# A robust workflow to benchmark deconvolution of multi-omic data

**DOI:** 10.1186/s13059-025-03897-9

**Published:** 2025-12-17

**Authors:** Elise Amblard, Vadim Bertrand, Hugo Barbot, Luis Martin Peña, Slim Karkar, Florent Chuffart, Mira Ayadi, Aurélia Baurès, Lucile Armenoult, Yasmina Kermezli, David Causeur, Jérôme Cros, Yuna Blum, Magali Richard

**Affiliations:** 1 TIMC, UMR 5525, Univ. Grenoble Alpes, CNRS, Grenoble, France; 2 IRMAR, UMR 6625 CNRS, Institut Agro Rennes Angers, Rennes, France; 3 IAB, Univ. Grenoble Alpes, CNRS UMR 5309, INSERM U1209, La Tronche, France; 4 Programme Carte d’Identité des Tumeurs, Ligue Nationale Contre Le Cancer, Paris, France; 5 Department of Pathology, AP-HP, Beaujon Hospital, University of Paris Cité, Clichy, France; 6 IGDR, UMR 6290, ERL U1305, Equipe Labellisée Ligue Nationale contre le Cancer, University of Rennes, CNRS, INSERM, Rennes, France; 7 LIG, Univ. Grenoble Alpes, CNRS, Grenoble INP, Grenoble, France; 8 IGBC, University Bordeaux, CNRS, IBGC, UMR 5095, Bordeaux, France

## Abstract

**Background:**

Tumour heterogeneity significantly affects cancer progression and therapeutic response, yet quantifying it from bulk molecular data remains challenging. Deconvolution algorithms, which estimate cell type proportions in bulk samples, offer a potential solution. However, there is no consensus on the optimal algorithm for transcriptomic or methylomic data.

**Results:**

Here, we present an unbiased evaluation framework for the first comprehensive comparison of deconvolution algorithms across both omic types, including reference-based and -free approaches. Our evaluation covers raw performance, stability, and computational efficiency under varying conditions, such as gene dependencies, missing or additional cell types and diverse sample compositions. We apply this framework across multiple benchmark datasets, including a novel multi-omics dataset generated specifically for this study. To ensure transparency and re-usability, we have designed a reproducible workflow using containerization and publicly available code.

**Conclusions:**

Our results highlight the strengths and limitations of various algorithms, and provides practical guidance for selecting the best method based on data type and analysis context. This benchmark sets a new standard for evaluating deconvolution methods and analysing tumour heterogeneity.

**Supplementary Information:**

The online version contains supplementary material available at 10.1186/s13059-025-03897-9.

## Background

Bulk transcriptome and methylome are routinely measured in the clinic to diagnose and classify cancer patients. However, these data are usually analysed in a way that does not account for intra-tumour heterogeneity, i.e. the fact that a tumour sample is composed of different cell types [1]. However, intra-tumour heterogeneity is a critical parameter as it affects tumour evolution and its response to treatment [2, 3]. It is possible to extract this information from bulk data with deconvolution tools that aim at predicting the proportions of the different cell types present in the sample [1, 4]. Deconvolution can also be used to quantify heterogeneity in non-cancer data, but deconvolving cancer tissues is the most classical use case in the literature. Nevertheless, there is no strong consensus on the best method to use [5, 6]. Second, as there is no multi-omic benchmark to our knowledge, it is not known what is the easiest omic to analyse with respect to the deconvolution task.

There are two classes of deconvolution algorithms. The supervised (or reference-based) methods use a matrix of reference profiles to estimate cell type proportions, whereas the unsupervised (or reference-free) methods estimate simultaneously the proportions along with reference molecular profiles of each cell type. In the supervised class, the quality of the references is key for the deconvolution performance [7, 8]. Critical points include the fact that the cells in the references should preferably come from the same tissue context as those to be deconvoluted (in vivo or in vitro), and that they should contain the expected cell types [6, 7, 9]. More precisely, most supervised algorithms cannot handle missing cell types. One solution implemented by few methods, called semi-supervised methods, is to allow the prediction of an unknown component [10]. On the other hand, unsupervised methods do not rely on references, avoiding associated challenges, while major disadvantages include the greater complexity of the problem and the difficulty in identifying the cell types retrieved during deconvolution. As a matter of fact, it has been shown that the components obtained after deconvolution are likely a linear combination of cell types present in the samples [2, 11].

Single-cell-based methods could overcome deconvolution-related issues by allowing a straightforward quantification of intra-tumour heterogeneity. Nonetheless, integrating such technologies into the standard of care for hospital patients is not yet feasible due to their time-consuming nature and high costs. Moreover, single-cell technologies do not capture all cell types with the same probability, blurring the estimation of the cellular composition [12]. Conversely, bulk technologies have been used for a long time at the hospital, and focusing on the analysis of these data will also allow to leverage the massive amount of data already generated. Nonetheless, it is possible to take advantage of both single-cell and bulk technologies. The strategy is to sequence the single cells of few samples to generate reference profiles from the same context and use them for supervised deconvolution of the other samples. We did not include single-cell-based deconvolution methods, which are more recent than bulk-based ones, for two main reasons. First, the properties of a bulk transcriptome differ significantly from those of a single-cell transcriptome, owing to variations in tissue processing and RNA enrichment protocols [13]. As a result, using single-cell references can be misleading compared to bulk references. Indeed, a previous benchmark showed that second generation single-cell-based methods did not outperform state-of-the-art bulk-based tools [14]. Second, there are no single-cell-based DNAm deconvolution methods. However, second generation single-cell-based methods are powerful and will likely lead to improved deconvolution methods [14, 15]. Another lead is to leverage bulk multi-omic approaches, which is the motivation of this benchmark.

As of today, there is a humongous amount of deconvolution algorithms available. Many benchmarks have been published to help bioinformaticians choose the best tool as a function of their data (Table 1). Still, current benchmarks suffer from several pitfalls: most of them include fewer than 10 methods, they do not all confirm rankings on real-world datasets (termed silver-standard datasets) and none except one provides a single comprehensive ranking. Only few studies investigated supervised and unsupervised methods simultaneously, and all of them studied a single omic: either transcriptomic or methylation alone. Lastly, there is a high discrepancy between these benchmarks, possibly because of the inconsistency of the metrics used to measure performance [6].

**Table 1:** Characteristics of selected benchmark studies

Benchmark ID	Modality	Methods (#)	Class	Metrics	Gold-standards	Silver-standards
Avila Cobos et al. [7]	RNA	20		RMSE, Pearson correlation	✓	✓
Nadel et al. [8]	RNA	12	Supervised	AE, Pearson correlation	✓	✓
Zhang et al. [16]	RNA	3	Supervised	RMSE, MAE, MAPE, sMAPE, Pearson correlation	✓	✓
Jin and Liu [17]	RNA	11	Both	mAD, Pearson correlation	✓	✗
Sturm et al. [18]	RNA	7	Supervised	Pearson correlation	✓	✓
Our framework	RNA	16	Both	RMSE, MAE, Pearson correlation, Overall comprehensive score	✓	✓
Decamps et al. [19]	DNAm	3	Unsupervised	RMSE, MAE, Pearson correlation	✓	✓
Teschendorff et al. [20]	DNAm	4	Supervised	RMSE, Pearson correlation	✓	✓
Song and Kuan [21]	DNAm	8	Both	RMSE, sMAPE, Spearman correlation	✓	✗
Our framework	DNAm	11	Both	RMSE, MAE, Pearson correlation, Overall comprehensive score	✓	✓

*AE* Absolute Error, *RMSE* Root Mean Square Error, *MAE* Mean Absolute Error, *MAPE* Mean Absolute Percentage Error, *sMAPE* Symmetric MAPE, *mAD* mean Absolute Deviation

In our benchmark, we addressed those pitfalls. We built a robust workflow to systematically rank and evaluate deconvolution methods. We included 20 algorithms, supervised or unsupervised, designed for transcriptome or methylome data. We tested all methods on 3 types of data: in silico simulations, in vitro mixes and in vivo data, including a new multi-omic dataset. We studied various aspects of a method’s performance: (i) raw performance which measures errors in the prediction of the proportions, (ii) stability, and (iii) execution time. Based on those 3 categories, we provide an overall score per method along with intermediate scores, and p-values. We evaluated the effect of gene dependence, missing or extra cell types in supervised deconvolution, and of data dispersion and size in unsupervised deconvolution. We also analysed the methods’ performance in terms of rare cell type detection. We related our ranking with findings from previous benchmarks. We provide (i) a GitHub repository with all the codes needed to reproduce the analysis performed in the paper, along with (ii) an Apptainer container to run all methods included, and (iii) guidelines to choose a deconvolution method among those included here, as a function of the data to analyse. The repository and the container have been designed with flexibility in mind, allowing the inclusion of new datasets and methods.

## Results

**Fig. 1:**
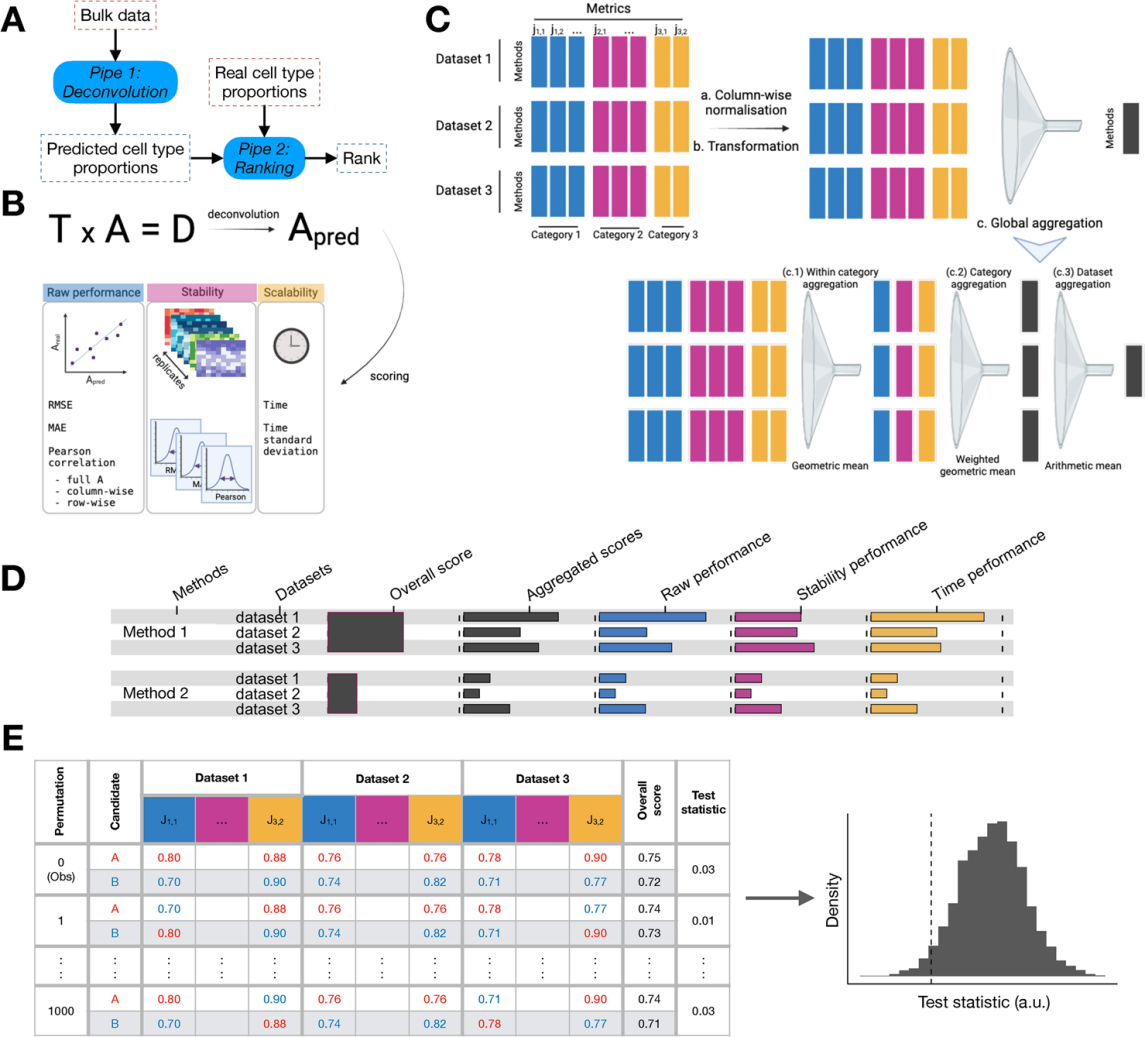
A comprehensive benchmark pipeline evaluates several aspects of the deconvolution performance. **A** The pipeline is done in 2 steps: a deconvolution step and a ranking step. **B** The deconvolution step outputs an estimation of the proportions which is used to evaluate a method’s raw performance and stability, along with its running time. **C** Metrics aggregation procedure, recapitulating a series of metrics from different natures across several datasets and yielding a single overall score per method. The different natures of the metrics impose to first normalize and transform the scores such that they all lie between 0 and 1, with 1 being the best possible value. **D** Example of an output from the 3-steps global aggregation procedure with the display of intermediate scores. **E** Permutation test to compute p-values

Our benchmark is a combination of two pipelines (Fig. 1A). The first pipeline performs the deconvolution task, the second the ranking task. The ranking pipeline has been designed to compare extensively deconvolution algorithms.

### The deconvolution pipeline is exhaustive, reproducible and flexible

We simulated 7 datasets (3 multi-omic, 3 RNA, 1 DNAm datasets), termed gold-standard, based on the bulk molecular profiles of pure cell types (Table 2). Using simulations allowed us to easily evaluate the performance of deconvolution methods as the ground truth is readily available from the simulation process. On the contrary, real datasets’ ground truth is usually not known, or is accessible only via proxies such as FACS or imaging. Another advantage of simulated datasets is the possibility to test the impact of data characteristics on the quality of the deconvolution. Here, we tested the effect of the number of samples ($n=30,120$) and of the diversity in sample composition with three levels of dispersion. Despite those benefits, the drawback of using simulations is that the results of the benchmark might not comply with real-life scenarii. Hence, we also analysed real datasets to confront our rankings (Table 2). We added gold-standard in vitro mixes: 1 multi-omic, 1 DNAm and 1 RNA datasets; as well as 2 RNA and 2 DNAm silver-standard in vivo datasets. in vitro mixes are real-life datasets, with the advantage of having the ground truth, but there are only few such datasets available.

**Table 2:**
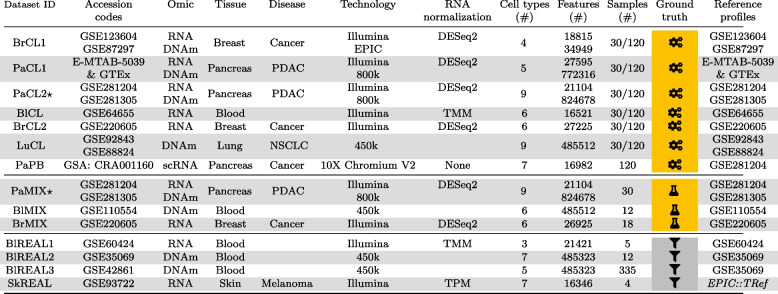
Table of gold- and silver-standard datasets used in the benchmark. The penultimate column refers to the type of the ground truth matrix: for gold-standard datasets, the ground truth is exact and represented by the symbols for simulations and for the in vitro mixes, while it is a proxy, usually measured by FACS and represented by the symbol , for silver-standard datasets. The last column indicates the reference profiles used for supervised deconvolution. The in silico datasets are under the acronym CL (Cell Line) or PB (pseudo-bulk), the in vitro datasets under the acronym MIX and the in vivo under the acronym REAL. New datasets generated for this study are flagged with the symbol $\star$. For the PaCL1 dataset, more details on how to download it are on the website https://deconbench.github.io/Benchmark_datasets.html. PDAC: pancreatic adenocarcinoma, NSCLC: non-small-cell lung cancer

We analysed fourteen gold- and silver-standard datasets (Table 2) with twenty deconvolution methods (see Methods and Additional file 1: Tables S1, S2) in the deconvolution task. All methods have been run on a high performance computing cluster, using Snakemake [22] and an Apptainer container (see Methods) to enforce reproducibility and re-usability. Owing to these tools, the deconvolution pipeline is flexible in order to allow for additional methods and/or datasets. The input of this first pipeline is a dataset and a method and the output is the estimation of cell type proportions along with the running time (Fig. 1A, B).

We tested several deconvolution methods designed for either of the two omics we included, namely the methylome and transcriptome, comprising different algorithmic designs: Bayesian-, or Least Squares-based (LS) for supervised methods, ICA- or NMF-based for unsupervised ones. After a thorough review of the literature (Additional file 1: Tables S1, S2), we included 12 supervised methods in our deconvolution pipeline ($n=7$ for transcriptomic, $n=1$ for methylation data, $n=4$ for both omics), and 8 unsupervised methods ($n=2$ for transcriptomic, $n=3$ for methylation data, $n=3$ for both omics) (see Methods, paragraphs “Supervised deconvolution algorithms” and “Unsupervised deconvolution algorithms” for an exhaustive list of the algorithms we tested).

### The ranking process enables unambiguous comparisons of algorithms

The ranking pipeline takes as input the estimation of the proportions and the running time from the deconvolution pipeline. For each prediction, we computed five performance metrics along with the running time, named primary metrics, and six additional secondary metrics when replicates were available (Fig. 1B and Methods): error metrics (RMSE, MAE and Pearson correlations) to measure the raw performance, standard deviation of those error metrics to measure the stability, and the running time and its stability. We selected metrics that are routinely used in other benchmarks (Table 1). Those metrics are aggregated by the pipeline into a single overall score (Fig. 1C and Methods). The output of this second pipeline is an overall benchmark score associated with p-values for the pairwise comparisons of methods, along with the intermediate scores (Fig. 1D). We also display intermediate scores in order to better understand why a method has a good or bad performance (Fig. 1D). Finally, we compute p-values along with the overall score (Fig. 1E and Methods).

We designed several ranking processes and tested them according to empirical criteria defined in [23] (Additional file 1: Fig. S1 and Methods). We chose the process $S_{consensus}$, as it was the most generalizable one, based on the generalisation criterion (Additional file 1: Fig. S2E, F). This criterion quantifies the stability of the ranking with respect to the exclusion of few metrics, which is a desired property. The consensus process averages the outputs of three other processes $S_{raw}$, $S_{rank}$ and $S_{topsis}$. $S_{raw}$ merges the different metrics after normalization, $S_{rank}$ merges the ranks computed from each metric, and $S_{topsis}$ merges the TOPSIS scores [24] (see Methods for more details). Besides, we observed that the top methods elected by each process remained the same, which serves as a proof of the robustness of our ranking processes and their results (Additional file 1: Fig. S2A, B, C, D, F). The codes to run the ranking process are available and can be modified to add new metrics or modify the aggregation process (See Methods).

### RLR is the best supervised method irrespective of the type of omic

We first analysed data simulated with bulk reference profiles and applied deconvolution to 3 versions of each expression/methylation matrix: (i) without feature selection, with feature selection performed (ii) by selecting the 1,000 most variable features or (iii) by TOAST and selecting 1,000 features [25]. TOAST works by iteratively doing ICA followed by a selection of the features differentially expressed across components. For each deconvolution method, we selected the version of the expression matrix yielding the best overall benchmark score (Additional file 1: Fig. S3). Overall, the strategy with no feature selection is ranked best in 63% (10/16) of the cases, and it is especially true for RNA data (8/11). The second best feature selection is TOAST (4/16). We observed that increased performance after feature selection is partly due to a substantially decreased running time, while stability is reduced (Additional file 1: Fig. S4). Additionally, we tested the impact of the type and number of genes selected for RNA deconvolution. We compared a situation with only 1000 highly variable genes (HVG), or HVG supplemented with $1\%,5\%,10\%$ of either outlier genes (defined as genes with the highest median expression, see Methods) or randomly selected genes. We observed that most methods benefit from including more genes, with outlier genes seemingly more meaningful deconvolution-wise than random genes (Additional file 1: Fig. S5).

**Fig. 2:**
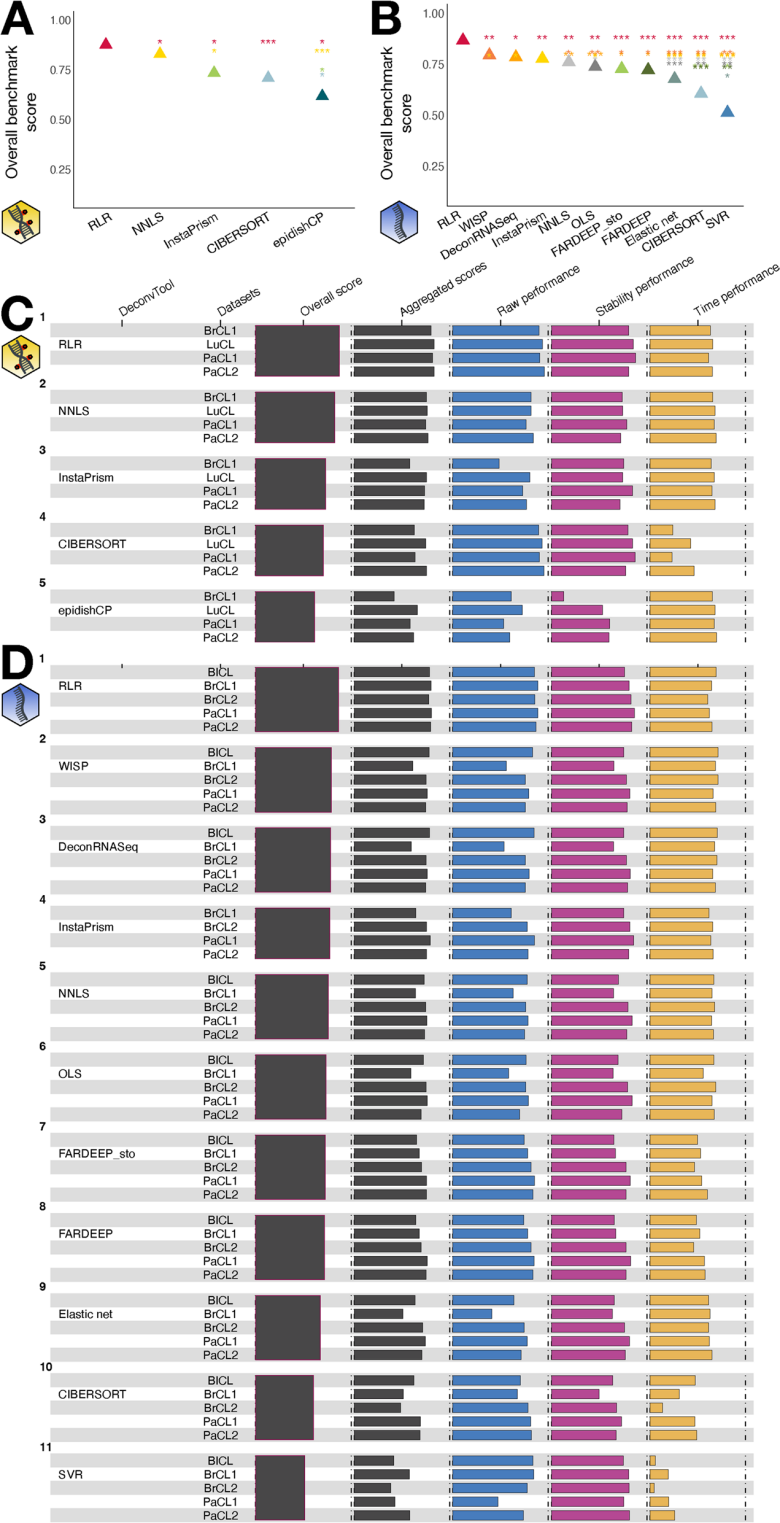
Benchmark scores of supervised deconvolution methods. Methods have been applied to the methylation (**A**, **C**) or the transcriptome (**B**, **D**). **A**, **B** Statistical significance of the differences in overall benchmark scores. Overall benchmark scores are shown with triangle symbols, and one-sided p-values between two methods are shown with stars, the colour and y-value relating to the supposedly better method, the x-value relating to the supposedly worse method. Stars follow the convention: (*) for p-values below 0.05, (**) below 0.01, (***) below 0.001. **C**, **D** Overall and intermediate scores. Each row represents a method run on the different simulations. The third column is the overall benchmark score, the forth the aggregated score per dataset, and the fifth to seventh columns the scores relating to each of the three categories, averaged per dataset. The yellow DNA icon stands for methylation data, the blue one for transcriptome data

Figure 2 presents the result of the benchmark for supervised methods on simulated data, displaying the p-values along with overall and aggregated scores. RLR is one of the 4 methods that can be used on both omic types and it has consistently been the best method in a significant fashion (Fig. 2A, B). RLR is also one of the method yielding the less variation in performance from one dataset to another (Additional file 1: Fig. S6A, C). Those good results can be explained by the fact that the method is robust to outliers, while other least-squares-based methods also have good scores. In contrast, the performance dependency with respect to the dataset increases with decreasing overall score, especially for the methylome. For all datasets, RLR displays the best aggregated scores (Additional file 1: Fig. S7A, C) as well as the best scores for the raw performance (as shown also in [26] for DNAm and in [7] for RNA) and the stability categories (Fig. 2C, D). Additionally, our results are consistent with the ranking obtained in [26] where the authors used Spearman correlation. While our framework relied on mean expression profiles for convolution-based simulations, thereby ignoring the biological variability within individual cell types, we tested the robustness of rankings to such variability by generating additional simulations. Specifically, we constructed pseudo-bulk samples from a pancreatic single-cell RNAseq dataset [27], referred to as PaPB (see Methods and Table 2). These pseudo-bulk samples were deconvolved using the same reference as the PaCL2 dataset. We then compared the rankings derived from PaPB to those obtained on PaCL2 and other bulk-based convolution simulations, and found them to be largely consistent across simulation conditions (Additional file 1: Fig. S8A), indicating that the results are robust to biological variability.

### The performance of unsupervised methods strongly depends on the dataset

While there is a consensus on the best method for both omics and across datasets in the supervised setting, this is not the case for unsupervised algorithms. Again, we retained the feature selection strategy which gave the best overall score for each deconvolution algorithm (Additional file 1: Fig. S3). Regarding the impact of the type and number of genes selected for RNA deconvolution, we observed that CDSeq and PREDE perform best when run on HVG only, while the 3 other methods have better scores when run with more genes, be it outliers or random ones (Additional file 1: Fig. S5B).

**Fig. 3:**
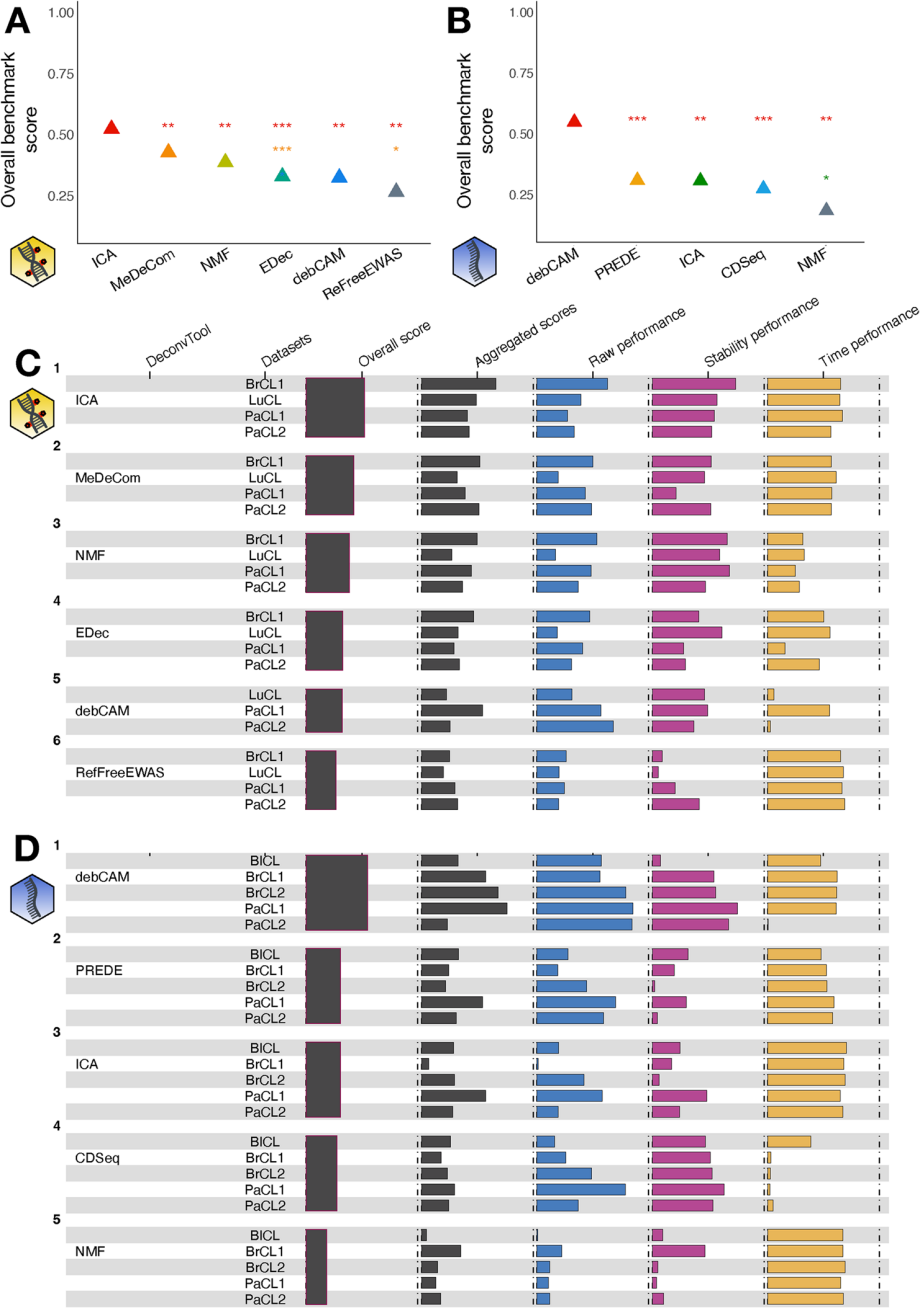
Benchmark scores of unsupervised deconvolution methods. Methods have been applied to the methylation (**A**, **C**) or the transcriptome (**B**, **D**). **A**, **B** Statistical significance of the differences in overall benchmark scores. **C**, **D** Overall and intermediate scores

For methylation data, ICA has the highest overall score, and performs significantly better than all other methods (Fig. 3A, C), but it displays contrasted results across datasets: it is the best algorithm for 50% of the datasets (2/4), and the second best for PaCL2 (Additional file 1: Fig. S7B). On the contrary, the best methods are debCAM for PaCL1 and MeDeCom for PaCL2, and the method yielding the most stable results across datasets is RefFreeEWAS (Additional file 1: Fig. S6B).

For transcriptomic data, the best method is debCAM (Fig. 3B, D), as it performs particularly well in all except the PaCL2 dataset compared to the other methods (Additional file 1: Fig. S7D). The performance varies greatly from one dataset to another, except for CDSeq (Additional file 1: Fig. S6D). We observed that the performance of unsupervised deconvolution depends on the dataset much more strongly than for supervised methods (Additional file 1: Fig. S6). We also evaluated the performance of unsupervised methods on the pseudo-bulk dataset PaPB. Again, we observed that, although performance depends on the dataset, rankings are almost conserved with only marginal differences (Additional file 1: Fig. S8B). All these observations shed light on the difficulty of reaching a consensus on the best deconvolution tool. Additionally, supervised methods perform better than unsupervised ones, as already shown in the literature [28, 29].

### RNA and DNAm deliver comparable performance across method designs

**Fig. 4:**
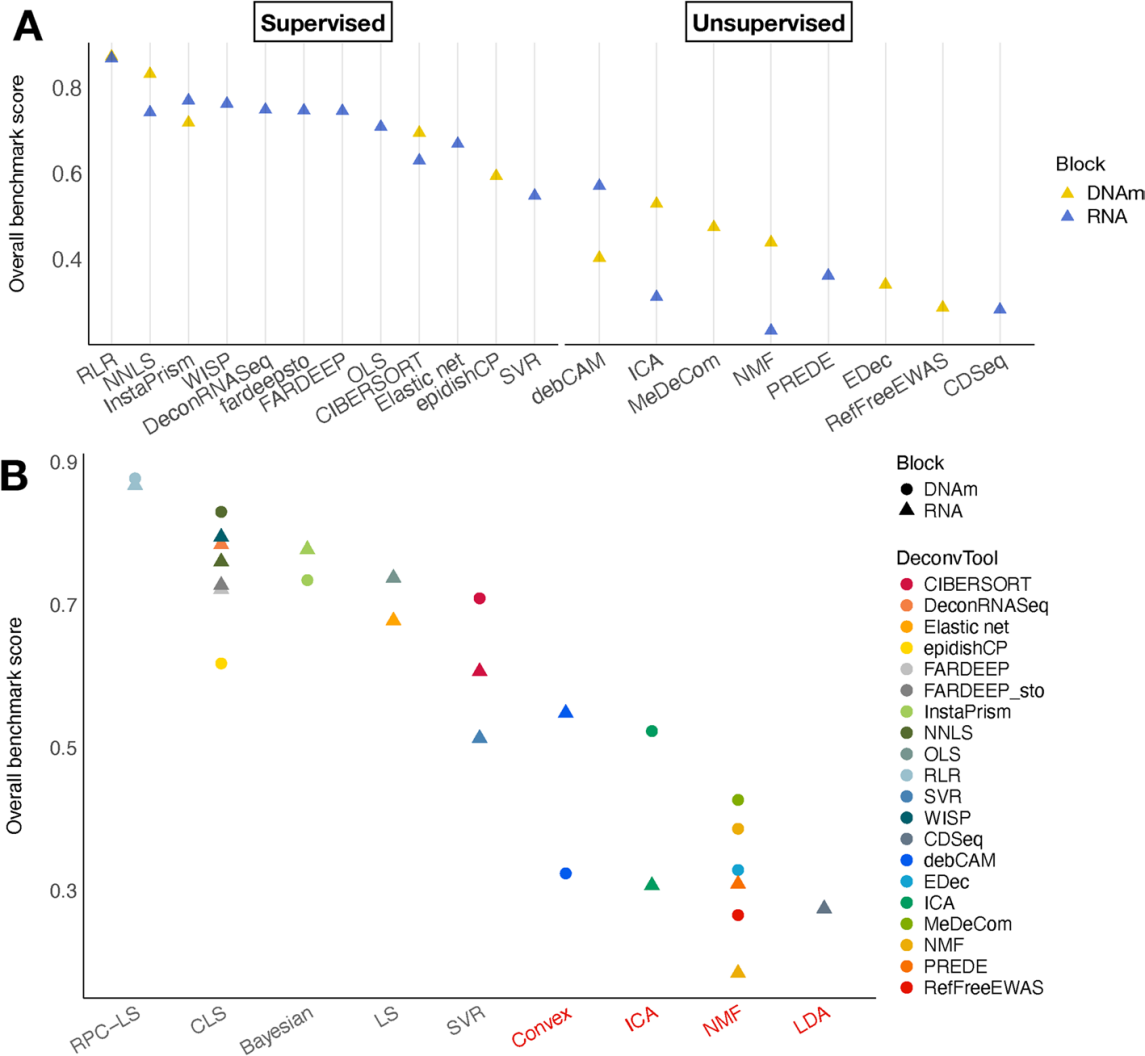
Comparison of the deconvolution performance for each omic. **A** Overall benchmark score of all methods included in the benchmark, ranked on the three multi-omic in silico datasets. **B** Overall benchmark scores of all methods sorted by design (i.e. type of algorithm), with unsupervised designs labelled in red. LS: Least Squares. CLS: Constrained LS. RPC: Robust Partial Correlation. SVR: Support Vector Regression. LDA: Latent Dirichlet Allocation

Since our objective was to explore and compare multi-omic data, we wanted to determine the easiest omic to deconvolve (if any), based on the three simulated multi-omic datasets. We observed that for the best method (RLR) achieved similar results for both DNAm and RNA (Fig. 4A). However, DNAm showed a slight advantage for two-thirds of the methods applicable to both omics: NNLS, CIBERSORT, ICA, and NMF performed better on DNAm, whereas InstaPrims and debCAM performed better on RNA. Overall, it is challenging to make a definitive recommendation for preferring one omic over the other. In the supervised category, RLR applied to either DNAm or RNA data emerged as the best choice. The second-best method was NNLS on DNAm; however, beyond that, top performances were generally associated with RNA deconvolution, likely reflecting extensive methodological development efforts over the past decade. In the unsupervised category, the highest performance was obtained for RNA analyzed with debCAM, although the next best methods (ICA, MeDeCom, and NMF) were associated with DNAm analysis.

We aimed to determine whether the observed performance ranking was driven by superior algorithmic design or by intrinsic differences between the two omics analysed. We tested which specific designs were the most competitive in our benchmark. We summarized in Additional file 1: Fig. S9 the type of model, and whether the sum-to-one constraint on the proportion matrix was implemented in the form of an equality or inequality. Since too few methods used an inequality constraint, we focused on analysing the impact of the type of model. The most popular approaches are NMF for the unsupervised class and LS for the supervised class (Additional file 1: Fig. S9). However, the most efficient ones were the convex analysis and ICA for unsupervised methods and robust LS for supervised methods (Fig. 4B). While it provides hints for future algorithms to improve the quality of the deconvolution, we also observed that for a given design applied to both omics, the best benchmark score is attributed to DNAm in 71% of the cases (5/7). To conclude, each approach seems to perform best on a specific omic, probably relating to the property of the data itself.

### Simulation parameters affect the deconvolution performance

We tested two simulation parameters, namely the number of samples ($n=30,120$) and the dispersion factor ($\alpha _0=3,10,30$), related to the amount of heterogeneity in the proportions: the higher $\alpha _0$, the lower the dispersion (see Methods). We considered the baseline simulation as the one defined by $n=120,\alpha _0=10$, and we evaluated the impact on the overall score, computed on all in silico datasets, of varying the number of samples or the dispersion, mimicking biological noise, in the unsupervised class of methods, without feature selection.

**Fig. 5:**
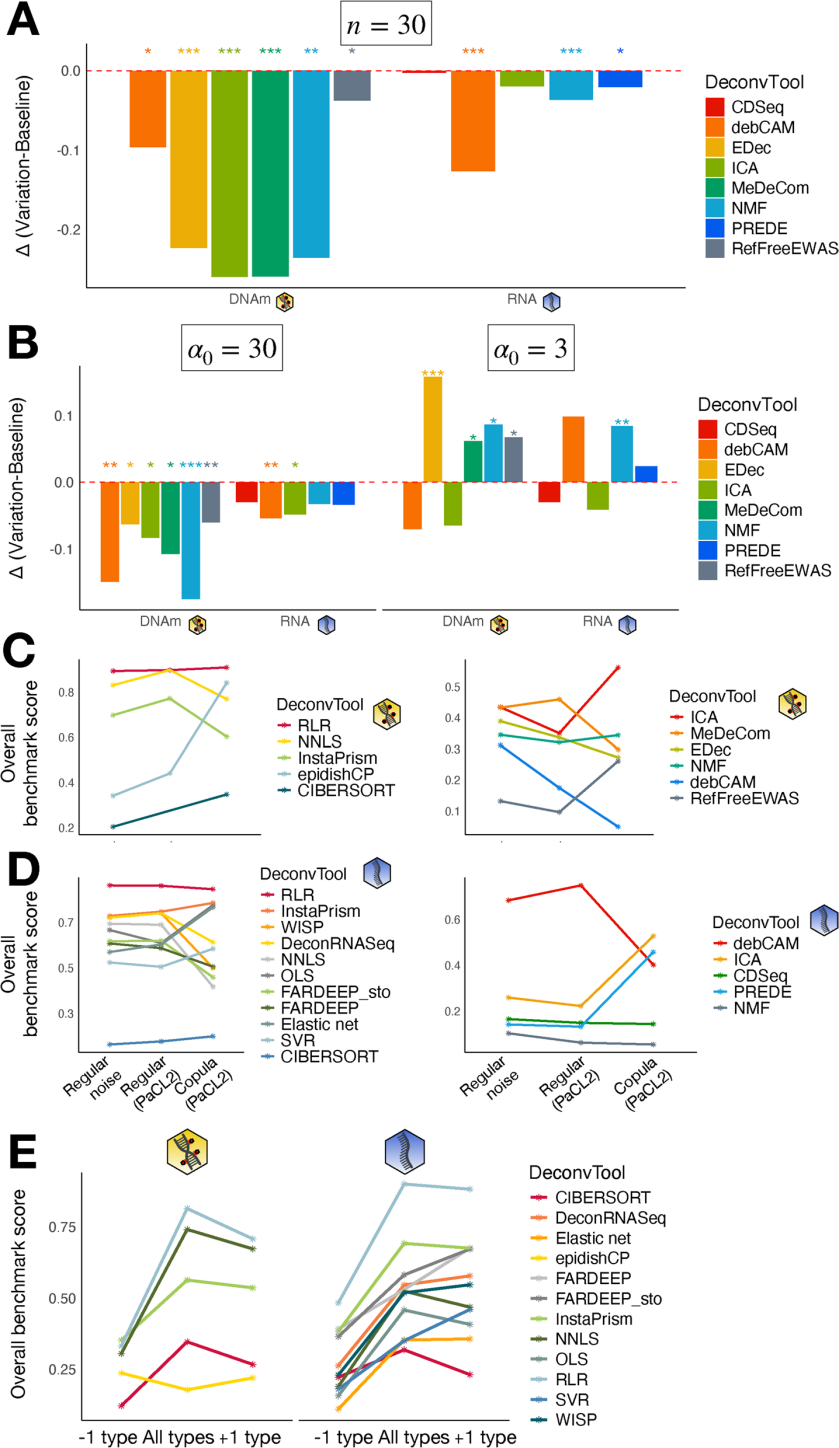
Impact of simulation parameters. **A** Comparison of overall scores for unsupervised methods between the baseline simulation ($n=120$) and simulations with fewer samples ($n=30$). **B** Comparison of overall scores for unsupervised methods between the baseline simulation ($\alpha _0=10$) and simulations with less ($\alpha _0=30$, left panel) or more ($\alpha _0=3$, right panel) dispersion. Methods for which the difference in scores is significant are flagged with stars indicating the p-value. **C, D** Overall scores of regular Gaussian/Negative Binomial noise versus copula noise on methylation data (**C**) and transcriptome (**D**) data (left: supervised, right : unsupervised). **E** Overall scores of supervised methods in the case of a missing (“−1 type”) or extra cell type (“+1 type”) in the reference matrix versus the correct cell types (“All types”). The yellow DNA icon stands for methylation data, the blue one for transcriptome data. We display overall rather than aggregated scores —i.e. a single score per method, without error bars. This is due to the ranking procedure

For the number of samples, we observed that unsupervised methods always perform worse when there are fewer samples than at baseline (Fig. 5A), especially for the methylation where score differences are significant for all methods. This trend is expected and has been shown before [19].

Unsupervised methods are also sensitive to the dispersion parameter. Indeed, all methods performed worse when there was less dispersion ($\alpha _0=30$) than in the baseline, and it is particularly true for the methylation where the difference in scores is always significant (Fig. 5B). In the $\alpha _0=3$ situation, many methods perform better than the baseline except for ICA, and debCAM for methylation data and CDSeq for transcriptomic data, although the deterioration of the score was never significant. This improvement in performance makes sense as well, as more dispersion means more information (Additional file 1: Fig. S10): in extreme situations where the $\alpha _0$ factor is very large, this would cause all samples to have the same composition [19]. In the case where one expects its samples to have a similar composition, the results from our benchmark indicate that deconvolution is less affected with RNA than with DNAm data.

Beyond the dispersion parameter, we also studied the effect of noise type on the performance, with a focus on modelling feature dependencies. We generated additional simulations incorporating a copula-based noise, which captures the covariance structure between features (see Methods). This approach requires real data to estimate the Copula, therefore it was only implemented for PaCL2, using the Copula derived from PaMIX. The same supervised and unsupervised methods were run on these simulations, without any feature selection. We then compared the resulting rankings to those obtained using standard noise models (Gaussian or Negative Binomial, referred to as “Regular noise” in the figure) across all simulated datasets or specifically on PaCL2 (Fig. 5C, D). As expected, the performances depend on the type of noise. Among supervised methods however, RLR remains the best tool (Fig. 5C, D left panels). For unsupervised approaches and as already previously observed, there is less consensus (Fig. 5C, D right panels). While debCAM retains good performances on the copula simulations for RNA data, no consistent trend was observed for DNAm data.

We evaluated the effect of removing or adding a cell type in the reference profile matrix during supervised deconvolution without feature selection (see Methods). As expected, the optimal performance is mostly met when the reference profiles comprise only the cell types present in the samples (Fig. 5E). This is true for all methods and both omics except for epidishCP for DNAm and 5 methods for RNA analysis. However, the differences in overall benchmark scores are negligible. Moreover, we observed that in the case of an extra or missing cell type in the reference, rankings are fairly well conserved, especially for top methods. The most robust methods to an incorrect reference are epidishCP for DNAm data and CIBERSORT for RNA in the case of a missing cell type. In the case of an extra cell type, the most robust ones are FARDEEP_sto, DeconRNASeq and WISP (for RNA data) and InstaPrism (for DNAm data). On the other hand, methods which are the most sensitive to an incorrect reference are the methylation methods RLR and NNLS and the transcriptomic methods RLR, NNLS and InstaPrism, in the case of a missing cell type. As a control, we also evaluated the effect of adding a real cell type to the reference matrix of BrCL1, instead of a simulated one (see Methods). Interestingly, for BrCL1, including an additional cell type in the reference had little impact on supervised deconvolution performance, and the difference between using a real versus a simulated cell type was negligible (Additional file 1: Fig. S11).

### Rare inter-correlated cell type detection strongly depends on the deconvolution method

**Fig. 6:**
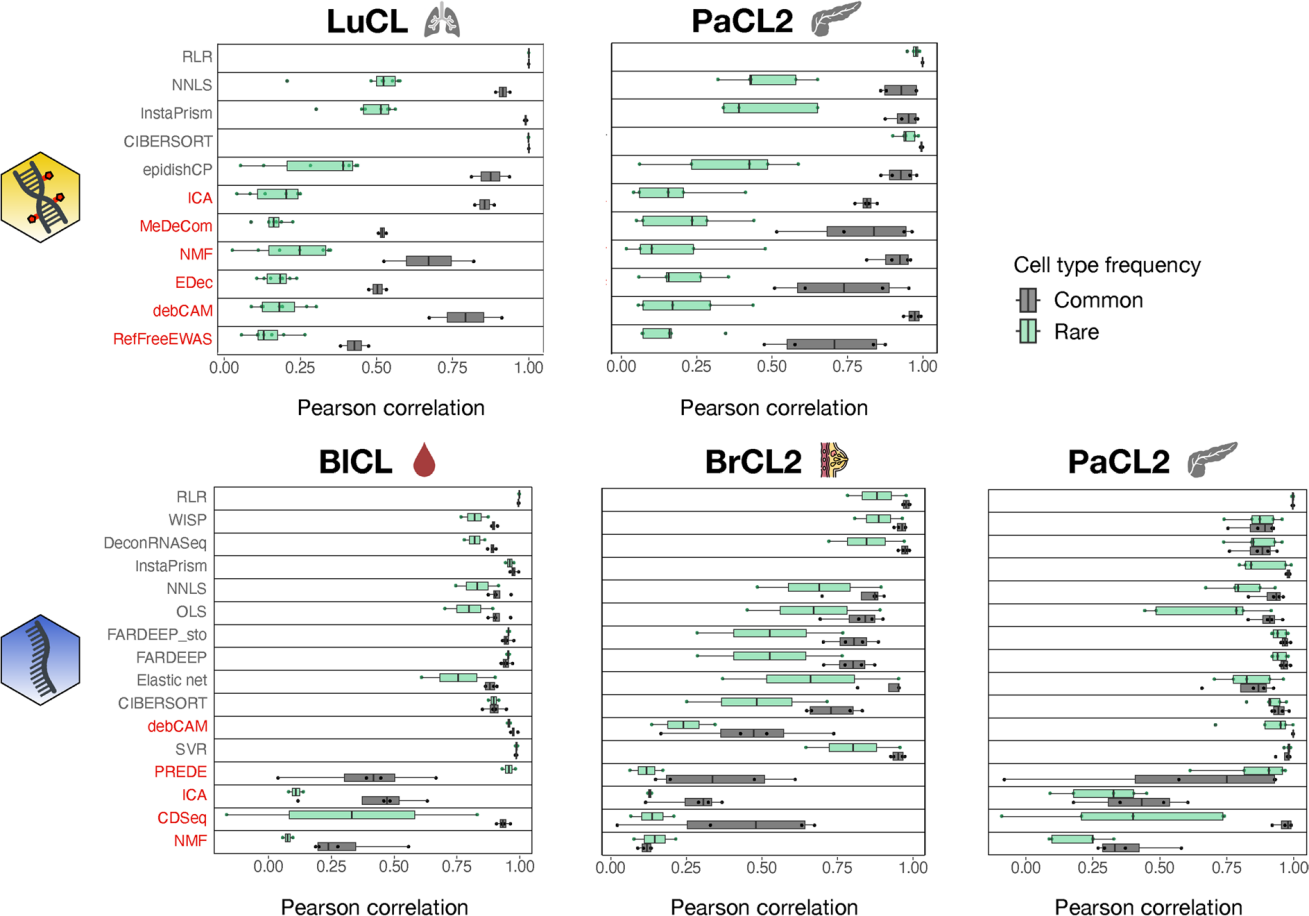
Efficacy to detect rare cell types. Cell type Pearson correlations for rare and common cell types were computed for each dataset containing rare types. Methods are ordered from top to bottom by their overall benchmark score for each omic type. Unsupervised methods are labelled in red. Number of rare types varies across datasets: $n=7$ for LuCL, $n=5$ for PaCL2, $n=2$ for BlCL and BrCL2. For the common types, $n=2$ for LuCL and $n=4$ for all other datasets. The yellow DNA icon stands for methylation data, the blue one for transcriptome data

Depending on the research question, the main goal could be to estimate rare cell types. In the specific case of oncology, it is important to quantify the immune infiltrate in the tumour microenvironment where these cells are usually lowly abundant. We defined as rare cell types in our simulations those with proportions $\alpha _i$ below 5% (Additional file 1: Table S3). It encompassed immune types across 4 datasets (3 simple-omic, 1 multi-omic). We investigated whether our methods, combined with their optimal feature selection option as defined from Additional file 1: Fig. S3, were fit to detect rare occurrences, based on cell type Pearson correlations. Starting with methylation deconvolution, we observed that all methods were less efficient to estimate the proportions of rare immune cell types compared to common ones except for RLR and CIBERSORT in the LuCL dataset (Fig. 6 upper panel). Apart from this particular case, there are only few methods for which the detection of rare immune cell types is not too degraded compared to the detection of common cell types (defined by a difference in mean correlation below 0.2 for all datasets): RLR and CIBERSORT, which are only supervised methods. On the other hand, methods for which the detection of rare cell types is strongly degraded (defined by a difference in mean correlation above 0.4) include NNLS, InstaPrism, epidishCP, ICA, NMF, and debCAM. Interestingly, the ability to detect rare immune types does not depend linearly on the overall ranking, nor on the rankings of each dataset (Additional file 1: Fig. S7), but rather on the class of the method: supervised methods are more efficient than unsupervised ones in detecting rare immune types. Our conclusions hold true when we only compared the efficacy to detect rare immune cell types: RLR and CIBERSORT are the best methods, independent of their relative performance on common cell types.

For RNA deconvolution, we also investigated performance degradation for the detection of rare immune cell types. With this omic type, the estimation of rare types is not systematically worse than the estimation of common types (Fig. 6 lower panel), with some rare types being more correctly estimated than common types: e.g. SVR and PREDE in both cancer datasets. There is also less agreement across datasets than for the methylation. However, the mean correlation is in most cases smaller for rare types than for common types (77%, 36/47). For transcriptomic data, 5 methods are not too degraded when detecting rare immune types: RLR, WISP, DeconRNASeq, NNLS and SVR. Conversely, and defining large degradation as a difference above 0.4, only CDSeq meets this criterion for the two cancer datasets. On an absolute basis, RLR is the best method across datasets to estimate rare immune cell types. Finally, and based on the analysis of the multi-omic dataset PaCL2, we observed that rare immune cell types detection is the most competitive with RNA data.

Since rare types included only immune cells in our cancer and haematologic datasets, we investigated whether their lower prediction accuracy could be explained by their similarity with other cell types. We observed that there is indeed a trend: the more a cell type is correlated with others, the more difficult it is to deconvolve, regardless of its abundance and particularly in DNAm data (see Methods and Additional file 1: Fig. S12A). For example, rare macrophages in PaCL2 are quite dissimilar from other cells transcriptome-wise and easy to deconvolve, while it is the opposite for endothelial cells. In general, immune cells are among the types that resemble the most to the other types (Additional file 1: Fig. S12B), which is likely to contribute to the difficulty of deconvolving them. To further disentangle the roles of abundance and similarity, we simulated a dataset with the BrCL2 references where fibroblasts were assigned to a proportion of $5\%$ and all other cell types were above that threshold (see Additional file 1: Table S3) and we investigated how accurate their estimate was. Despite their scarcity, fibroblasts, being the most distinct from other cell types, were still accurately estimated, achieving the highest Pearson correlation among all types and all methods (Additional file 1: Fig. S12B, S13). These findings support the conclusion that similarity along with rareness, rather than rareness alone, play a key role in deconvolution accuracy.

### In vitro and in vivo datasets recapitulate in silico ranking

**Fig. 7:**
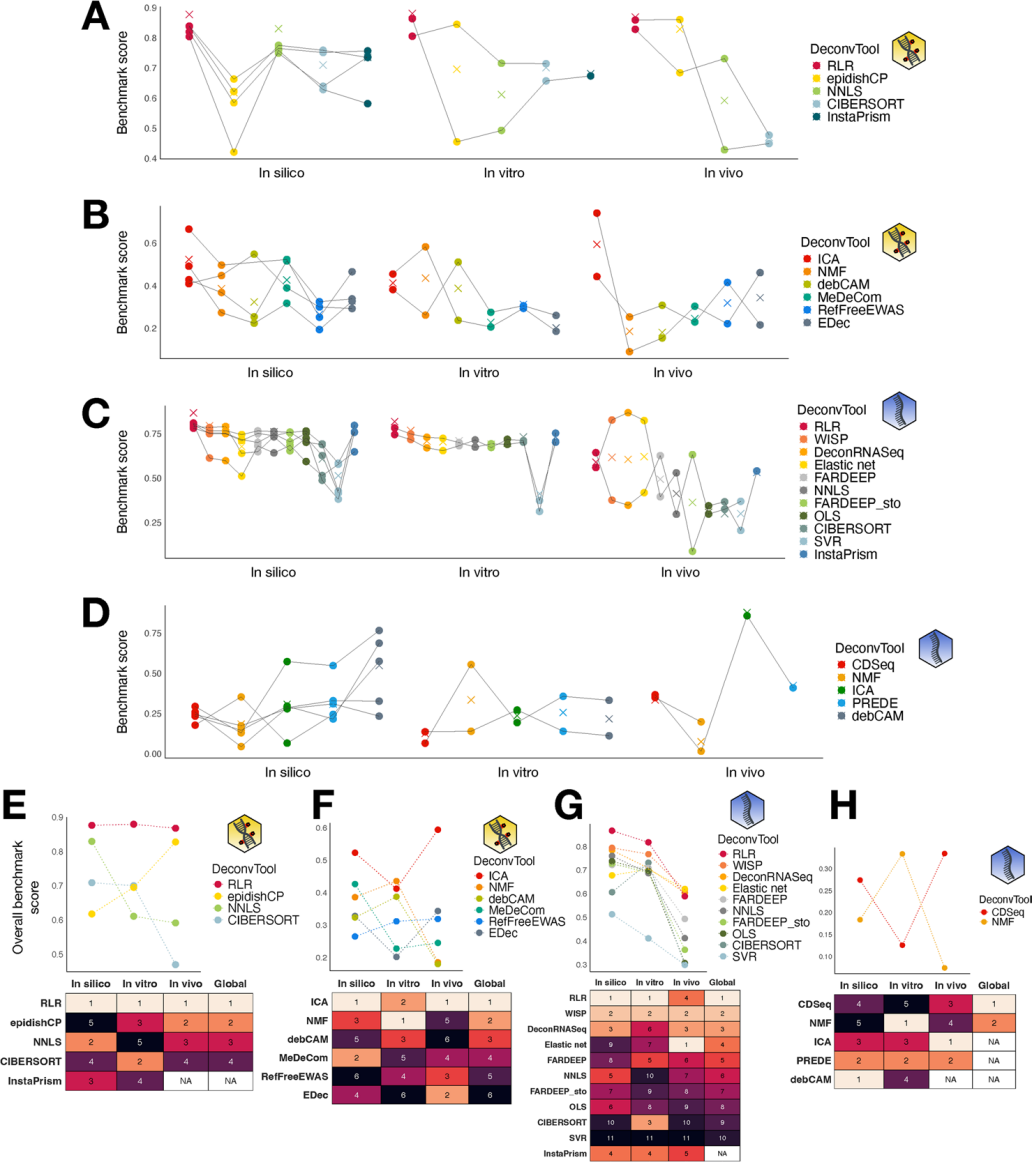
Rankings as a function of omic type, class and data source. **A**, **B**, **C**, **D** Overall and aggregated benchmark scores for all three sources, in the supervised (**A**, **C**) and unsupervised (**B**, **D**) settings. The aggregated scores are depicted by circles. Panels E to H display the overall score in each source and the respective rankings, ’global’ being the ranking as defined by the mean of the scaled overall scores for each method, in the supervised (**E**, **G**) or unsupervised (**F**, **H**) settings. Methods that were excluded from the comparison across data sources or did not run on all datasets of a given source have no ranking (NA in the ranking tables). The yellow DNA icon stands for methylation data, the blue single-strand one for transcriptome data

As mentioned earlier, simulations differ from real-life datasets and we need to ensure that our results are also valid for other data sources. We confronted in Fig. 7 rankings obtained via datasets from different sources: gold-standard in silico and in vitro datasets, and silver-standard in vivo datasets. We computed the ranking for each source independently of the others, as we do not want to compare the respective performance between sources but the ranking of the different methods itself. Notably, we cannot compute the secondary stability metrics for real datasets since we do not have replicates, so we used two different sets of metrics depending on the source (see Methods and Additional file 1: Fig. S2F). We also retained only the methods that ran on all in vitro and in vivo datasets, otherwise it would have favoured methods that were running only on a few datasets (Fig. 7A, B, C, D). Indeed, some methods were not able to deconvolve few real datasets, and we penalized it by excluding them in this comparison. We retained 10 out of the 11 DNAm methods and 12 out of the 16 RNA methods. In particular, SkREAL had too few samples ($n=4$), which caused several methods to fail (Additional file 1: Fig. S14). InstaPrism was excluded in both omics as it requires to define variable cell types, which we chose to be the cancer cell types; hence, it was run only on cancer datasets (see Methods).

Among supervised methylation methods, RLR remains the best method across all sources (Fig. 7E). For the unsupervised case, the best overall method is ICA (Fig. 7F).

In supervised RNA deconvolution, we obtained a coherent ranking. RLR and WISP are always in the top methods for each source (Fig. 7G). Major ranking shifts are observed for Elastic net, NNLS and CIBERSORT though. In the unsupervised case, we retained only 2 out of the 5 methods, making it difficult to challenge any observation (Fig. 7H).

This confirms the robustness of our ranking method, as well as the hegemony of some methods over others, such as RLR in the supervised class.

Using multi-omic multi-source datasets and methods, we revisited the comparison between DNAm- and RNA-based deconvolutions to assess the relative ease of deconvolution across these two omic types. A widespread assumption is that RNA-based deconvolution of in vitro and in vivo datasets is less accurate than DNAm-based. Indeed, RNA deconvolution estimates the fraction of RNA coming from each cell type (thus depending on cell size) which might differ from true cellular fractions [30], while the ground truth, as defined by FACS or in vitro proportions, reflects the true cellular fractions. Since our design should avoid this pitfall for simulated data, we checked whether methods that perform best on RNA in the case of simulations would improve on DNAm in the case of real data. Retaining only BrCL1, PaCL1, PaCL2, PaMIX and the blood in vivo datasets, we ranked CIBERSORT, InstaPrism, NNLS, RLR, debCAM, ICA, and NMF for each source, retaining the best feature selection based on Additional file 1: Fig. S3. We observed that among the methods that performed best on RNA for in silico data (namely InstaPrism and debCAM), debCAM was the only method with better results on DNAm for real datasets (Additional file 1: Fig. S15). The other methods already performed better on DNAm for simulated data. Interestingly, among those, all except NNLS showed in fact better results with RNA on at least one type of real data (mostly on in vivo datasets). These results suggest that cell size may not significantly impact deconvolution accuracy in our datasets.

### Our benchmark provides a robust and comprehensive overview of a method’s performance

**Fig. 8:**
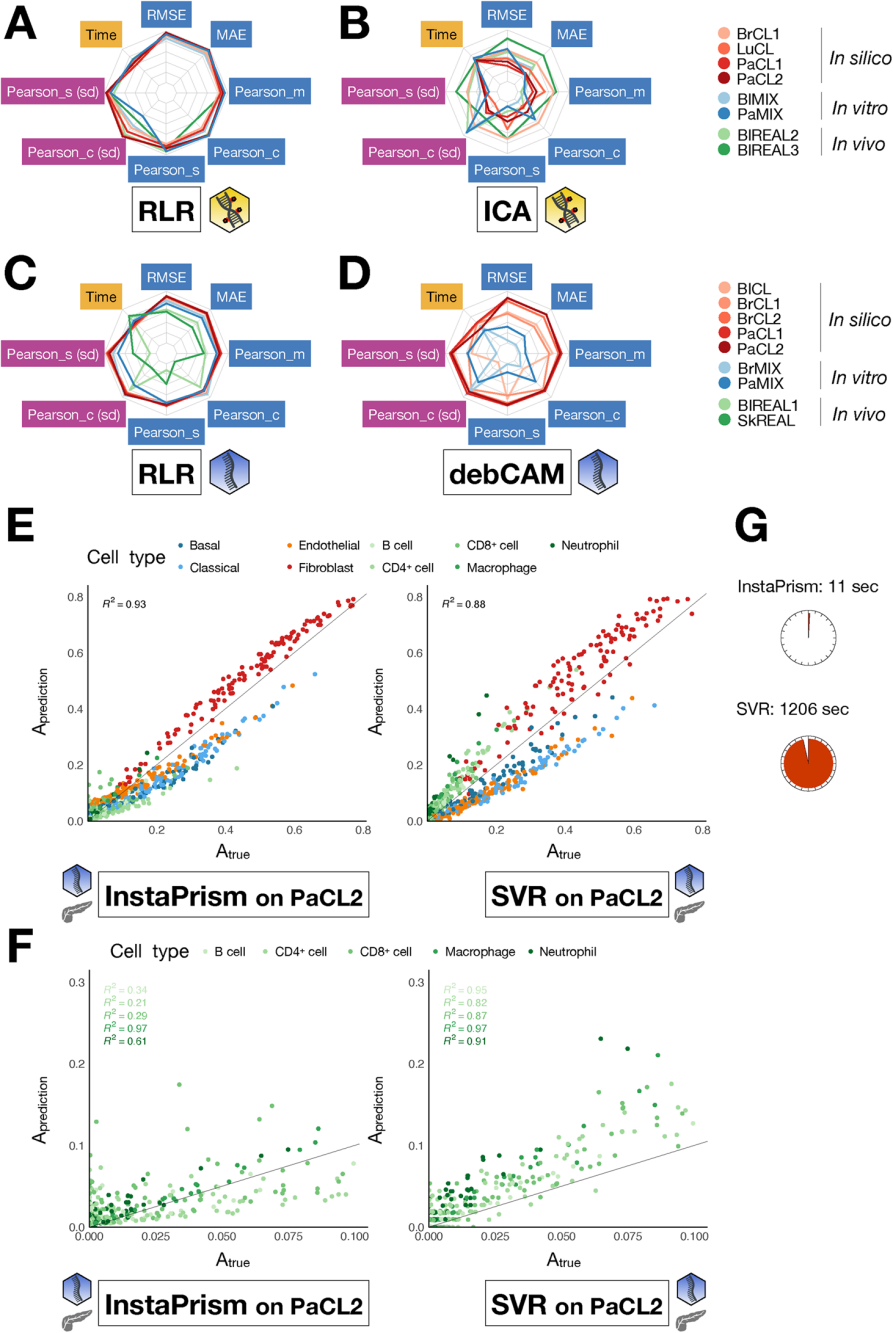
Detailed visualisation of methods’ performances. The benchmark allows to display several visual outputs. **A**, **B**, **C**, **D** Spiderplots of normalised-transformed primary metrics quantifying raw performance (blue), stability (violet) and scalability (yellow), for all dataset sources and for the best method in each class (supervised (**A**, **C**) and unsupervised (**B**, **D**)) and omic (methylation **A**, **B**) and transcriptome (**C**, **D**)), as determined by the in silico ranking. **E**, **F** Predicted proportions as a function of true proportions for one simulation of PaCL2, coloured by cell type; all cell types (**E**) or only rare types (**F**). **G** Running time for each algorithm. The yellow icon stands for methylation, the blue icon for transcriptome

Owing to our ranking process, we could unambiguously compare deconvolution tools. However, we can also look at intermediate and primary scores in order to better understand what makes a method efficient or not (Fig. 8A, B, C, D, Additional file 1: Fig. S16). For example, we observed that RLR performs well for all metrics in all DNAm datasets. We also observed that spiderplots of supervised methods looked more regular than for unsupervised ones, in terms of metrics and datasets. All those scores allowed us to come up with guidelines in terms of dataset’s easiness-of-deconvolution and algorithmic design’s strengths and weaknesses. It could also help choose a method as a function of a specific use case. As an illustration, we compared InstaPrism and SVR to deconvolve PaCL2. InstaPrism made fewer errors ($R^2=0.93$) than SVR ($R^2=0.88$) on the whole matrix, and ran 100 times faster, but it failed to correctly estimate rare proportions compared to SVR, except for macrophages (Fig. 8E, F, G). One should favour one method over the other as a function of its goal: if the goal is to predict the abundance of rare cell types and scalability is not an issue, the best tool is SVR. On the opposite, if the goal is to make fewer errors globally, InstaPrism is more efficient.

## Conclusions

With this benchmark, we conducted a thorough evaluation of deconvolution algorithms across multiple omic types, addressing several critical aspects of their performance, such as robustness, scalability, and precision. The proposed framework evaluated both supervised and unsupervised approaches via diverse in silico, in vitro and in vivo benchmark datasets, including an original multi-omics dataset. The analysis spanned the impact of simulation parameters including missing or extra cell types, stronger noise or noise with gene dependence, number of samples), the detection of rare cell types, and the consistency of algorithms ranking across diverse data sources. This comprehensive evaluation allowed us to tackle some of the major challenges in selecting deconvolution methods for transcriptomic and methylomic data, while paving the way for the development of new multi-omic algorithms, a significant step forward compared with existing benchmarks.

While our benchmark represents a notable advancement, our approach has certain limitations. The use of simulated data allowed us to work with large datasets with the exact ground truth, which is essential for rigorous evaluation. Although we applied common strategies for simulating technical noise, such as Negative Binomial noise for RNA data [17] and Gaussian noise for DNAm data [20, 31], we acknowledge that our simulations may not fully capture the complexity of real data. This could partially explain the discrepancies in algorithms ranking across different datasets. Additionally, the scarcity of real-world datasets with reliable ground truth, and the uncertainty surrounding their accuracy, may also contribute to these inconsistencies [6].

Our benchmark allowed us to compare our results with those from previous studies, revealing both similarities and differences. Consistent with our benchmark, [20] and [21] also observed that RLR was the best method for deconvolution of methylation data. However, our results diverged from other studies in some respects: for example, DeconRNASeq performed better in our evaluation than reported by [7] while it is the opposite for CIBERSORT (ranked among the worst in our benchmark). We compared the original CIBERSORT script [32] with its EpiDISH implementation and found substantial differences (Additional file 1: Fig. S17). For a fair evaluation, we used the original script and recommend future users to do the same. Our benchmark includes a larger variety of datasets and applies an overall ranking that merges multiple performance metrics, providing a more holistic view of algorithm performance.

The overall benchmark score allowed us to provide a single, interpretable ranking for each method, which simplifies comparison. However, this approach comes with trade-offs. Aggregating multiple metrics into a single score means that nuances from individual metrics are inevitably lost. To address this, we recommend that users refer back to the individual metrics in cases where a specific aspect of performance, e.g. speed or rare cell type detection, predominates. This flexibility allows for a more tailored selection of algorithms based on specific research needs. Additionally, our ranking method involved several subjective choices, such as selecting and categorizing metrics, along with weighting, and normalization procedure. While these choices were informed by existing benchmarks and the literature, they influence the final ranking. We chose metrics that were classically used in other benchmarks: RMSE and MAE measure the average error between the prediction and the reality, with RMSE increasing to a greater degree than MAE in case of a few large differences, and Pearson correlation measures the linear correlation. Some benchmarks also used Jensen-Shannon divergence [33], Aitchison distance [34] or sMAPE [16, 21]. Therefore, future studies could explore alternative ranking methodologies or adjust the weights to emphasize specific performance aspects depending on the analysis context. The flexibility of our benchmark ensures that as new methods and metrics emerge, it can be readily adapted to meet evolving research needs.

Our benchmark provides valuable guidance for researchers looking to select appropriate deconvolution methods based on their datasets. Datasets can be described by several characteristics: number of features, number of cell types, technology, etc (Additional file 1: Fig. S18, Additional file 2: Supplementary Methods). We investigated if some characteristics could be linked to the easiness-of-deconvolution. We explored the sensitivity of each method to the datasets and observed that unsupervised methods tend to be more sensitive to the datasets than supervised ones (Additional file 1: Fig. S19). Although our attempts to link specific dataset characteristics, such as heterogeneity (measured by the phenotypic volume, see Additional file 2: Supplementary Methods for more details [35]), to deconvolution difficulty were inconclusive (Additional file 1: Fig. S20, Additional file 2: Supplementary Methods), this remains a promising area for future research. Expanding the benchmark to include more datasets could help clarify these relationships.

Beyond its immediate application, our benchmark offers a rigorous framework that can be used to test and validate new deconvolution methods. Thanks to our strategy of giving a single rating per method we could unambiguously evaluate 20 algorithms across classes and omics. One of the key strengths of our benchmark is the inclusion of a dual-omic evaluation, which offers a unique perspective on how deconvolution methods perform across both transcriptomic and methylomic data. We have also observed that a consensus strategy did not outperform the best methods in our evaluation (Additional file 1: Fig. S21), but this remains an area where future innovations could have a significant impact. By providing a reproducible and transparent framework, our benchmark can be reused and extended as new methods are developed, contributing to the continuous improvement of deconvolution techniques. Indeed, our benchmark is well-positioned to accommodate new types of data, such as spatial transcriptomics, which represent a growing frontier in the analysis of tumour heterogeneity.

Finally, our benchmark serves two key purposes: to evaluate the performance of existing deconvolution methods across a wide range of conditions; and to provide a flexible, extensible platform for the integration of future developments in the field. This evaluation was performed on multiple datasets, including an original multi-omics dataset. By comparing algorithms across transcriptomic and methylomic data, we have laid the groundwork for the next generation of multi-omic deconvolution tools. Moreover, the ability to integrate additional datasets and algorithms ensures that this benchmark will remain a valuable resource in the future. Looking ahead, we anticipate that our framework could be extended to include spatial deconvolution methods, further broadening its applicability and relevance in the study of complex biological systems.

## Methods

### Original PaCL2 and PaMIX datasets generation

This dataset consists of 30 mixtures and 9 pure cell types, constructed to recapitulate the heterogeneity seen in real pancreatic adenocarcinoma. The in vitro mixes contained variable proportions of (i) human tumour cells (CAPAN-1 and Mia PaCa-2), (ii) cancer associated fibroblasts (mix of 2 primary cell lines), (iii) human tumour derived-endothelial cells (HMEC) and immune cells that were FACS-sorted from healthy donors (B cells, CD4^+^ cells, CD8^+^ cells, neutrophils and M2-macrophages). All patients gave their written informed consent for the use of their specimen for research. Cells were mixed and RNA/DNA were simultaneously extracted followed by RNAseq (RNA-seq poly A) and methylome (MethEPIC 850 K). For all samples, DNA/RNA were extracted using the ALLPrep tissue kit (Qiagen, Venlo, The Netherlands) following the manufacturer’s instructions.

RNA-seq libraries were prepared using the NEBNext Ultra II Directional RNA preparation kit, and paired-end 100-bp sequencing was conducted on the NovaSeq Illumina platform. Gene expression profiles were generated using Fastq files and aligned using STAR (2.7.1a) on UCSC hg38 genome. Bam files were counted using featureCounts (v2.0.0) with options -p -s 2 -T 15 -t exon -g gene_name.

Gene counts were normalized using standard DESeq2 procedure. DNA methylation was acquired according to standard Illumina protocol for Infinium Methylation EPIC BeadChip. The raw DNA methylation intensity data files (IDAT) were processed with the R packages lumi and methylumi. We performed pre-normalization filtering (removing probes containing SNP, high intensity probes and undetected probes) and normalization using colour balance adjustment and between-sample normalisation with the “quantile” method. The gene expression data and the DNA methylation data have been deposited on GEO under accession codes GSE281204 and GSE281305.

### Data simulation

We simulated expression/methylation bulk data $\widehat{D}$ and proportion matrices $\widehat{A}$, based on real reference profile matrices *T* and single-cell transcriptomic data, and a priori knowledge on the proportions classically found in real samples (Additional file 1: Table S3). $\widehat{D}$ is of size $f\times S$, *T* of size $f\times K$ and $\widehat{A}$ of size $K\times S$, with *f* the number of features, *S* the number of samples and *K* the number of cell types.

We first generated proportions $\widehat{A}$ based on a Dirichlet distribution and a priori knowledge on realistic proportions (Additional file 1: Table S3):

1 $$\begin{aligned} \widehat{A_{,s}} \sim \text {Dir}(\alpha ) \end{aligned}$$

with $\widehat{A_{,s}}$ the vector of proportions for *K* cell types in sample *s* and $\alpha = \alpha _0 \times (\alpha _1, ..., \alpha _K)$ the shape vector of the *K* cell types. For each dataset, $\alpha$ has been chosen proportional to prior knowledge on the cell types mix in real data, multiplied by a factor $\alpha _0$ controlling the dispersion around those typical proportions (Additional file 1: Table S3). The dispersion factor mimics biological noise: the higher the factor, the lower the dispersion around typical proportions.

We then did two types of simulations. First, proportions $\widehat{A}$ were convoluted with reference profiles of pure cell types *T* to obtain the expression/methylation matrix $\widehat{D}$. Finally we added a noise $\epsilon$ to model technical noise. We generated different noises. We first added a Negative Binomial noise for RNA and a Gaussian noise on M-values for DNAm data (see Additional file 2: Supplementary Methods for the parameters of the Negative Binomial and Gaussian distributions) [17, 20, 31]. For PaCL2, we also created a noise based on Copula such that it takes into account gene-gene correlations. Shortly, we computed the empirical copula from the scaled and centered residuals $D_{\text {PaMIX}} - T_{\text {PaMIX}}\times A_{\text {PaMIX}}$ using the R package Copula. We generated a Negative Binomial (for RNA) and Gaussian (for DNAm) noise with parameters derived from the copula (see Additional file 2: Supplementary Methods).

2 $$\begin{aligned} \widehat{D} = T \times \widehat{A} + \epsilon \end{aligned}$$

Second, we did a pseudo-bulk. We used a single-cell dataset of pancreatic cells [27] and we only kept cells labelled as “Endothelial”, “Fibro/Stellate”, “Mono.Macro”, “Basal” and “Classic” based on published markers [36–47] (see Additional file 2: Supplementary Methods for details on how cells were labelled). For each sample *s*, we randomly selected 100 single cells from a unique patient [48], with the same proportions of the different cell types as in $\widehat{A_{,s}}$. This transcriptomic dataset PaPB was deconvoluted with the same reference as the PaCL2 dataset (GSE281204), restricted to the 7 common cell types: endothelial cells, fibroblasts, macrophages, basal and classical cancer types, B and T cells (with the CD4^+^ and CD8^+^ T cell profiles averaged into a single T cell profile) and the 16982 common genes.

Table 2 indicates which references were used for a given dataset. We generated sets of 10 replicates for each reference.

Removal or addition of a cell type in the reference profile matrix was performed by modifying *T*, for the experiments of missing or extra cell types. To have a reference matrix with a missing cell type, we removed the first column of $T \in \mathbb {R}^{f\times K}$ after ordering the columns alphabetically. For the extra cell type, we generated a fake cell type based on the real ones. Basically, we selected all immune cell types, computed the mean profile and added noise (see Additional file 2: Supplementary Methods). For the BrCL1 dataset, we also modified *T* by adding the monocyte cell type taken from the reference of BrCL2 across common genes and normalising the library sizes.

### Measure of cell type similarity

We quantified how different a cell type was from the other types by computing the average of the Pearson correlation coefficients between the reference profile of the cell of interest and all other types. We also did a hierarchical clustering on the reference profile matrices (restricted to the 30000 most variable CpGs for methylation references), using the Euclidean distance and the function *hclust* with default parameters.

### Computational deconvolution: formulation of the problem

The reference, proportions and expression/methylation matrices can be connected via the following equation:

3 $$\begin{aligned} \underset{f \times S}{D}\ = \underset{f \times K}{T}\ \times \underset{K \times S}{A} \end{aligned}$$

We can add further constraints of non-negativity (NN) and sum-to-one (STO) on *A*:

4 $$\begin{aligned} \left. \begin{array}{cc} A_{k,s} \in [0, 1] \text\ {(NN) }\\ \sum _{k=1}^K A_{k,s} = 1 \text\ {(STO) }\\ \end{array}\right\} \quad {\forall k \in [\![ 1,K ]\!], s\in [\![ 1,S ]\!]} \end{aligned}$$

### Supervised deconvolution algorithms

We used multiple approaches for supervised deconvolution. Several methods are based on least squares (LS): Robust Linear Regression (RLR) [49], Non-Negative LS (NNLS) [50], epidishCP [49], Ordinary LS (OLS) [50], WISP [51], DeconRNASeq [52], Elastic net and FARDEEP [53]. We added an STO constraint on FARDEEP for the method FARDEEP_sto. Other methods are based on a Support Vector Machine: CIBERSORT [32] and Support Vector Regression (SVR) [50]. The last method relies on a Bayesian approach to directly sample the columns $\tilde{A_{,s}}$ from the posterior distribution $p(A_{,s} | D_{,s}, T)$: InstaPrism [54]. InstaPrism requires to define variable cell types, which we chose to be the tumour types. Hence, InstaPrism was not run on non-cancer datasets.

For in silico data, we used the same reference matrix as the one employed for the simulations. For in vitro data, we used as reference the profiles of the pure cell types that were mixed. Except for BlMIX, the pure types were profiled at the same time as the mixes. For in vivo data, BlREAL1 and BlREAL2 were published along with matching references. We used the reference profiles from BlREAL2 to do supervised deconvolution of BlREAL3. For SkREAL, we used signatures accessed with the function *EPIC::TRef* (Table 2, column “Reference”) (package v1.1.5).

### Unsupervised deconvolution algorithms

Unsupervised deconvolution was conducted using two primary approaches. In the first approach, there is no assumptions about the reference matrix, and both *A* and *T* are estimated simultaneously through Independent Component Analysis (ICA). In the second approach, *A* and *T* are estimated alternatively using LS methods, including Non-negative Matrix Factorization (NMF), RefFreeEWAS [55], MeDeCom [56], EDec [57], and PREDE [58]. Other approaches included convex analysis with debCAM [59] or Latent Dirichlet Allocation (LDA) with CDSeq [60]. Although debCAM was initially designed for RNA data, we also tested its performance on DNAm data. For all unsupervised methods, we used the true number of cell types as the number of unsupervised components in the algorithms.

After estimating $\tilde{A}$, we identified the components by matching them to the cell types in the ground truth matrix *A*. Specifically, we aligned rows from $\tilde{A}$ to those from the ground truth *A* by solving a linear sum assignment problem, using the R package clue to order rows in a way that maximizes correlations.

### Implementation of deconvolution methods: feature selection and execution of deconvolution algorithms

Feature selection was performed using the R package TOAST (v1.16.0). The most variable features were identified with the function *findRefinx* and TOAST discriminating features with the function *csDeconv*, using ICA for the “FUN” argument. To select outlier genes, we took the 100 genes with the highest median expression across the data and we picked 10, 50, 100 genes from this pool to have 1000 HVG supplemented with $1\%,5\%,10\%$ of outlier genes. For the randomly selected genes, we picked from the pool of genes not selected as HVG.

Most algorithms were executed with default parameters (see Additional file 2: Supplementary Methods for more details). The deconvolution analyses were run with a Snakemake workflow within an Apptainer container on the University of Grenoble computing infrastructure GRICAD (https://gricad.univ-grenoble-alpes.fr). Each analysis was run on a single CPU node with 32 cores allocated for parallelized methods. The seed was fixed for reproducibility. To accelerate computation, deconvolution for methylation data was limited to the 30,000 most variable features as captured by the function *TOAST::findRefinx*.

### Performance metrics

We calculated several metrics grouped in three categories, namely:

- Raw performance: RMSE, MAE, Pearson correlation *P* on the whole predicted matrix $\tilde{A}$, median across cell types of the Pearson correlations on cell types $P_k$, median across samples of the Pearson correlations on samples $P_s$;
- Stability across replicates: standard deviation of RMSE, MAE and *P*, median across replicates of the standard deviation of $P_k$ and $P_s$;
- Time: Per-sample time of deconvolution, log-transformed, and its standard deviation across replicates.

Secondary stability metrics are calculated exclusively on replicates. The ranking process that includes both primary and secondary metrics is referred to as ‘A’, while the process based solely on primary metrics is referred to as ‘B’ (Additional file 1: Fig. S2F). When replicates were available, primary metrics were averaged by taking the median across replicates (see Additional file 2: Supplementary Methods).

We normalized the metrics as described in [61]. Let $M^X$ be the method $\times$ metric matrix for a dataset *X*. Each method is a combination of a feature selection strategy and a deconvolution algorithm. We first normalized each metric for the dataset *X* for all methods such that the different scores are all between 0 and 1, and transformed it such that 1 is the best score and 0 the worst (see Additional file 2: Supplementary Methods). After normalization-transformation, Pearson correlation metrics (resp. standard deviation of Pearson correlations) are merged into a Pearson meta-score (resp. Pearson stability meta-score) with the arithmetic mean (refer to Additional file 2: Supplementary Methods). The output of this normalization - transformation - merging step is $\bar{M}^X$.

### Ranking processes

We designed a consensus ranking processes to aggregate the different metrics into an overall score. The global aggregation process takes the method $\times$ metric matrices for all datasets as input, and outputs a vector of methods’ overall scores *S* via a three-steps process. First, metrics are aggregated using the geometric mean for each method, dataset and category. Second, we compute a weighted geometric mean to aggregate across categories for a given method and dataset, with weights of 1 for the raw performance and 0.5 for the scalability and stability. Finally, we aggregate across datasets via the arithmetic mean for each method (see Additional file 2: Supplementary Methods).

We tested three different inputs for the global aggregation described above (Additional file 1: Fig. S1). (1) For the process of successive aggregations $S_{raw}$, the input is $\bar{M}^X$. (2) For the process of average ranks $S_{rank}$, the input is the matrix of ranks $R^X$ computed separately for each metric from $\bar{M}^X$ (Additional file 1: Fig. S1, Additional file 2: Supplementary Methods). (3) For the topsis process $S_{topsis}$, the input is the matrix of TOPSIS scores computed from $\bar{M}^X$. The TOPSIS score $T_i^X$ measures a ratio of the distances between each method *i* and archetypes of the best and worst possible methods for each dataset *X* (Additional file 1: Fig. S1, Additional file 2: Supplementary Methods) [24].

Finally, $S_{consensus}$ is the arithmetic mean of the overall scores computed from each previously described processes $S_{raw}$, $S_{rank}$ and $S_{topsis}$. We used the process $S_{consensus}$ in all main figures. Because $S_{consensus}$ is an average of three processes, we computed intermediate scores such as the aggregated score (per dataset) by getting the consensus scores of the intermediate scores from three processes, which is only a proxy of true consensus intermediate scores.

To test the quality of our ranking process, we quantified on $S_{raw}$, $S_{rank}$, $S_{topsis}$ and $S_{consensus}$ a series of criteria in order to characterize their behaviour [23]. We computed the average rank which is the normalized average rank of the winner, the Condorcet rate which is the rate of ranking the Condorcet winner first when one exists, and the generalization criterion which quantifies of how much a ranking depends on the set of metrics used (Additional file 2: Supplementary Methods).

### Statistical significance of the overall score

We used a permutation test in order to associate p-values to the overall scores. For each pair of methods *i*, *j*, with *i* having a higher overall score than *j*, we did a one-sided test measuring how significant the difference in score was. We randomly shuffled their scores $M_{i,}$ and $M_{j,}$ 1,000 times. For each iteration *k*, we computed the overall scores $S_i^k$ and $S_j^k$ using the process $S_{consensus}$, and the associated test statistic $s_k = S_i^k - S_j^k$. Finally, we compared the observed test statistic $s_0 = S_i - S_j$ to the distribution $\{s_k, k\in [\![ 1,1000 ]\!] \}$.

## Supplementary information

Additional file 1. Supplementary figures S1-S21 and supplementary tables S1-S3 referenced in the main text.

Additional file 2. Supplementary methods.

## Data Availability

The code for the deconvolution pipeline along with the Apptainer container definition file, and the code to reproduce the figures for the ranking pipeline are available on GitHub (https://github.com/bcm-uga/DeconvBenchmark). The deconvolution pipeline can be adapted to add new methods and datasets. Similarly, the ranking pipeline can be modified. The references profiles and scRNAseq dataset used for the generation of the in silico mixes are available on Zenodo (10.5281/zenodo.14024478). The references and accession codes of the public datasets used in this study are listed in Table 2 and the directions on where to retrieve the proportion matrices for the in vitro mixes are given on our GitHub. The datasets we generated, PaMIX and PaCL2, have been deposited on GEO under accession codes GSE281204 and GSE281305 [62] (gene expression and DNA methylation data). All other datasets except PaCL1 and PaPB were downloaded from the GEO database with the following accession codes: GSE123604 and GSE87297 for BrCL1 [63, 64], GSE64655 for BlCL [65], GSE220605 for BrCL2 and BrMIX [66], GSE92843 and GSE88824 for LuCL [67, 68], GSE110554 for BlMIX [69], GSE60424 for BlREAL1 [70], GSE35069 for BlREAL2 and the reference profiles of BlREAL3 [71], GSE42861 for BlREAL3 [72] and GSE93722 for SkREAL [73]. PaCL1 was downloaded from the GTEx Portal and from [41]. Single-cell data used for PaPB was downloaded from [27].

## References

[1] Shen-Orr SS, Gaujoux R. Computational deconvolution: extracting cell type-specific information from heterogeneous samples. Curr Opin Immunol. 2013;25(5):571–8. 10.1016/j.coi.2013.09.015. 24148234 10.1016/j.coi.2013.09.015 PMC3874291

[2] Finotello F, Trajanoski Z. Quantifying tumor-infiltrating immune cells from transcriptomics data. Cancer Immunol Immunother. 2018;67(7):1031–40. 10.1007/s00262-018-2150-z. 29541787 10.1007/s00262-018-2150-z PMC6006237

[3] De Visser KE, Joyce JA. The evolving tumor microenvironment: from cancer initiation to metastatic outgrowth. Cancer Cell. 2023;41(3):374–403. 10.1016/j.ccell.2023.02.016. 36917948 10.1016/j.ccell.2023.02.016

[4] Schwartz R, Shackney SE. Applying unmixing to gene expression data for tumor phylogeny inference. BMC Bioinformatics. 2010;11(1):42. 10.1186/1471-2105-11-42. 20089185 10.1186/1471-2105-11-42 PMC2823708

[5] Zheng S. Benchmarking: contexts and details matter. Genome Biol. 2017;18(1):129. 10.1186/s13059-017-1258-3. 28679434 10.1186/s13059-017-1258-3 PMC5499005

[6] Garmire LX, Li Y, Huang Q, Xu C, Teichmann SA, Kaminski N, et al. Challenges and perspectives in computational deconvolution of genomics data. Nat Methods. 2024;21(3):391–400. 10.1038/s41592-023-02166-6. 38374264 10.1038/s41592-023-02166-6

[7] Avila Cobos F, Alquicira-Hernandez J, Powell JE, Mestdagh P, De Preter K. Benchmarking of cell type deconvolution pipelines for transcriptomics data. Nat Commun. 2020;11(1):5650. 10.1038/s41467-020-19015-1. 33159064 10.1038/s41467-020-19015-1 PMC7648640

[8] Nadel BB, Oliva M, Shou BL, Mitchell K, Ma F, Montoya DJ, et al. Systematic evaluation of transcriptomics-based deconvolution methods and references using thousands of clinical samples. Brief Bioinform. 2021;22(6):bbab265. 10.1093/bib/bbab265. 34346485 10.1093/bib/bbab265 PMC8768458

[9] Vallania F, Tam A, Lofgren S, Schaffert S, Azad TD, Bongen E, et al. Leveraging heterogeneity across multiple datasets increases cell-mixture deconvolution accuracy and reduces biological and technical biases. Nat Commun. 2018;9(1):4735. 10.1038/s41467-018-07242-6. 30413720 10.1038/s41467-018-07242-6 PMC6226523

[10] He D, Chen M, Wang W, Song C, Qin Y. Deconvolution of tumor composition using partially available DNA methylation data. BMC Bioinformatics. 2022;23(1):355. 10.1186/s12859-022-04893-7. 36002797 10.1186/s12859-022-04893-7 PMC9400327

[11] Houseman EA, Kile ML, Christiani DC, Ince TA, Kelsey KT, Marsit CJ. Reference-free deconvolution of DNA methylation data and mediation by cell composition effects. BMC Bioinformatics. 2016;17(1):259. 10.1186/s12859-016-1140-4. 27358049 10.1186/s12859-016-1140-4 PMC4928286

[12] Yamawaki TM, Lu DR, Ellwanger DC, Bhatt D, Manzanillo P, Arias V, et al. Systematic comparison of high-throughput single-cell RNA-seq methods for immune cell profiling. BMC Genomics. 2021;22(1):66. 10.1186/s12864-020-07358-4. 33472597 10.1186/s12864-020-07358-4 PMC7818754

[13] Hippen AA, Omran DK, Weber LM, Jung E, Drapkin R, Doherty JA, et al. Performance of computational algorithms to deconvolve heterogeneous bulk ovarian tumor tissue depends on experimental factors. Genome Biol. 2023;24(1):239. 10.1186/s13059-023-03077-7. 37864274 10.1186/s13059-023-03077-7 PMC10588129

[14] Avila Cobos FA, Panah MJN, Epps J, Long X, Man TK, Chiu HS, et al. Effective methods for bulk RNA-seq deconvolution using scnRNA-seq transcriptomes. Genome Biol. 2023;24(1):177. 10.1186/s13059-023-03016-6. 37528411 10.1186/s13059-023-03016-6 PMC10394903

[15] Dietrich A, Merotto L, Pelz K, Eder B, Zackl C, Reinisch K, et al. Benchmarking second-generation methods for cell-type deconvolution of transcriptomic data; 2024. Accessed September 2024. Preprint at https://www.biorxiv.org/content/10.1101/2024.06.10.598226v1

[16] Zhang W, Zhang X, Liu Q, Wei L, Qiao X, Gao R, et al. Deconer: A comprehensive and systematic evaluation toolkit for reference-based cell type deconvolution algorithms using gene expression data; 2023. Accessed July 2024. Preprint at https://www.biorxiv.org/content/10.1101/2023.12.24.573278v1

[17] Jin H, Liu Z. A benchmark for RNA-seq deconvolution analysis under dynamic testing environments. Genome Biol. 2021;22(1):102. 10.1186/s13059-021-02290-6. 33845875 10.1186/s13059-021-02290-6 PMC8042713

[18] Sturm G, Finotello F, Petitprez F, Zhang JD, Baumbach J, Fridman WH, et al. Comprehensive evaluation of transcriptome-based cell-type quantification methods for immuno-oncology. Bioinformatics. 2019;35(14):i436–45. 10.1093/bioinformatics/btz363. 31510660 10.1093/bioinformatics/btz363 PMC6612828

[19] Decamps C, Privé F, Bacher R, Jost D, Waguet A, HADACA consortium, et al. Guidelines for cell-type heterogeneity quantification based on a comparative analysis of reference-free DNA methylation deconvolution software. BMC Bioinformatics. 2020;21(1):16. 10.1186/s12859-019-3307-2. 31931698 10.1186/s12859-019-3307-2 PMC6958785

[20] Teschendorff AE, Breeze CE, Zheng SC, Beck S. A comparison of reference-based algorithms for correcting cell-type heterogeneity in Epigenome-Wide Association Studies. BMC Bioinformatics. 2017;18(1):105. 10.1186/s12859-017-1511-5. 28193155 10.1186/s12859-017-1511-5 PMC5307731

[21] Song J, Kuan PF. A systematic assessment of cell type deconvolution algorithms for DNA methylation data. Brief Bioinform. 2022;23(6):bbac449. 10.1093/bib/bbac449. 36242584 10.1093/bib/bbac449 PMC9947552

[22] Mölder F, Jablonski KP, Letcher B, Hall MB, Tomkins-Tinch CH, Sochat V, et al. Sustainable data analysis with Snakemake. F1000Res. 2021. 10.12688/f1000research.29032.2. 34035898 10.12688/f1000research.29032.1 PMC8114187

[23] Pavao A, Vaccaro M, Guyon I. Judging competitions and benchmarks: a candidate election approach; 2021. Paper presented at ESANN 2021. 10.14428/esann/2021.ES2021-122.

[24] Hwang CL, Yoon K. Methods for Multiple Attribute Decision Making. In: Multiple Attribute Decision Making. Lecture Notes in Economics and Mathematical Systems. vol. 186. Berlin, Heidelberg: Springer Berlin Heidelberg; 1981. pp. 58–191. 10.1007/978-3-642-48318-9_3.

[25] Li Z, Wu H. Toast: improving reference-free cell composition estimation by cross-cell type differential analysis. Genome Biol. 2019;20(1):1–17. 10.1186/s13059-019-1778-0. 31484546 10.1186/s13059-019-1778-0 PMC6727351

[26] Cai M, Yue M, Chen T, Liu J, Forno E, Lu X, et al. Robust and accurate estimation of cellular fraction from tissue omics data via ensemble deconvolution. Bioinformatics. 2022;38(11):3004–10. 10.1093/bioinformatics/btac279. 35438146 10.1093/bioinformatics/btac279 PMC9991889

[27] Peng J, Sun BF, Chen CY, Zhou JY, Chen YS, Chen H, et al. Single-cell RNA-seq highlights intra-tumoral heterogeneity and malignant progression in pancreatic ductal adenocarcinoma. Cell Res. 2019;29(9):725–38. 10.1038/s41422-019-0195-y. 31273297 10.1038/s41422-019-0195-y PMC6796938

[28] Avila Cobos F, Vandesompele J, Mestdagh P, De Preter K. Computational deconvolution of transcriptomics data from mixed cell populations. Bioinformatics. 2018;34(11):1969–79. 10.1093/bioinformatics/bty019. 29351586 10.1093/bioinformatics/bty019

[29] Sutton GJ, Poppe D, Simmons RK, Walsh K, Nawaz U, Lister R, et al. Comprehensive evaluation of deconvolution methods for human brain gene expression. Nat Commun. 2022;13(1):1358. 10.1038/s41467-022-28655-4. 35292647 10.1038/s41467-022-28655-4 PMC8924248

[30] Zaitsev K, Bambouskova M, Swain A, Artyomov MN. Complete deconvolution of cellular mixtures based on linearity of transcriptional signatures. Nat Commun. 2019;10(1):2209. 10.1038/s41467-019-09990-5. 31101809 10.1038/s41467-019-09990-5 PMC6525259

[31] Chalise P, Raghavan R, Fridley BL. InterSIM: simulation tool for multiple integrative “omic datasets.” Comput Methods Programs Biomed. 2016;128:69–74. 10.1016/j.cmpb.2016.02.011. 27040832 10.1016/j.cmpb.2016.02.011 PMC4833453

[32] Newman AM, Liu CL, Green MR, Gentles AJ, Feng W, Xu Y, et al. Robust enumeration of cell subsets from tissue expression profiles. Nat Methods. 2015;12(5):453–7. 10.1038/nmeth.3337. 25822800 10.1038/nmeth.3337 PMC4739640

[33] Sang-aram C, Browaeys R, Seurinck R, Saeys Y. Spotless: a reproducible pipeline for benchmarking cell type deconvolution in spatial transcriptomics. eLife. 2023;12. 10.7554/eLife.88431.1. 10.7554/eLife.88431 PMC11126312 38787371

[34] Tran KA, Addala V, Johnston RL, Lovell D, Bradley A, Koufariotis LT, et al. Performance of tumour microenvironment deconvolution methods in breast cancer using single-cell simulated bulk mixtures. Nat Commun. 2023;14(1):5758. 10.1038/s41467-023-41385-5. 37717006 10.1038/s41467-023-41385-5 PMC10505141

[35] Azizi E, Carr AJ, Plitas G, Cornish AE, Konopacki C, Prabhakaran S, et al. Single-cell map of diverse immune phenotypes in the breast tumor microenvironment. Cell. 2018;174(5):1293-1308.e36. 10.1016/j.cell.2018.05.060. 29961579 10.1016/j.cell.2018.05.060 PMC6348010

[36] Maurer C, Holmstrom SR, He J, Laise P, Su T, Ahmed A, et al. Experimental microdissection enables functional harmonisation of pancreatic cancer subtypes. Gut. 2019;68(6):1034–43. 10.1136/gutjnl-2018-317706. 30658994 10.1136/gutjnl-2018-317706 PMC6509007

[37] Baron M, Veres A, Wolock SL, Faust AL, Gaujoux R, Vetere A, et al. A Single-Cell Transcriptomic Map of the Human and Mouse Pancreas Reveals Inter- and Intra-cell Population Structure. Cell Syst. 2016;3(4):346-360.e4. 10.1016/j.cels.2016.08.011. 27667365 10.1016/j.cels.2016.08.011 PMC5228327

[38] Chen EY, Tan CM, Kou Y, Duan Q, Wang Z, Meirelles GV, et al. Enrichr: interactive and collaborative HTML5 gene list enrichment analysis tool. BMC Bioinformatics. 2013;14:128. 10.1186/1471-2105-14-128. 23586463 10.1186/1471-2105-14-128 PMC3637064

[39] Enge M, Arda HE, Mignardi M, Beausang J, Bottino R, Kim SK, et al. Single-cell analysis of human pancreas reveals transcriptional signatures of aging and somatic mutation patterns. Cell. 2017;171(2):321-330.e14. 10.1016/j.cell.2017.09.004. 28965763 10.1016/j.cell.2017.09.004 PMC6047899

[40] Bailey P, Chang DK, Nones K, Johns AL, Patch AM, Gingras MC, et al. Genomic analyses identify molecular subtypes of pancreatic cancer. Nature. 2016;531(7592):47–52. 10.1038/nature16965. 26909576 10.1038/nature16965

[41] Nicolle R, Blum Y, Marisa L, Loncle C, Gayet O, Moutardier V, et al. Pancreatic adenocarcinoma therapeutic targets revealed by tumor-stroma cross-talk analyses in patient-derived xenografts. Cell Rep. 2017;21(9):2458–70. 10.1016/j.celrep.2017.11.003. 29186684 10.1016/j.celrep.2017.11.003 PMC6082139

[42] Collisson EA, Sadanandam A, Olson P, Gibb WJ, Truitt M, Gu S, et al. Subtypes of pancreatic ductal adenocarcinoma and their differing responses to therapy. Nat Med. 2011;17(4):500–3. 10.1038/nm.2344. 21460848 10.1038/nm.2344 PMC3755490

[43] Moffitt RA, Marayati R, Flate EL, Volmar KE, Loeza SGH, Hoadley KA, et al. Virtual microdissection identifies distinct tumor- and stroma-specific subtypes of pancreatic ductal adenocarcinoma. Nat Genet. 2015;47(10):1168–78. 10.1038/ng.3398. 26343385 10.1038/ng.3398 PMC4912058

[44] Puleo F, Nicolle R, Blum Y, Cros J, Marisa L, Demetter P, et al. Stratification of pancreatic ductal adenocarcinomas based on tumor and microenvironment features. Gastroenterology. 2018;155(6):1999-2013.e3. 10.1053/j.gastro.2018.08.033. 30165049 10.1053/j.gastro.2018.08.033

[45] Elyada E, Bolisetty M, Laise P, Flynn WF, Courtois ET, Burkhart RA, et al. Cross-species single-cell analysis of pancreatic ductal adenocarcinoma reveals antigen-presenting cancer-associated fibroblasts. Cancer Discov. 2019;9(8):1102–23. 10.1158/2159-8290.CD-19-0094. 31197017 10.1158/2159-8290.CD-19-0094 PMC6727976

[46] Becht E, Giraldo NA, Lacroix L, Buttard B, Elarouci N, Petitprez F, et al. Estimating the population abundance of tissue-infiltrating immune and stromal cell populations using gene expression. Genome Biol. 2016;17(1):218. 10.1186/s13059-016-1070-5. 27908289 10.1186/s13059-016-1113-y PMC5134277

[47] Bindea G, Mlecnik B, Tosolini M, Kirilovsky A, Waldner M, Obenauf AC, et al. Spatiotemporal dynamics of intratumoral immune cells reveal the immune landscape in human cancer. Immunity. 2013;39(4):782–95. 10.1016/j.immuni.2013.10.003. 24138885 10.1016/j.immuni.2013.10.003

[48] Hu M, Chikina M. Heterogeneous pseudobulk simulation enables realistic benchmarking of cell-type deconvolution methods. Genome Biol. 2024;25(1):169. 10.1186/s13059-024-03292-w. 38956606 10.1186/s13059-024-03292-w PMC11218230

[49] Teschendorff AE, Zheng SC. EpiDISH. Bioconductor. 2017. 10.18129/B9.bioc.EpiDISH.

[50] Pfister S, Kuettel V, Ferrero E. granulator: Rapid benchmarking of methods for in silico deconvolution of bulk RNA-seq data. 2022. 10.18129/B9.bioc.granulator.

[51] Blum Y, Meiller C, Quetel L, Elarouci N, Ayadi M, Tashtanbaeva D, et al. Dissecting heterogeneity in malignant pleural mesothelioma through histo-molecular gradients for clinical applications. Nat Commun. 2019;10(1):1333. 10.1038/s41467-019-09307-6. 30902996 10.1038/s41467-019-09307-6 PMC6430832

[52] Gong T, Szustakowski JD. DeconRNASeq: a statistical framework for deconvolution of heterogeneous tissue samples based on mRNA-Seq data. Bioinformatics. 2013;29(8):1083–5. 10.1093/bioinformatics/btt090. 23428642 10.1093/bioinformatics/btt090

[53] Hao Y, Yan M, Heath BR, Lei YL, Xie Y. Fast and robust deconvolution of tumor infiltrating lymphocyte from expression profiles using least trimmed squares. PLoS Comput Biol. 2019;15(5):e1006976. 10.1371/journal.pcbi.1006976. 31059559 10.1371/journal.pcbi.1006976 PMC6522071

[54] Hu M, Chikina M. InstaPrism: an R package for fast implementation of BayesPrism. 2023. Accessed August 2024. Preprint at https://www.biorxiv.org/content/10.1101/2023.03.07.531579v2 10.1093/bioinformatics/btae440 PMC11245312 38970377

[55] Houseman EA, Molitor J, Marsit CJ. Reference-free cell mixture adjustments in analysis of DNA methylation data. Bioinformatics. 2014;30(10):1431–9. 10.1093/bioinformatics/btu029. 24451622 10.1093/bioinformatics/btu029 PMC4016702

[56] Lutsik P, Slawski M, Gasparoni G, Vedeneev N, Hein M, Walter J. MeDeCom: discovery and quantification of latent components of heterogeneous methylomes. Genome Biol. 2017;18(1):55. 10.1186/s13059-017-1182-6. 28340624 10.1186/s13059-017-1182-6 PMC5366155

[57] Onuchic V, Hartmaier RJ, Boone DN, Samuels ML, Patel RY, White WM, et al. Epigenomic deconvolution of breast tumors reveals metabolic coupling between constituent cell types. Cell Rep. 2016;17(8):2075–86. 10.1016/j.celrep.2016.10.057. 27851969 10.1016/j.celrep.2016.10.057 PMC5115176

[58] Qin Y, Zhang W, Sun X, Nan S, Wei N, Wu HJ, et al. Deconvolution of heterogeneous tumor samples using partial reference signals. PLoS Comput Biol. 2020;16(11):e1008452. 10.1371/journal.pcbi.1008452. 33253170 10.1371/journal.pcbi.1008452 PMC7728196

[59] Wang N, Hoffman EP, Chen L, Chen L, Zhang Z, Liu C, et al. Mathematical modelling of transcriptional heterogeneity identifies novel markers and subpopulations in complex tissues. Sci Rep. 2016;6(1):18909. 10.1038/srep18909. 26739359 10.1038/srep18909 PMC4703969

[60] Kang K, Meng Q, Shats I, Umbach DM, Li M, Li Y, et al. CDSeq: a novel complete deconvolution method for dissecting heterogeneous samples using gene expression data. PLoS Comput Biol. 2019;15(12):e1007510. 10.1371/journal.pcbi.1007510. 31790389 10.1371/journal.pcbi.1007510 PMC6907860

[61] Saelens W, Cannoodt R, Todorov H, Saeys Y. A comparison of single-cell trajectory inference methods. Nat Biotechnol. 2019;37(5):547–54. 10.1038/s41587-019-0071-9. 30936559 10.1038/s41587-019-0071-9

[62] Amblard E, Chuffart F, Kermezli Y, Armenoult L, Baurès A, Cassard A, et al. Generation of multi-omic datasets using high-throughput molecular profiling of transcriptomic human data. Gene Expr Omnibus. 2025. Accessed in 2024. https://identifiers.org/geo:GSE281204, https://identifiers.org/geo:GSE281305.

[63] Kang K, Meng Q, Shats I, Umbach D, Li M, Li Y, et al. A novel computational complete deconvolution method using RNA-seq data. Gene Expr Omnibus. 2018. Accessed in 2022. https://identifiers.org/geo:GSE123604.

[64] Onuchic V, Hartmaier R, Boone D, Samuels ML, Patel RY, White WM, et al. Epigenomic deconvolution of breast tumors reveals metabolic coupling between constituent cell types. Gene Expr Omnibus. 2016. Accessed in 2022. https://identifiers.org/geo:GSE87297. 10.1016/j.celrep.2016.10.057 PMC5115176 27851969

[65] Hoek KL, Link AJ. A Cell-based Systems Biology Assessment of Human Blood to Monitor Immune Responses After Influenza Vaccination. Gene Expr Omnibus. 2015. Accessed in 2023. https://identifiers.org/geo:GSE64655. 10.1371/journal.pone.0118528 PMC4338067 25706537

[66] Sumazin P. Effective methods for bulk RNA-seq deconvolution using scnRNA-seq transcriptomes. Gene Expr Omnibus. 2023. Accessed in 2023. https://identifiers.org/geo:GSE220605. 10.1186/s13059-023-03016-6 PMC10394903 37528411

[67] Zimmermann T, Bartkuhn M, Dammann R. Genome-wide DNA-methylation profiles of human lung cancer cell lines and normal lung cells. Gene Expr Omnibus. 2017. Accessed in 2023. https://identifiers.org/geo:GSE92843.

[68] Fox AD, Field J, Gresle MM, Johnson L, Laverick L, Butzkueven H, et al. Deconvolution of whole blood DNA methylomes reveals immune cell type-specific differential methylation in Multiple Sclerosis. Gene Expr Omnibus. 2017. Accessed in 2023. https://identifiers.org/geo:GSE88824.

[69] Salas LA, Koestler DC, Butler RA, Hansen HM, Wiencke JK, Kelsey KT, et al. An optimized library for reference-based deconvolution of whole-blood biospecimens assayed using the Illumina HumanMethylationEPIC BeadArray. Gene Expr Omnibus. 2018. Accessed in 2023. https://identifiers.org/geo:GSE110554. 10.1186/s13059-018-1448-7 PMC5975716 29843789

[70] Speake C, Linsley PS, Whalen E, Chaussabel D, Presnell SR, Mason MJ, et al. Next generation sequencing of human immune cell subsets across diseases. Gene Expr Omnibus. 2015. Accessed in 2024. https://identifiers.org/geo:GSE60424.

[71] Reinius LE, Acevedo N, Joerink M, Pershagen G, Dahlén S, Greco D, et al. Differential DNA Methylation in Purified Human Blood Cells. Gene Expr Omnibus. 2012. Accessed in 2024. https://identifiers.org/geo:GSE35069. 10.1371/journal.pone.0041361 PMC3405143 22848472

[72] Liu Y, Feinberg AP. Differential DNA methylation in Rheumatoid arthritis. Gene Expr Omnibus. 2013. Accessed in 2024. https://identifiers.org/geo:GSE42861.

[73] Racle J, de Jonge K, Baumgaertner P, Speiser DE, Gfeller D. Simultaneous enumeration of cancer and immune cell types from tumor gene expression data. Gene Expr Omnibus. 2017. Accessed in 2024. https://identifiers.org/geo:GSE93722. 10.7554/eLife.26476 PMC5718706 29130882

