# Supplementary material for: A robust workflow to benchmark deconvolution of multi-omic data
Source: Genome Biol. 2025 Dec 17;26:429. doi: 10.1186/s13059-025-03897-9 (PMC12713266; doi:10.1186/s13059-025-03897-9)
Supplement: Supplementary file 1 — Additional file 1. Supplementary figures S1-S21 and supplementary tables S1-S3 referenced in the main text. [file 13059_2025_3897_MOESM1_ESM.pdf]

A ROBUST WORKFLOW TO BENCHMARK DECONVOLUTION OF MULTI-OMIC DATA
ADDITIONAL FILE 1: SUPPLEMENTARY FIGURES

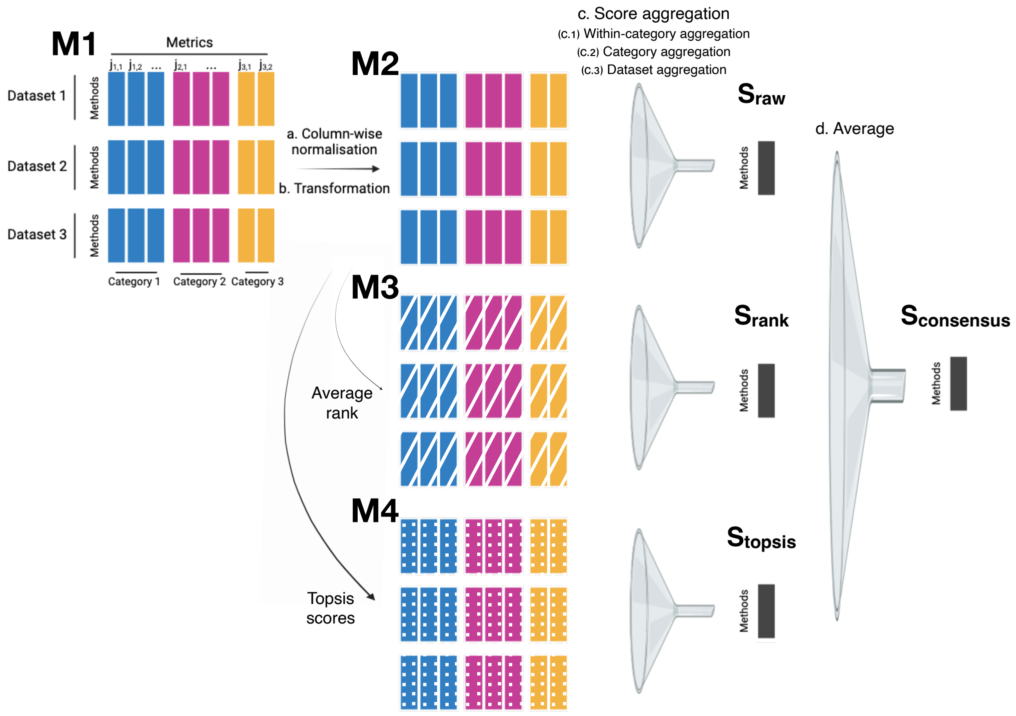

Fig. S1: Design of the different ranking processes. The first step of normalisation (a) and transformation (b) on the metric-by-dataset-by-method score matrix **M1** is common to all processes. The matrix **M2** then goes through the global 3-steps aggregation process (c) described in Fig. 1C (S_{raw}), or is used to compute average ranks **(M3)** and topsis scores **(M4)**. Matrices **M3** and **M4** then go through the global aggregation process (c) (respectively S_{rank} and S_{topsis}). The 3 ranking processes S_{raw}, S_{rank} and S_{topsis} are finally averaged (d) into $S_{consensus}$.

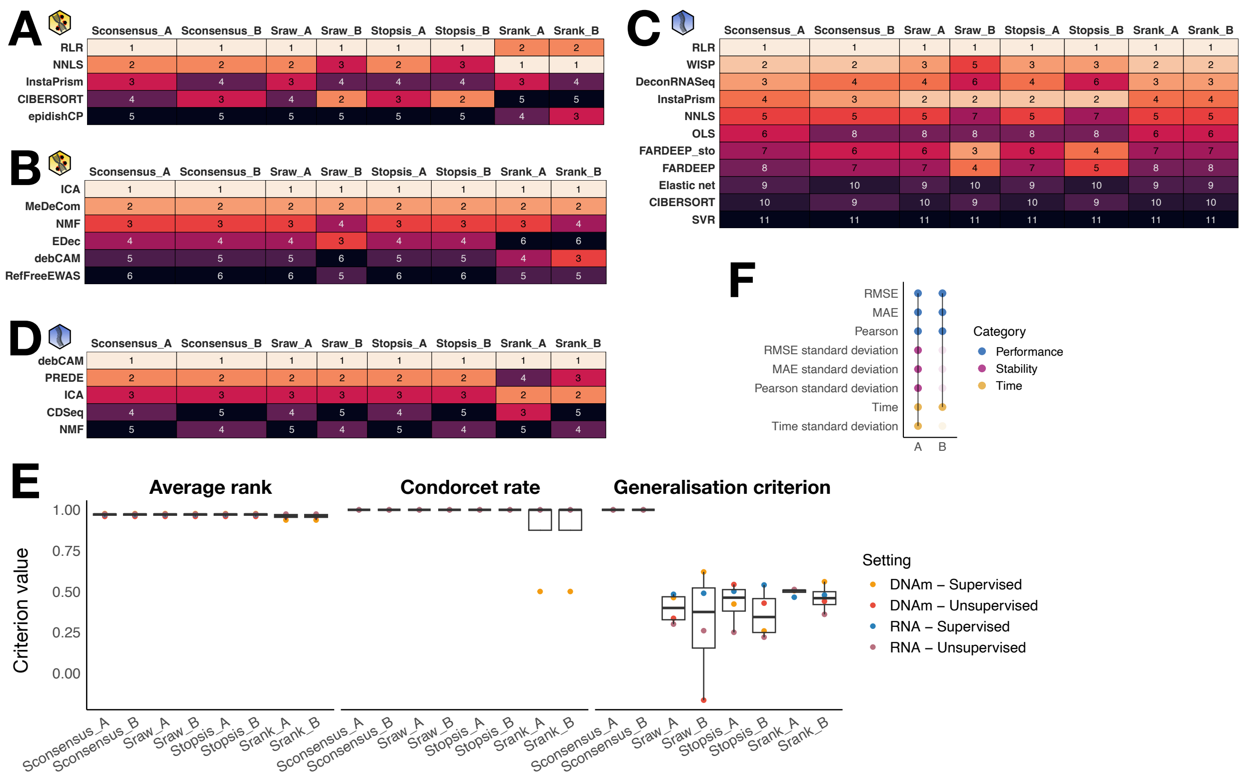

Fig. S2: Comparison of the different ranking processes. Various ranking processes yield comparable rankings, especially for the top performing candidates. Ranking tables for the 4 ranking processes tested, for the methylation (**A,B**, yellow DNA icon) and transcriptome (**C,D**, blue icon) blocks and the supervised (**A,C**) or unsupervised (**B,D**) classes of methods. **(E)** Goodness-of-ranking criteria display similar scores for the different ranking processes, except for the generalisation criterion which shows the superiority of the consensus process. **(F)** Combinations A and B include different metrics.

Method	Approach	DOI	Reviewed in	Supervised	Language	Included	Details
**ARIC**	ν-SVR	10.1093/bib/bbab362	1, 2	Yes	Python	No	
**BayesCCE**	Bayesian	10.1186/s13059-018-1513-2	2, 3	Semi	MATLAB	No	
**CelFEER**	EM	10.1093/nargab/lqad048	2	Semi	Python	No	
**CelFiE**	EM	10.1038/s41467-021-22901-x	2	Semi	Python	No	
**cfSort**	DNN	10.1073/pnas.2305236120	2	Yes	Python	No	
**CIBERSORT**	ν-SVR	10.1038/nmeth.3337	2, 4, 5, 7	Yes	R, Web-based	Yes	
**CIBERSORTx**	ν-SVR	10.1038/s41587-019-0114-2	2	Yes	Web-based	No	
**DXM**	HMM	10.1093/nar/gkab516	2, 6	No	Python	No	
**EDec**	NMF	10.1016/j.celrep.2016.10.057	2, 3	No	R	Yes	
**Emeth**	EM	10.1038/s41598-021-84864-9	2	Semi	R	No	
**epidishCP**	CLS	10.1186/1471-2105-13-86	1, 2, 3, 4, 5, 6, 7	Yes	R, Python	Yes	
**EPISCORE**	RPC-LS	10.1186/s13059-020-02126-9	2, 3	Yes	R	No	Requires scRNAseq data to impute a DNAm reference matrix
**HiBED**	HM	10.3389/fnins.2023.1198243	2	Yes	R	No	Specific to cerebral data
**HiTIMED**	HM	10.1186/s12967-022-03736-6	2	Yes	R	No	Similar to epidishCP
**ICA**	ICA	10.3390/ijms20184414	3	No	R, Python	Yes	
**MeDeCom**	NMF	0.1186/s13059-017-1182-6	2, 3, 6	No	R	Yes	
**MethAtlas**	NNLS	10.1038/s41467-018-07466-6	2	Yes	Python	No	
**MethylPurify**	EM	10.1186/s13059-014-0419-x	2, 6	No	Python	No	
**MethylResolver**	LS	10.1038/s42003-020-01146-2	1, 2, 3	Yes	R	No	Designed only for immune cells
**NMF/RefFreeCellMix**	NMF	10.1186/s12859-016-1140-4	2, 3, 4, 5, 7	No	R	Yes	
**PRISM**	EM	10.1093/bioinformatics/btz327	2, 6	No	Python	No	
**PRMeth**	NMF	10.1186/s12859-022-04893-7	2	Semi	R	No	
**RefFreeEWAS**	NMF	10.1093/bioinformatics/btu029	1, 2, 4, 5, 7	No	R	Yes	
**RLR**	RPC-LS	10.1186/s12859-017-1511-5	1, 2, 3, 4, 5, 7	Yes	R	Yes	
**Tsisal**	Geometric	10.1093/bioinformatics/btaa930	2	Both	R	No	
**UXM**	NNLS	10.1038/s41586-022-05580-6	2	Yes	Python	No	

Table S1: Literature review of methylation deconvolution methods. Methods in green are those who were included in this article. References for the reviews listed in the table are in the Supplementary Methods. SVR: Support Vector Regression. EM: Expectation–Maximization. DNN: Deep Neural Network. HMM: Hidden Markov model. NMF: Non-negative Matrix Factorization. LS: Least Squares. CLS: Constrained LS. RPC: Robust Partial Correlation. HM: Hierarchical Model. ICA: Independent Component Analysis. NNLS: Non Negative LS.

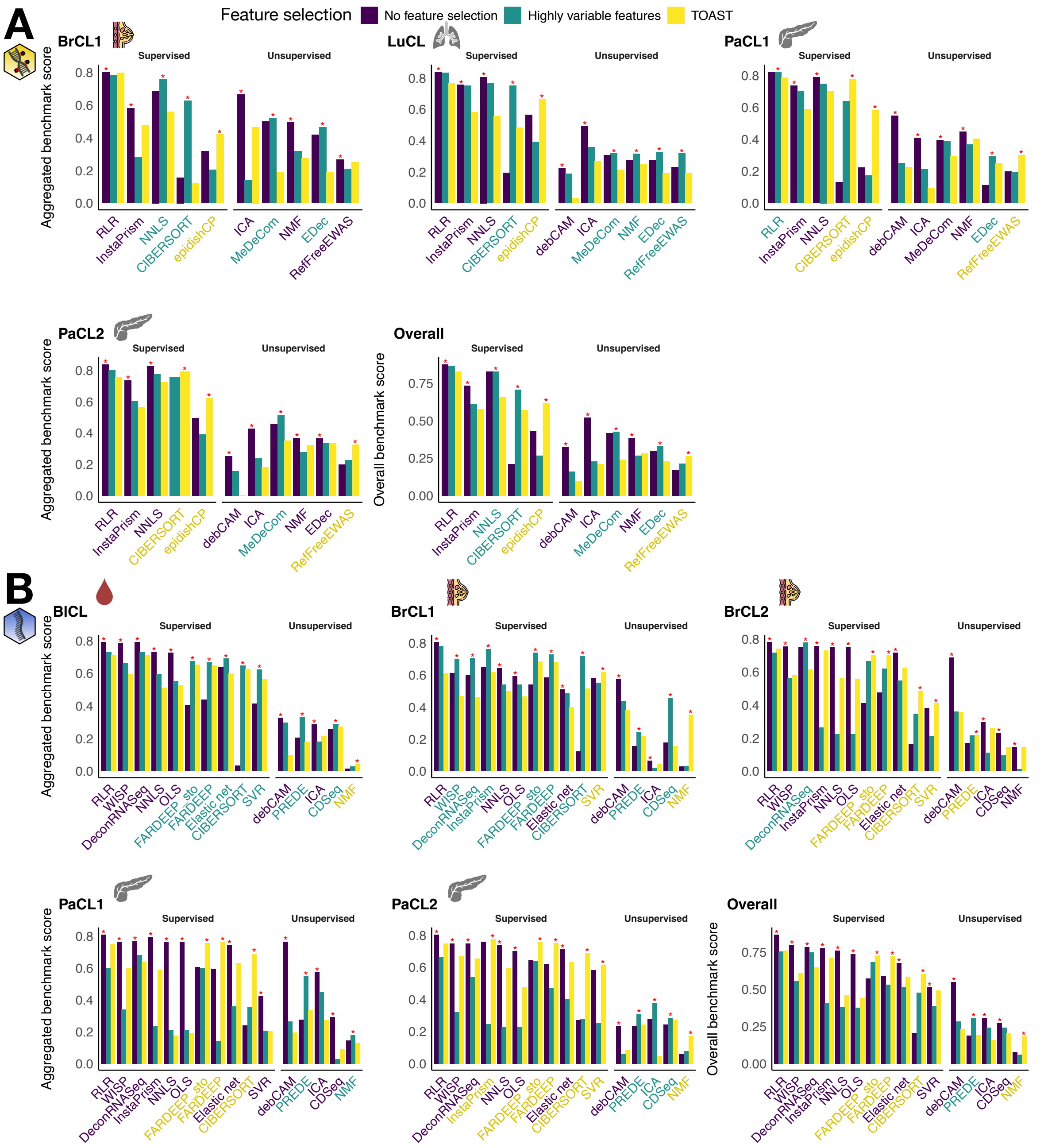

Fig. S3: Overall and aggregated scores, for each feature selection. It shows that the no feature selection strategy performs the best in most cases, both for methylation **(A)**, flagged with the yellow DNA icon, and transcriptome **(B)** data, flagged with the blue one.

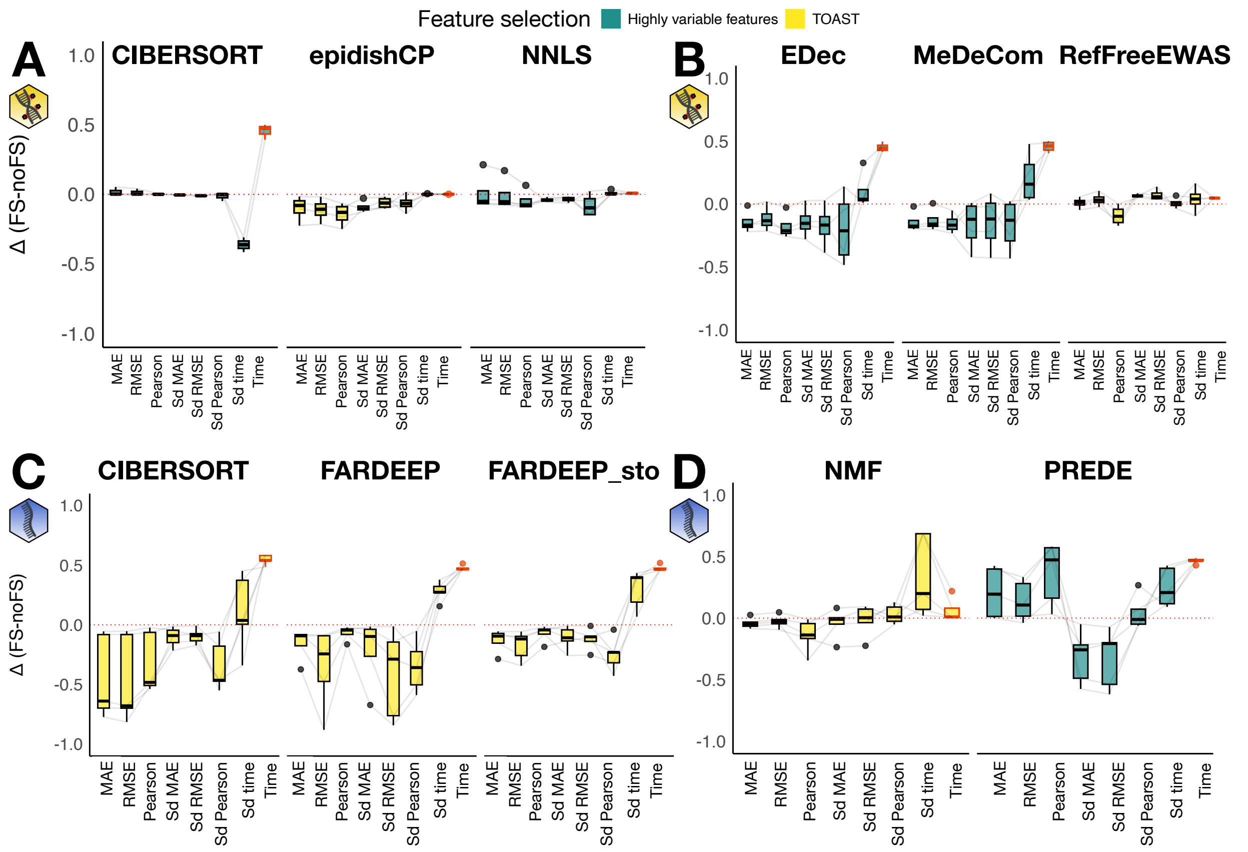

Fig. S4: Detailed performance comparison with and without feature selection. Difference in normalised-transformed atomic scores between the best feature selection and the no feature selection for candidates for which feature selection improves the overall benchmark score in simulated data (refer to Fig. **S3**). Each data point is the difference in atomic score for a given dataset. The left panels **(A,C)** show supervised methods, the right ones **(B,D)** unsupervised methods. The upper panels **(A,B)** show the results for DNAm data (yellow DNA icon), the lower ones **(C,D)** for RNA data (blue single-strand icon). The time score is in red.

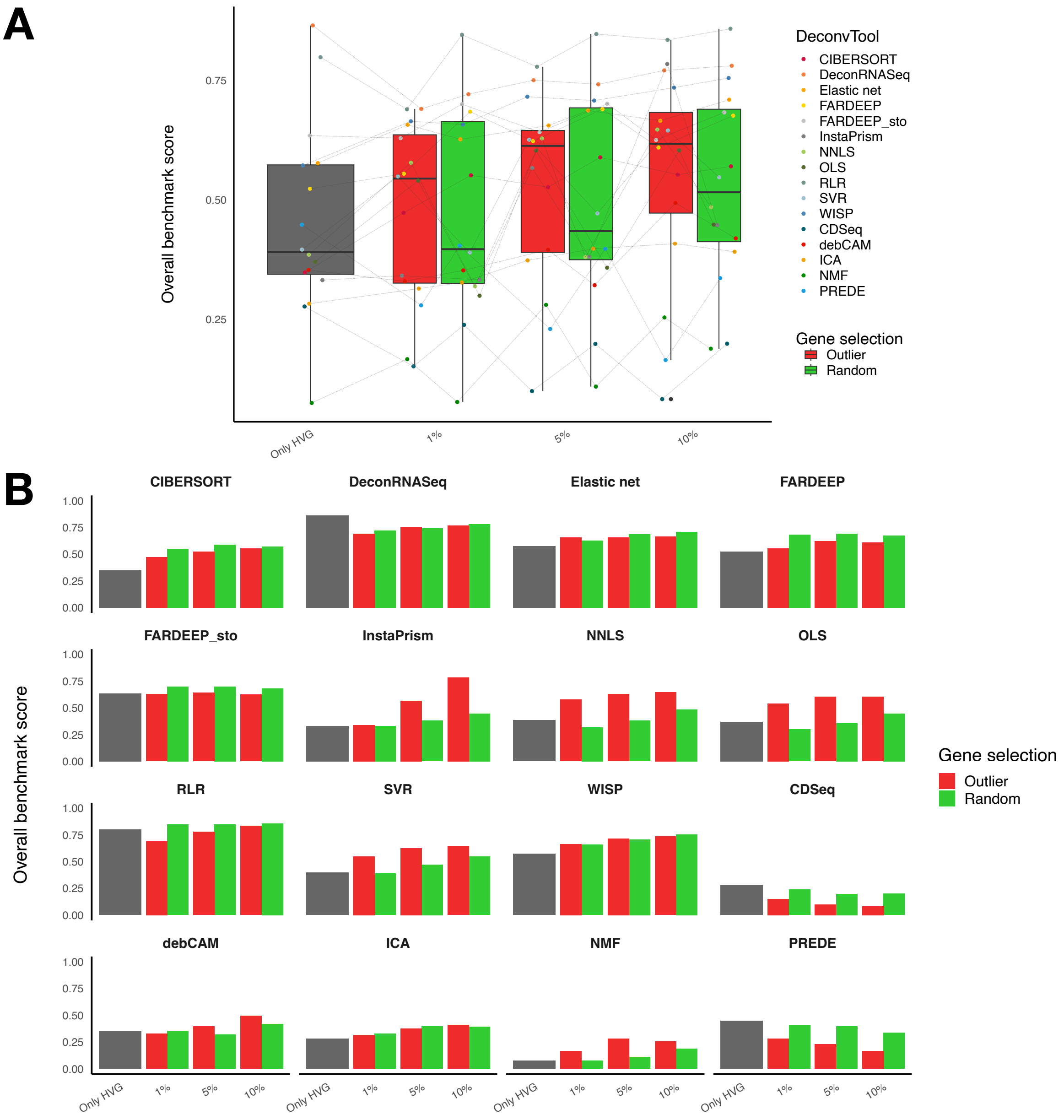

Fig. S5: Impact of the type and number of genes used for RNA deconvolution. **(A)** Boxplot of all methods. **(B)** Overall score for each RNA method.

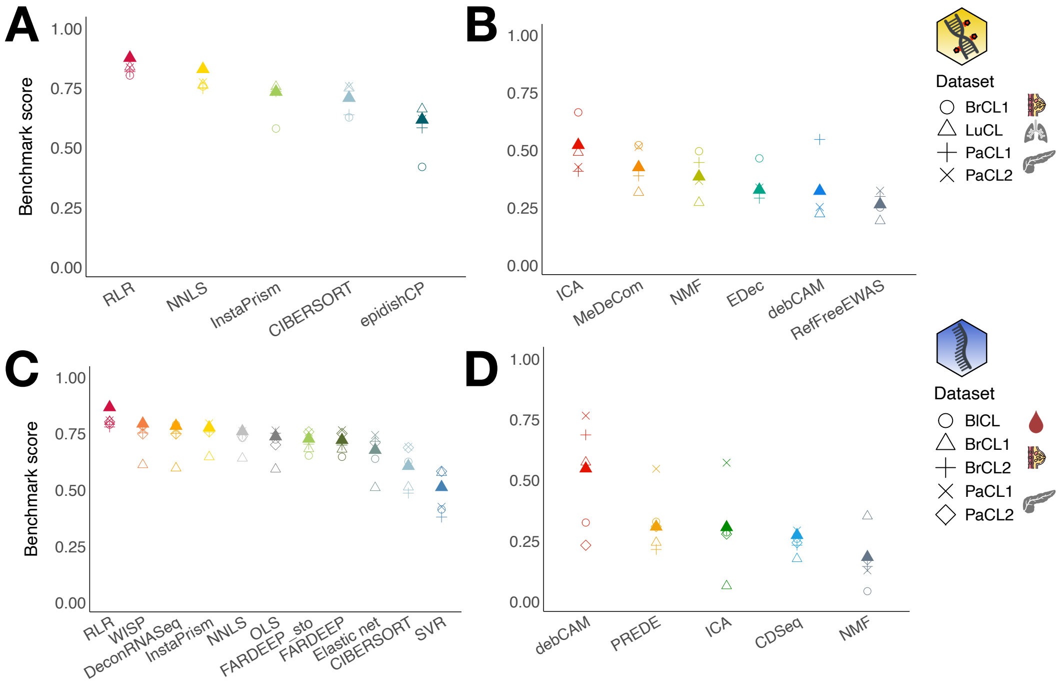

Fig. S6: Overall and aggregated benchmark scores. Scores are for the methylation **(A,B)** and transcriptome **(C,D)** blocks in the supervised **(A,C)** or unsupervised **(B,D)** classes. The overall score is depicted by the full triangle symbol.

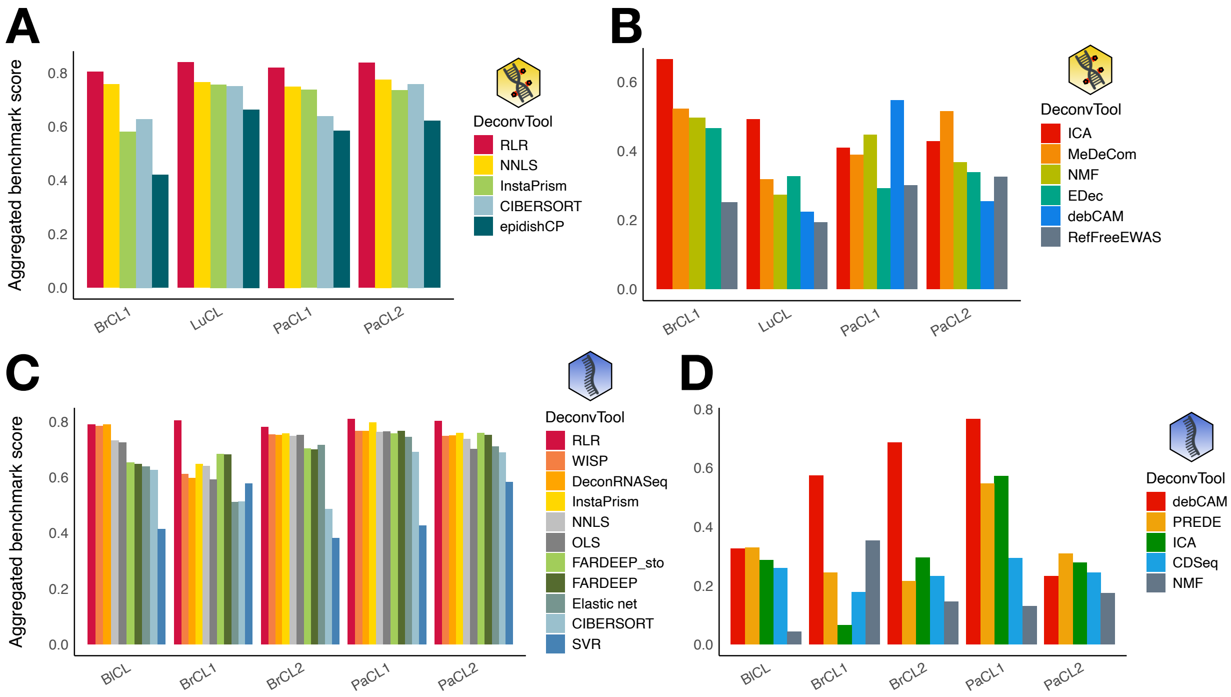

Fig. S7: Aggregated benchmark scores per dataset. They display a finer granularity in the ranking of the different candidates, as a function of the omic (methylation **(A,B)**, transcriptome **(C,D)**) and class of methods (supervised **(A,C)**, unsupervised **(B,D)**).

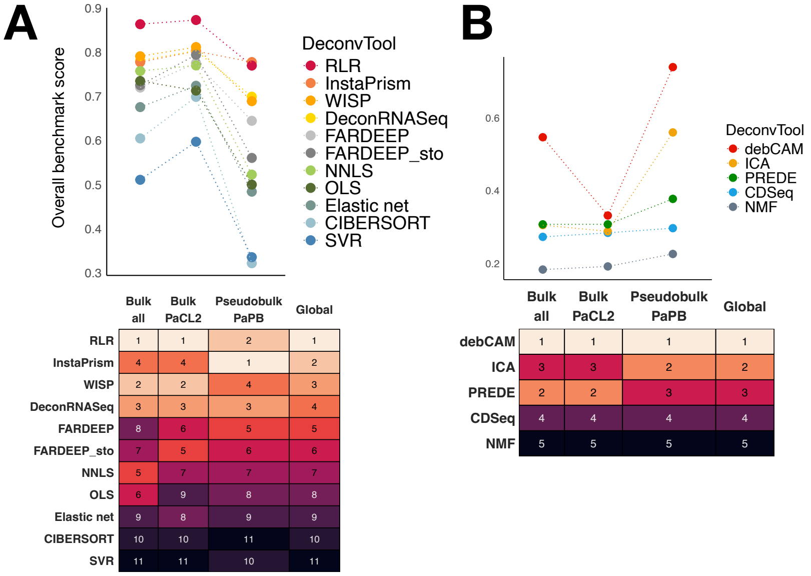

Fig. S8: Overall benchmark scores and rankings as a function of the simulating conditions: we display results for RNA bulk-based convolution simulations, including all datasets or only PaCL2, along with the pseudo-bulk dataset PaPB, in the supervised **(A)** or unsupervised **(B)** settings.

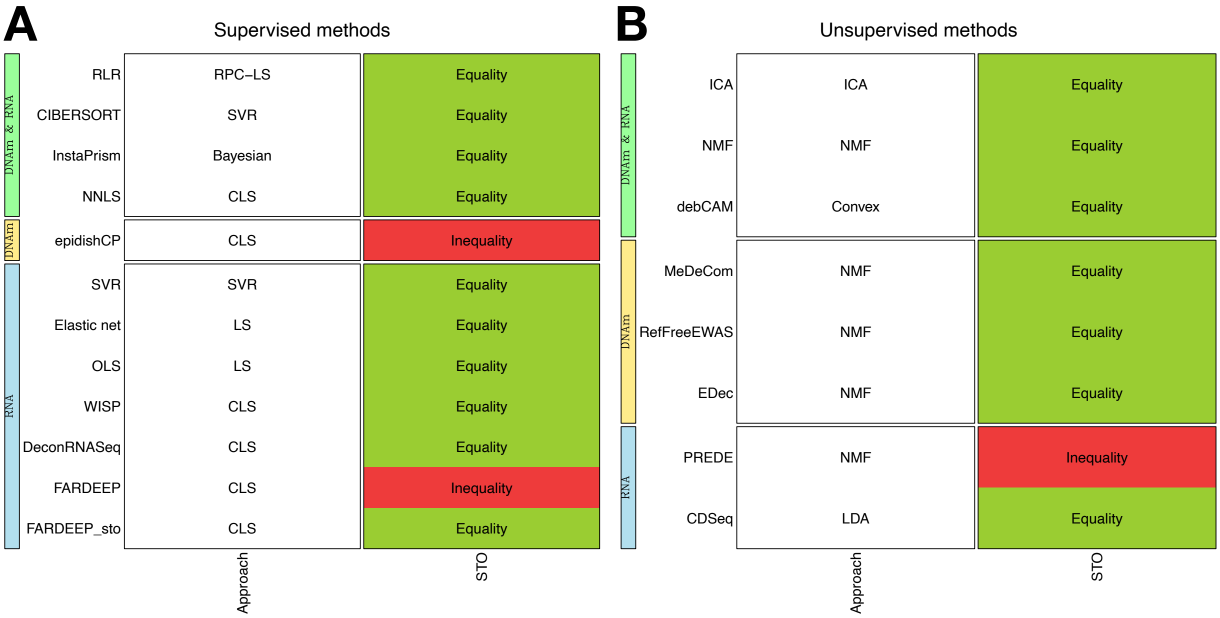

Fig. S9: Different algorithmic designs exist. Designs for the supervised **(A)** and unsupervised **(B)** classes of methods. The second column indicates whether the sum-to-one (STO) constraint is a strict equality or an inequality.

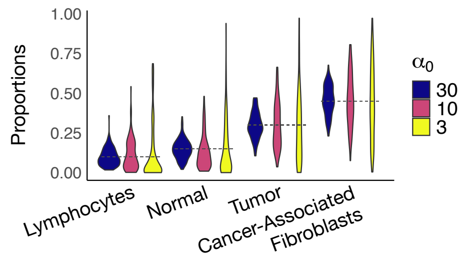

Fig. S10: The dispersion factor affects the proportions' distribution. Different values of the dispersion factor α_0 implies different ranges of variation around the proportions $\alpha_i, i \in [1, K]$ (dashed line) in the simulations. Example with the BrCL1 dataset.

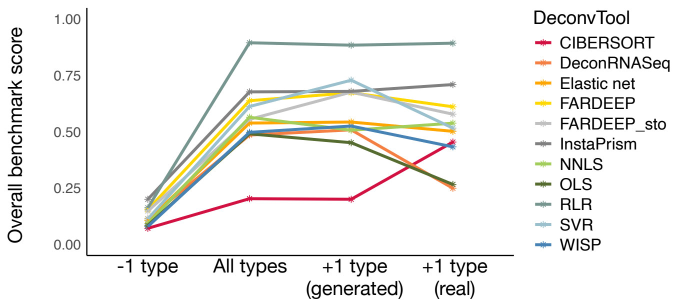

Fig. S11: Overall scores of RNA supervised methods with no feature selection applied to the BrCL1 dataset in the case of a missing, or a real or generated extra cell type in the reference matrix.

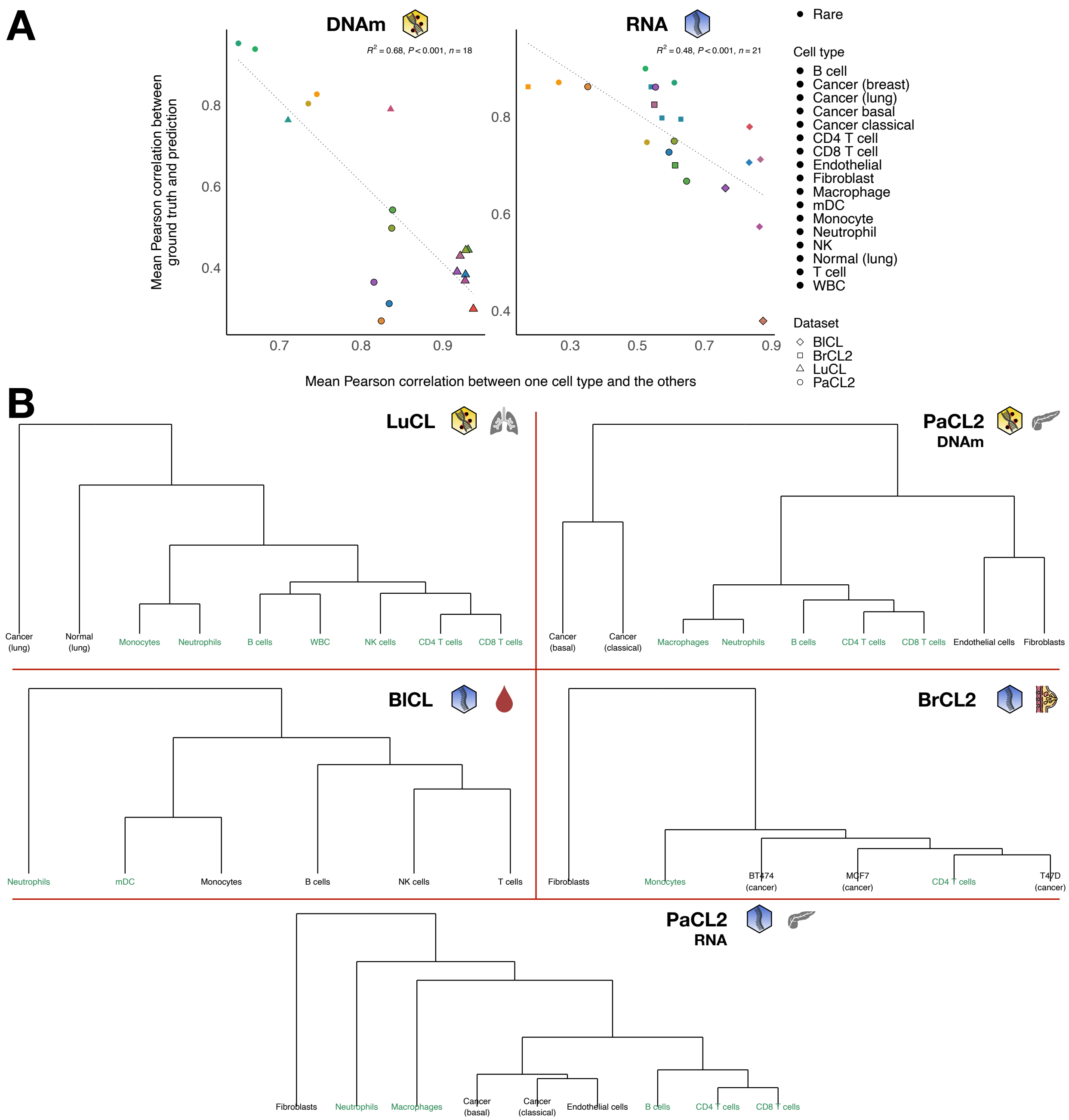

Fig. S12: Correlation across cell types. **(A)** Relationship between how correlated a cell type is to the others and the quality of its estimate. Rare types are circled in black. **(B)** Hierarchical clustering based on the Euclidean distance between cell types. Rare types are in green.

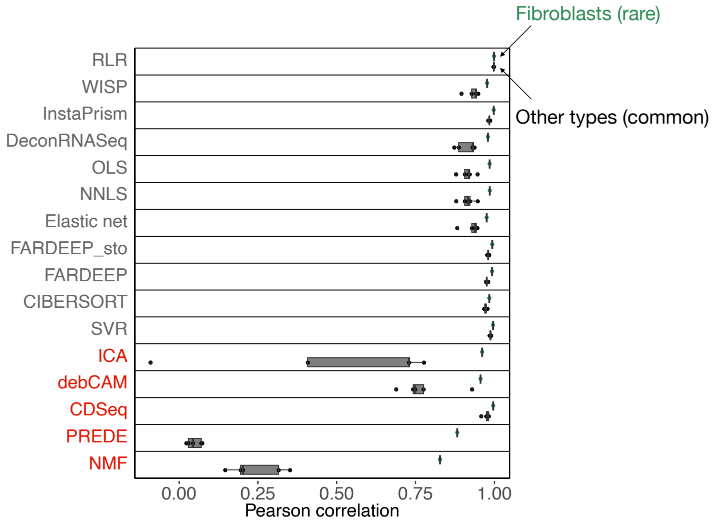

Fig. S13: Cell type Pearson correlation for fibroblasts (first line for each method) versus common types (second line) in BrCL2.

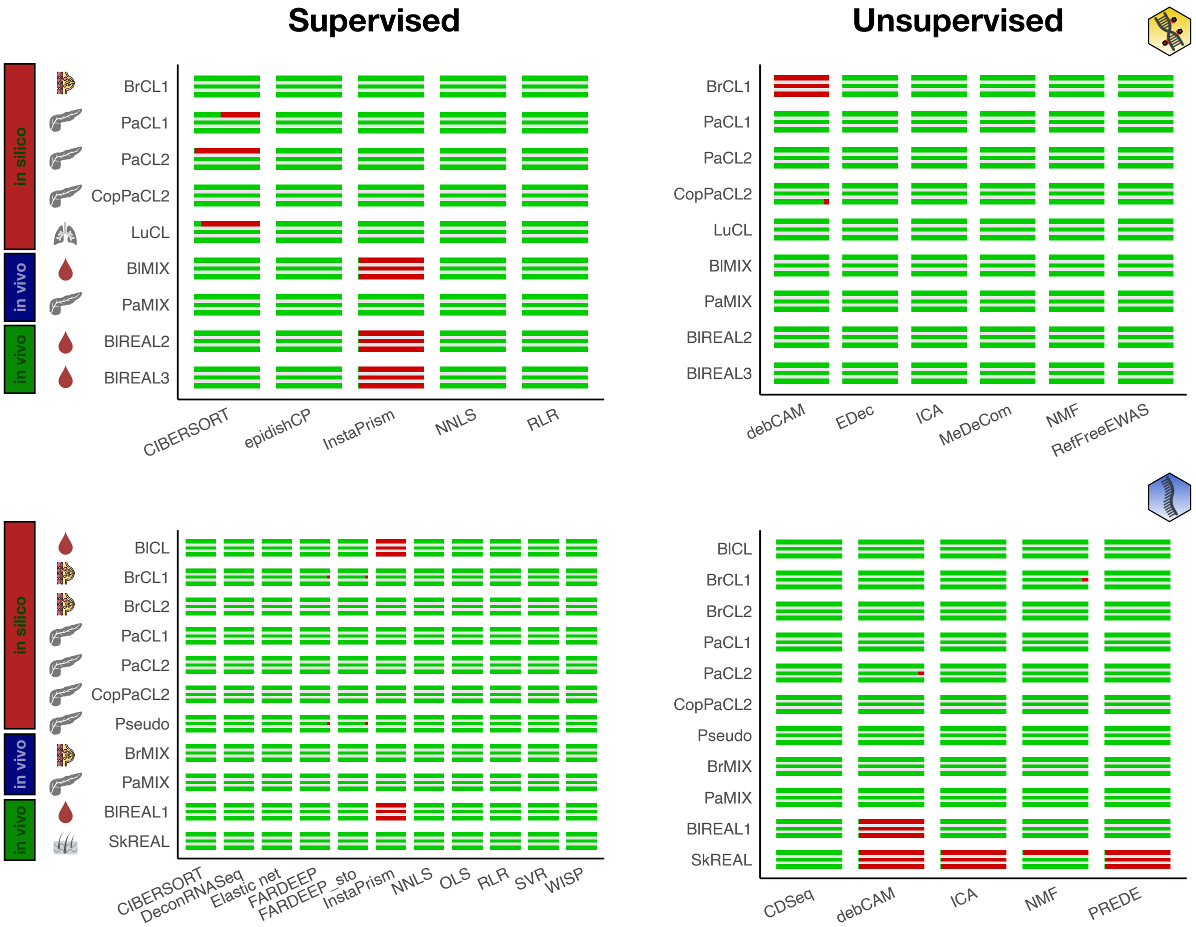

Fig. S14: Summary of successful runs (in green) on all datasets and all methods included in the benchmark. The first progress bar, for a given dataset and method stands for no feature selection, the second for highly variable features and the last for TOAST feature selection. Some methods did not run on all simulations for a given *in silico* dataset. InstaPrism runs only on cancer datasets. Failure to run for CIBERSORT happened when the method could not complete in 48 hours. The top panel is for DNAm data, the bottom one for RNA.

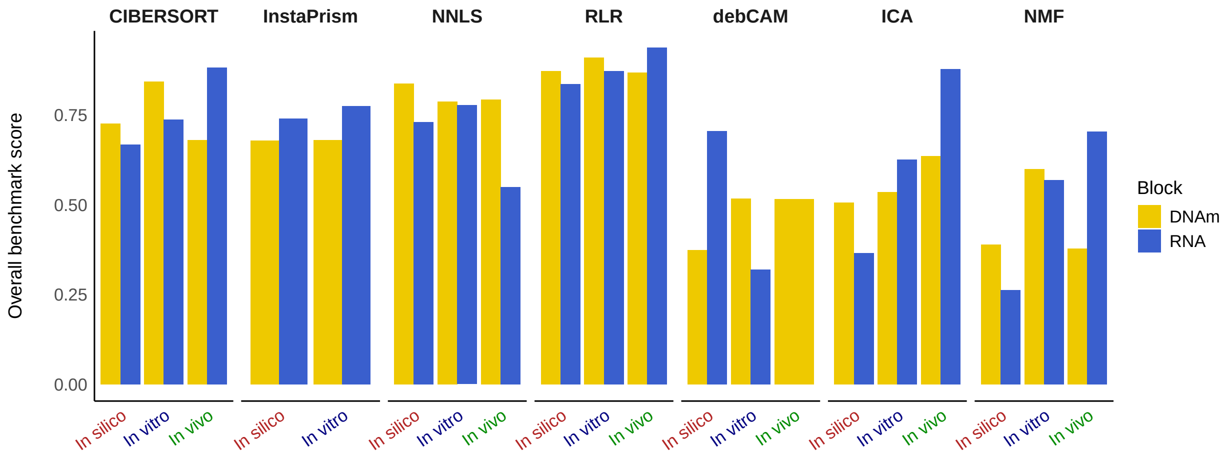

Fig. S15: Overall score for multi-omic methods and datasets across the different source: *in silico*, *in vitro* and *in vivo* data.

Method	Approach	DOI	Reviewed in	Supervised	Language	Included	Details
**Abbas**	CLS	10.1371/journal.pone.0006098	1, 2	Yes	R	No	Similar to DeconRNASeq
**AutogeneS**	ν-SVR	10.1016/j.cels.2021.05.006	3, 4	Yes	Python	No	
**BayesPrism/InstaPrism**	Bayesian	10.1038/s43018-022-00356-3	3, 4, 5	Yes	R	Yes	
**BisqueRNA**	CLS	10.1038/s41467-020-15816-6	3, 4, 5, 6	Yes	R	No	Relies on single-cell reference
**Bseq-SC**	ν-SVR	10.1016/j.cels.2016.08.011	3, 4	Yes	R	No	Relies on single-cell reference
**CDSeq**	LDA	10.1371/journal.pcbi.1007510	4	No	R	Yes	
**CIBERSORT/SVR**	ν-SVR	10.1038/nmeth.3337	2, 3, 4, 6, 7, 8, 9, 10	Yes	R	Yes	
**CIBERSORTx**	ν-SVR	10.1038/s41587-019-0114-2	4, 5, 7, 8	Yes	Web-based	No	
**CPM**	SVR	10.1038/s41592-019-0355-5	3, 4, 5	Yes	R	No	Relies on single-cell reference
**debCAM**	Convex	10.1038/srep18909	3, 4, 7	No	R	Yes	
**deconf**	NMF	10.1186/1471-2105-11-27	2, 3, 4	No	R	No	Developed for microarrays
**DeconRNASeq**	CLS	10.1093/bioinformatics/btt090	2, 3, 4, 6, 7, 9	Yes	R	Yes	
**DSection**	Bayesian	10.1093/bioinformatics/btq406	1, 11	No	MATLAB	No	
**dtangle**	LS	10.1093/bioinformatics/bty926	3, 4, 6, 9	Yes	R	No	Relies on markers
**DWLS**	CLS	10.1038/s41467-019-10802-z	3, 4, 5, 6	Yes	R	No	Relies on single-cell reference
**Elastic net**	LS	10.18637/jss.v033.i01	4, 6	Yes	R	Yes	
**EPIC**	CLS	10.7554/eLife.26476	2, 3, 4, 5, 6, 7, 8, 10	Yes	R		
**FARDEEP**	CLS	10.1371/journal.pcbi.1006976	3, 4, 6	Yes	R	Yes	
**ICA**	ICA	10.3390/ijms20184414	3	No	R	Yes	
**ImmuCellAI**	CLS	10.1002/advs.201902880	3	Yes	R		
**Lasso**	LS	10.18637/jss.v033.i01	4, 6	Yes	R		
**LinDeconSeq**	CLS	10.1186/s12864-020-06888-1	3	Yes	R	No	Relies on single-cell reference
**LinSeed**	Linear	10.1038/s41467-019-09990-5	3, 7, 9	No	R	No	Relies on markers
**MCP-Counter**	Linear	10.1186/s13059-016-1070-5	2, 3, 8, 10	Yes	R	No	Relies on markers
**MMAD**	MLE	10.1093/bioinformatics/btt566	2, 7	No	MATLAB	No	
**MOMF**	NMF	10.5281/zenodo.3373980	3, 4	Yes	R	No	Relies on single-cell reference
**MuSiC**	CLS	10.1038/s41467-018-08023-x	3, 4, 5, 6, 7, 9	Yes	R	No	Relies on single-cell reference
**NITUMID**	NMF	10.1093/bioinformatics/btz748	3	Yes	R		
**NNLS**	CLS	10.1137/1.9781611971217	4, 6	Yes	R	Yes	
**OLS**	LS	10.1007/978-3-642-50096-1_48	4, 6	Yes	R	Yes	
**PERT**	MLE	10.1371/journal.pcbi.1002838	1, 2	Yes	Octave	No	
**PREDE**	NMF	10.1371/journal.pcbi.1008452	3	Yes	R	Yes	
**proportionsInAdmixture**	LS	10.1371/journal.pone.0224693	4	Yes			
**quanTIseq**	CLS	10.1186/s13073-019-0638-6	2, 3, 8, 10	Yes	R/Docker		
**Ridge/AdRoit**	CLS	10.18637/jss.v033.i01	3, 4, 6	Yes	R	No	Relies on single-cell reference
**RLR/EpiDISH**	RPC-LS	10.1038/s41592-018-0213-x	4, 6	Yes	R	Yes	
**SCDC**	Ensemble	10.1093/bib/bbz166	3, 4, 6	Yes	R	No	Relies on single-cell reference
**ssFrobenius**	NMF	10.1186/1471-2105-11-27	2, 4, 6	Semi	R	No	
**ssKL**	NMF	10.1186/1471-2105-11-27	2, 4, 6	Semi	R	No	
**ssNMF**	NMF	10.1016/j.meegid.2011.08.014	1	Semi	R	No	
**TIMER**	CLS	10.1186/s13059-016-1028-7	2, 4, 7, 10	Yes	Web-based	No	
**WISP**	CLS	10.1038/s41467-019-09307-6		Yes	R	Yes	
**xCell**	GSEA	10.1186/s13059-017-1349-1	2, 8, 9, 10	Yes	R	No	Computes enrichment

Table S2: Literature review of transcriptome deconvolution methods. Methods in green are those who were included in this article. References for the reviews listed in the table are in the Supplementary Methods. LS: Least Squares. CLS: Constrained LS. SVR: Support Vector Regression. LDA: Latent Dirichlet Allocation. NMF: Non-negative Matrix Factorization. MLE: Maximum Likelihood Estimation. ICA: Independent Component Analysis. PCA: Principal Component Analysis. RPC: Robust Partial Correlation. GSEA: Gene Set Enrichment Analysis.

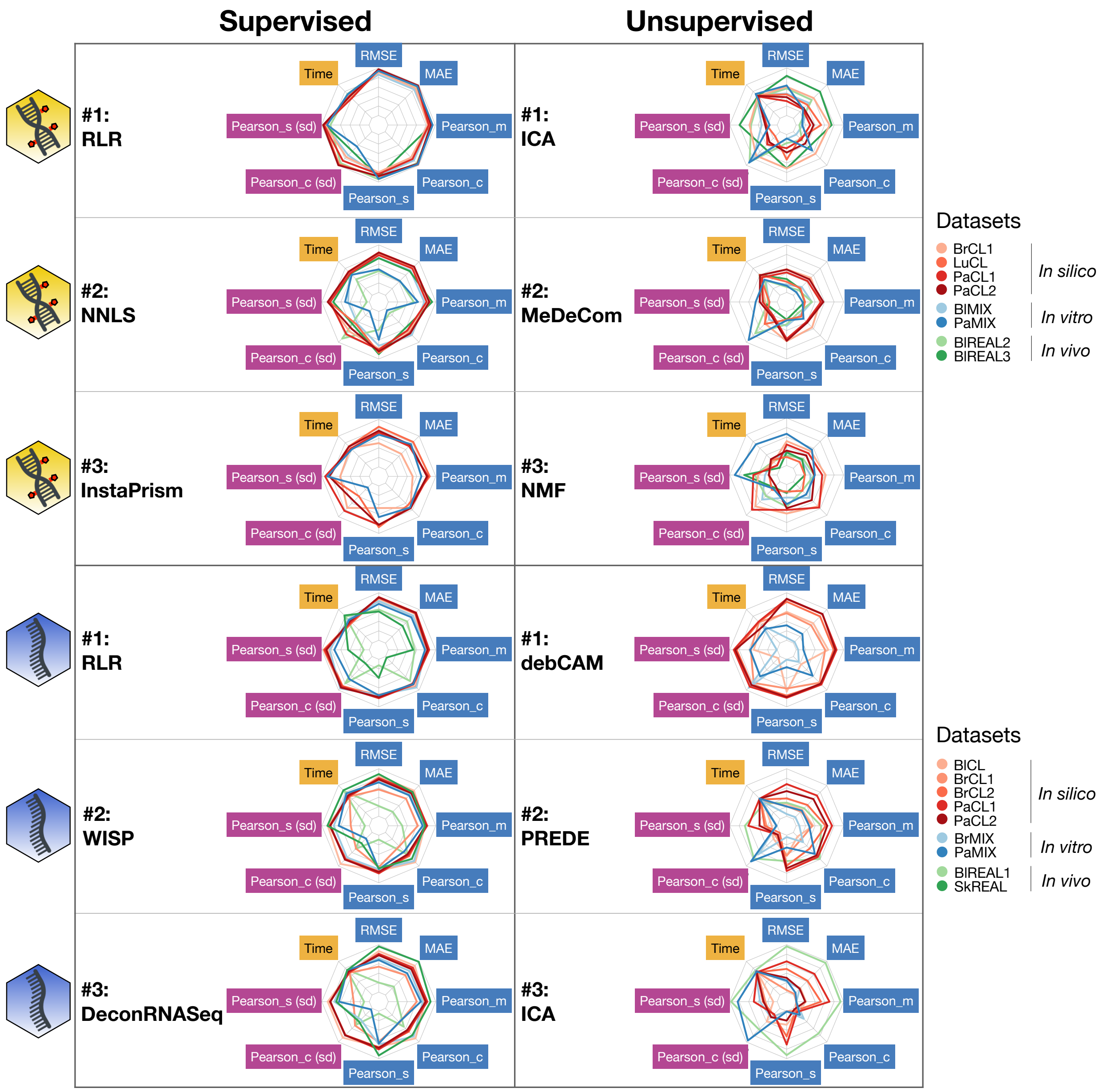

Fig. S16: Spiderplots of the top 3 best methods in each setting. Methylation is flagged with the yellow icon and transcriptome with the blue icon.

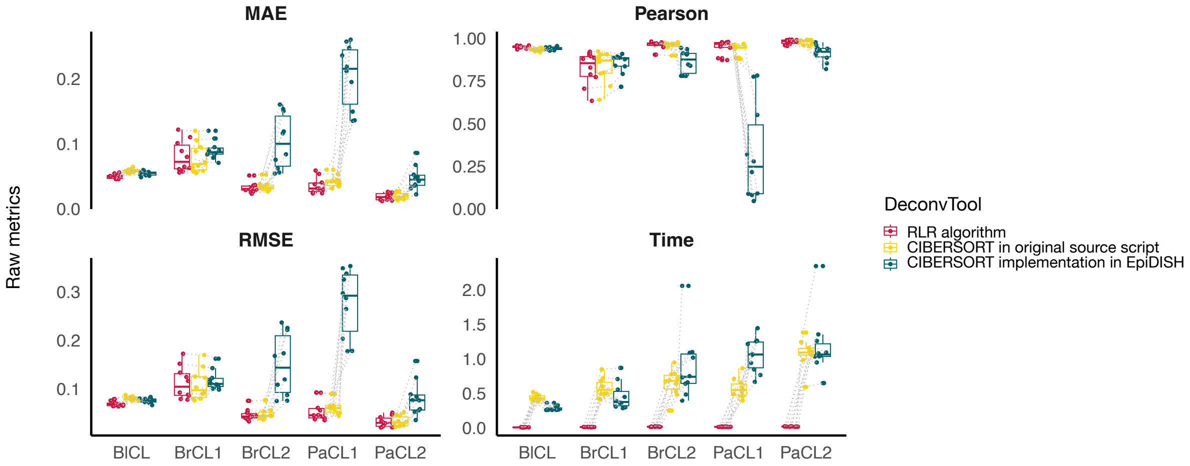

Fig. S17: Comparison of CIBERSORT implementations, between the `EpiDISH` package and the original script provided by the authors. RMSE, MAE, Pearson and time results on all bulk-based simulated RNA datasets, with the TOAST feature selection. RLR serves as an upper boundary.

Dataset	Fibroblast	T cell	B cell	NK cell	Monocyte	Neutrophil	Immune cell	Breast	Lung	Pancreas
**BrCL1**	45%	10%						45% (15% healthy, 30% cancer)		
**PaCL1**	45%						10%			45(% (15% endothelial, 30% cancer)
**PaCL2**	46%	7% (4% CD4$^+$, 3% CD8$^+$)	1%			1%	1% (Macrophage)			44% (15% endothelial, 29% cancer)
**BlCL**		60%	13%	7%	15%	4%	1% (mDC)			
**BrCL2**	45%	5% (CD4$^+$)			5%			45% (15% BT474, 15% MCF7, 15% T47D)		
**BrCL2** (rare fibroblasts)	5%	25% (CD4$^+$)			25%			45% (15% BT474, 15% MCF7, 15% T47D)		
**LuCL**		10% (5% CD4$^+$, 5% CD8$^+$)	4%	4%	4%	4%	4% (WBC)		70% (10% NHBEC, 60% A549)	

Table S3: Proportions α_i used for the different cell types in the simulated datasets. Rare cell types ($\alpha_i \leq 5\%$) are in green.

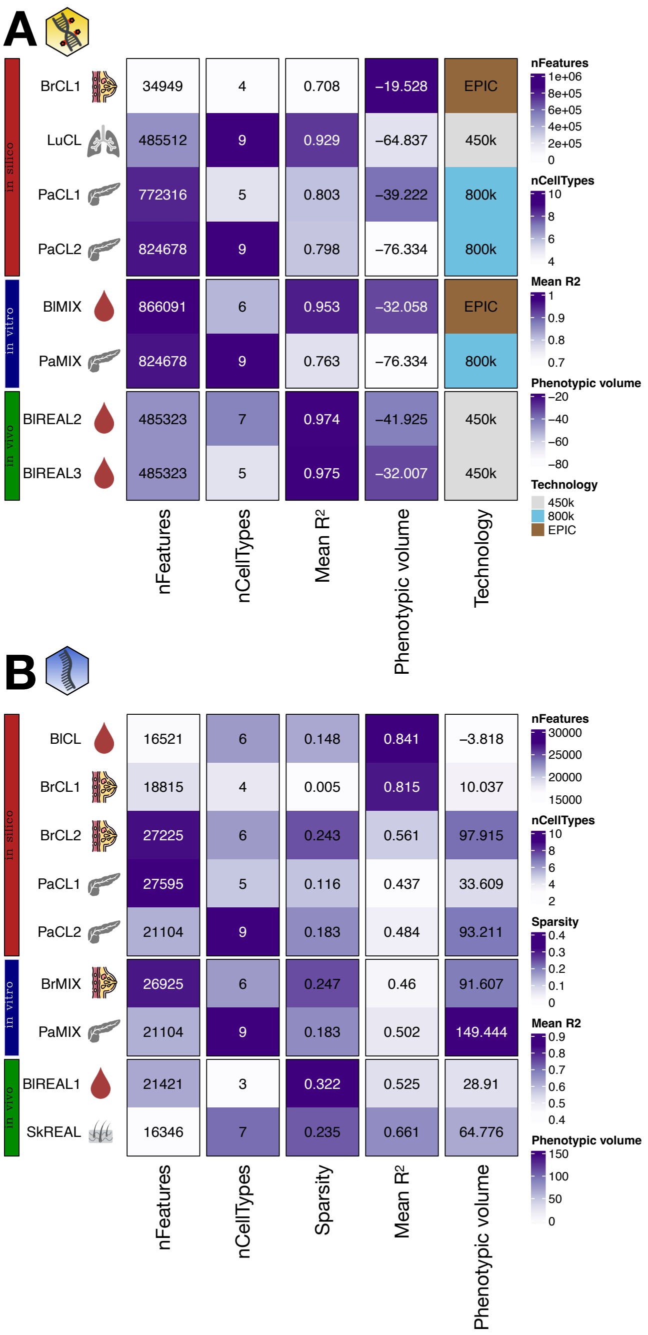

Fig. S18: Datasets characteristics. (**A**) shows DNAm datasets and **(B)** RNA datasets. We computed for both omics the number of molecular features, the number of cell types, the mean Pearson correlation between cell types profiles, and the phenotypic volume (see Additional file 2: Supplementary Methods for its definition), the two latter serving as proxies of dataset heterogeneity. We also took into account the technology for the methylation block, while all transcriptomes were sequenced with the Illumina platform. For the transcriptome block, we added the sparsity, defined as the mean of null counts.

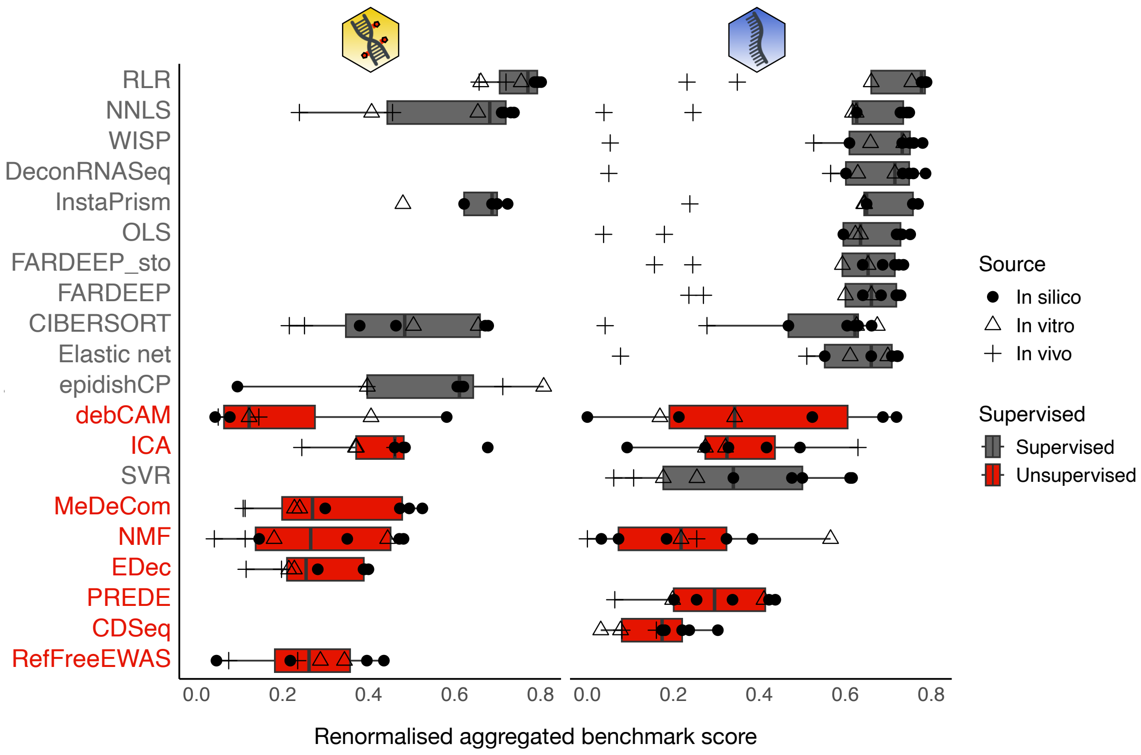

Fig. S19: Renormalized aggregated benchmark scores. Candidates are ordered from top to bottom by their overall benchmark score. The yellow DNA icon represents DNAm methods, the blue single-strand one RNA methods.

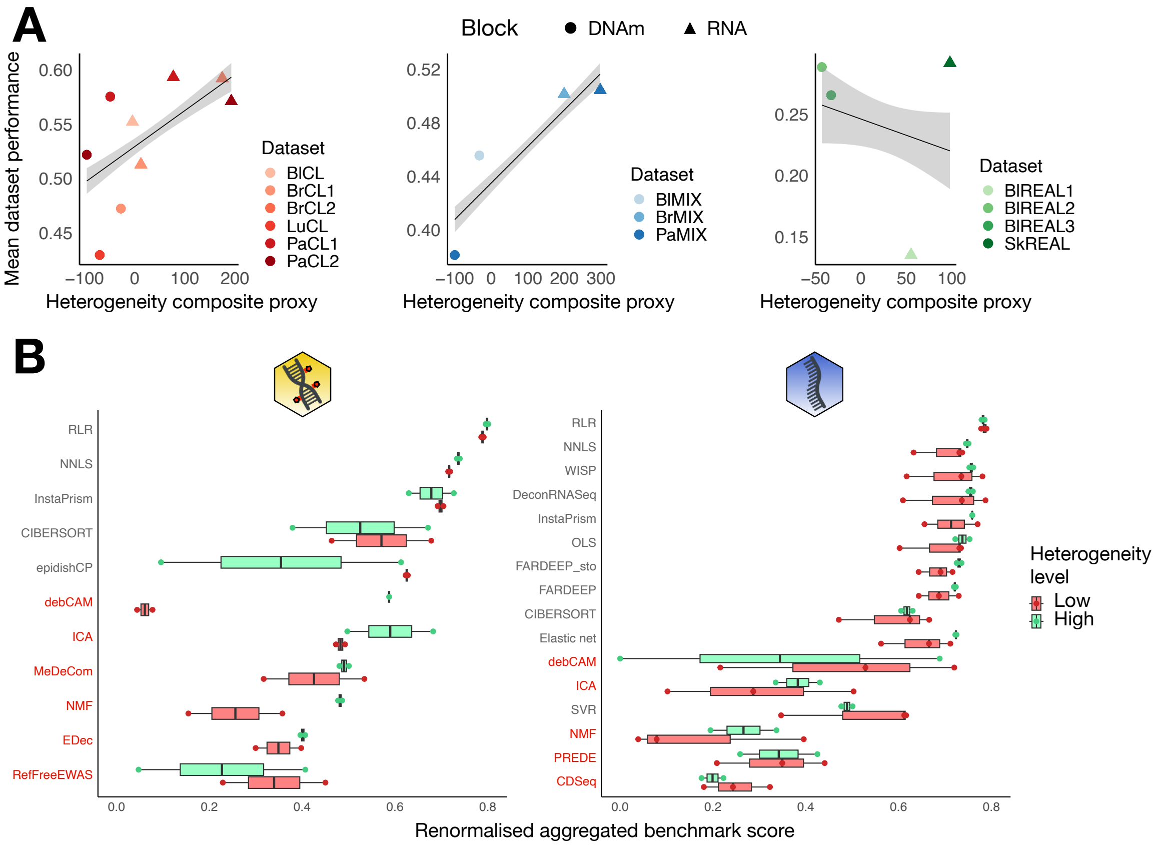

Fig. S20: Impact of heterogeneity levels on the general deconvolution performance. **(A)** Global deconvolution performance is weakly impacted by dataset heterogeneity. Each facet represents a data source: *in silico*, *in vitro* and *in vivo*. **(B)** Effect of the heterogeneity level of *in silico* datasets on each method's performance as a function of the omic type: the yellow icon stands for DNAm methods, the blue one for RNA methods. Unsupervised methods are in red.

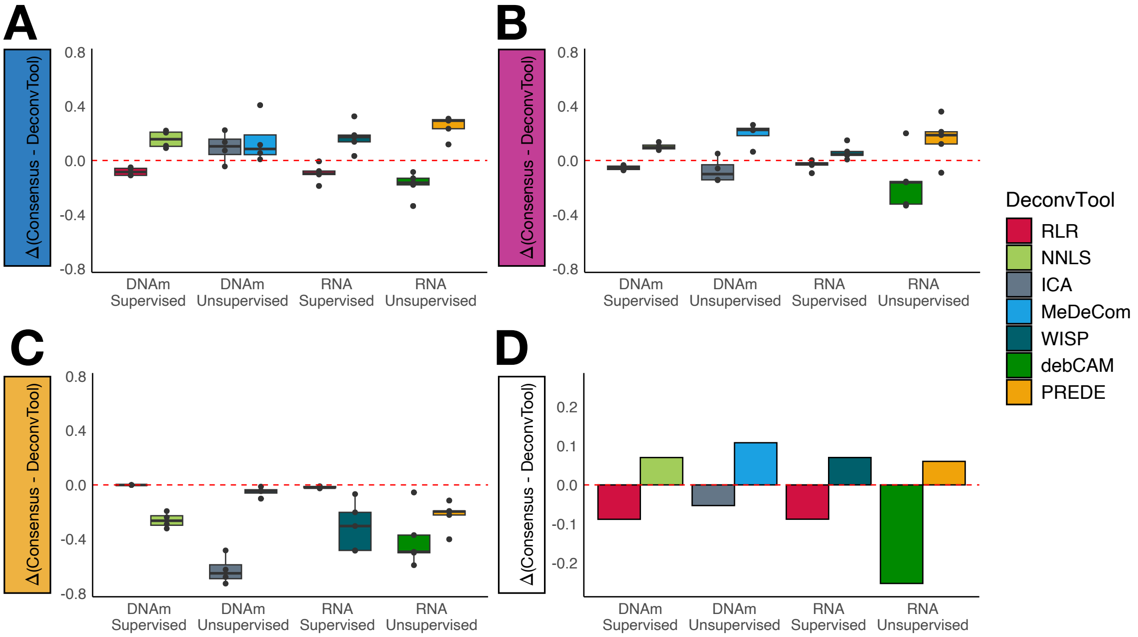

Fig. S21: Consensus approaches. They do not improve the performance of the deconvolution, in any combination of class and omic. The boxplots display on the y-axis the differences in normalised scores for each dataset between the consensus strategy and the two best methods in each setting. The raw performance is shown in panel **(A)**, the stability in panel **(B)** and the scalability in panel **(C)**. **(D)** Difference in overall score between the consensus strategy and the two best methods of each setting.
