# Supplementary material for: A robust workflow to benchmark deconvolution of multi-omic data
Source: Genome Biol. 2025 Dec 17;26:429. doi: 10.1186/s13059-025-03897-9 (PMC12713266; doi:10.1186/s13059-025-03897-9)
Supplement: Supplementary file 2 — Additional file 2. Supplementary methods. [file 13059_2025_3897_MOESM2_ESM.pdf]

A ROBUST WORKFLOW TO BENCHMARK DECONVOLUTION OF MULTI-OMIC DATA
ADDITIONAL FILE 2: SUPPLEMENTARY METHODS

1 Data

1.1 Simulations: model noise

We added a noise after convoluting the reference matrix T with the proportions matrix A to mimic the technical noise.

Technical noise for DNAm data For methylation data, we added a Gaussian noise with parameters $\mu = 0$, $\sigma = 3$. More precisely, we did the convolution $T \times A$, and converted the resulting matrix to M-values, using the formula $M_{ij} = log2(D_{ij}/(1-D_{ij}))$. Then we generated random numbers based on a Gaussian distribution with the *rnorm* R function, that we added to the M-values matrix. Finally, we revert to β-values using the formula $D_{ij} = 2^{M_{ij}}/(2^{M_{ij}} + 1)$.

Technical noise for RNA data For transcriptomic data, we used a Negative Binomial noise to add to the convolution $T \times A$ of size $f \times s$, following the same procedure as in:[10]

P is a matrix of size $f \times s$ with all its elements equal to 10^{-1}
Δ is a matrix of Gaussianly distributed numbers with parameters $\mu = 0$ and $\sigma = 1$, of size $f \times s$
c is the vector of the sums on the columns of $T \times A$: $c_j = \sum_i \sum_k T_{ik} A_{kj}$ of length s
M_0 is a matrix of size $f \times s$, defined by its elements $\mu^0_{ij} = \frac{\sum_k T_{ik} A_{kj}}{c_i} \times \frac{\sum_l c_l}{s}$
Σ_0 is a matrix defined as $\Sigma_0 = (1.8P + \frac{1}{\sqrt{M_0}}) \times \exp(\frac{\Delta}{2})$ with element-wise addition, division and multiplication
$S_1 = \frac{1}{\Sigma_0^2}$ with element-wise division
$S_2 = \frac{M_0}{S_1 + \epsilon}$ with element-wise addition and division and $\epsilon \approx 10^{-16}$
M is a matrix of size $f \times s$ of numbers drawn from Gamma distributions with parameters S_1 and S_2
The resulting expression matrix D is a matrix of numbers drawn from a Poisson distribution with parameter M

Copula-derived noise For the PaCL2 dataset, we have a matching *in vitro* dataset PaMIX: it contains the same cell types, sequenced in the same batch, and it has the same reference matrix. Based on PaMIX, we computed the empirical copula, that was then used to create a noise that takes into account dependencies between features.

Step 1: Compute the matrix of residuals $\epsilon_{ij} = D^{\text{PaMIX}}_{ij} - \sum_k T^{\text{PaMIX}}_{ik} \times A^{\text{PaMIX}}_{kj}$ for each omic (using M-values for methylation)
Step 2: Center and scale the residuals
Step 3: Compute pseudo-observations from the centered-scaled residuals, the matching empirical copula and draw randomly numbers from this copula with the R package `copula` (v1.1-6)
Step 4: Generate a Negative Binomial (for RNA) or a Gaussian (for DNAm) marginal by applying the corresponding quantile function on the vector of numbers drawn at step 3
Step 5: Add the copula-based noise to each omic, revert to β-values for DNAm data.

1.2 Single-cell RNAseq labelling

We used a public scRNAseq dataset[15] that we reannotated using a manually curated marker database. The labelling was performed using gene set enrichment analysis (GSEA) combined with a copy number variation (CNV)-based approach to identify cancer cells. The re-annotated dataset is available from Zenodo (DOI 10.5281/zenodo.14024478).

Data collection The Peng dataset was downloaded from the Genome Sequence Archive (GSA) under the accession number PRJCA001063, and only primary tumors were kept.[15] This dataset contains 35 samples (24 tumors and 11 controls) and 57530 cells. The preprocessing of the raw matrix and downstream analyses were performed using R

(version 4.0.2) and the `Seurat` package (v4.0.4). We used the default parameters for the first steps of dead cells removal, normalization, dimension reduction and clustering. We discared low quality cells and features with parameters min.cells = 3, min.features = 200, percent.mt $< 5\%$.

Literature curation of markers We generated a curated database of marker genes based on publications describing different pancreatic cell types in normal and/or tumoral tissues. We retrieved 214 stromal/immune and 142 epithelial markers from.[11] Markers of acinar, endocrine (α, β, γ and δ) and ductal cells were published in[3] and merged with markers from the Enrichr database.[6] We also added the ductal marker *PROM1* described in.[9] For tumoral markers, we separated classic- and basal-like tumor cells using markers published in at least two different studies.[2,13,7,12,16] Stromal cells were divided in three subcategories, fibroblasts/stellates, immune and endothelial cells. Fibroblasts/stellates markers were retrieved from.[8] Immune cells were further divided into 4 subpopulations: T and B cells, dendritic cells and monocytes/macrophages. The markers were defined based on 2 different publications.[4,5]

Clustering We normalized with normalization.method = "LogNormalize", did the PCA with 2000 variable genes, selected the first 10 PCs for the function *Seurat::FindNeighbors* and did the clustering with the function *FindClusters*, with resolution = 1. Cells were labelled with GSEA (see below). Unlabelled cells were reclustered with resolution = 0.5.

Cell labelling In order to separate neoplastic from normal epithelial cells, we relied on CNV estimation using the `InferCNV` package. The *InferCNV* function was used with cutoff = 0.1 and HMM = True. Cell labelling was performed using our curated database of markers. After the first clustering, we applied GSEA using the `fgsea` package: for each cluster, genes were ranked according to their average z-score and tested for gene set enrichment considering the markers from our curated database. We used an adjusted p-value cutoff of 0.1. Clusters with significant p-values for more than one type were considered as ambiguous and discarded. Clusters with no significant p-values were re-clustered with a lower resolution.

1.3 Creating an extra cell type

To create an extra cell type in the reference matrix, we first started by selecting all immune cell types T_{immune} in T. If we have more than one cell type, we computed the average profile by calculating the arithmetic mean for each row of T_{immune}. Finally we added noise, following the procedure described above. For methylation data, the parameters we used for the Gaussian distribution were $\mu = 0$ and $\sigma = 1$. For transcriptomic data, the parameters p and σ were kept the same.

1.4 Quantifying the heterogeneity of a dataset

We used two proxies to quantify a dataset's heterogeneity. First, we measured the mean of the Pearson correlations between all pairs of cell types in T, called "Mean R^2". Then, we computed the logarithm of the phenotypic volume V as defined in.[1] Briefly, it represents the volume occupied by the different cell types in the space of molecular features:

$$\log(V) = \log(\frac{|\Sigma|_+}{K-1}) = \log(\frac{\prod_{e=1}^{E} \lambda_e}{K-1}); \forall \lambda_e > 0$$

where Σ is the covariance matrix of T restricted to the $K-1$ most variable features, K is the number of cell types and λ_e are the non-zero eigenvalues of Σ.

We used in our analyses the composite parameter HG $= \log(V) \times R^{-2}$.

2 Deconvolution pipeline: methods

The RDS file containing genes' length for TPM normalization is available upon request to the corresponding authors. We specify below the functions and the non-default parameters that were used for each deconvolution method. As a reminder, NN stands for Non Negative and STO for Sum To One.

2.1 RNA methods

CIBERSORT was run with the source code downloaded from https://cibersortx.stanford.edu/csdownload.php with default parameters (v1.04) or, when specified, with the *EpiDISH::epidish* function (v2.18.0), setting "method" to

"CBS", or, when specified, with the source code downloaded from https://cibersortx.stanford.edu/csdownload.php with default parameters (v1.04).

DeconRNASeq was run with the *DeconRNASeq::DeconRNASeq* function with "use.scale = FALSE" and a further STO constraint (v1.44.0).

Elastic net was done via the *glmnet::glmnet* function with further explicit NN and STO constraints (v4.1-8).

FARDEEP and FARDEEP_sto were run with the *FARDEEP::fardeep* function, and proportions were retrieved from the "abs.beta" slot for FARDEEP, and "relative.beta" slot for FARDEEP_sto (v1.0.1).

InstaPrism was run by adapting the code available at https://github.com/humengying0907/InstaPrism/tree/master, and setting the number of cores to 32.

NNLS, OLS and SVR were run with the `granulator` package on TPM-normalized data with 32 cores, with further explicit NN and STO constraints (v1.10.0).

RLR was done with the *EpiDISH::epidish* function, setting "method" to "RPC" (v2.18.0).

WISP was run using the code available on https://github.com/cit-bioinfo/WISP, adding an additional STO constraint. The parameter scaling was set to 'scale' for the PaPB dataset, as recommanded by the authors of the method in case the reference and expression matrices are from two different technologies.

CDSeq, PREDE and debCAM were run with the same parameters as in https://github.com/bcm-uga/gedepir/blob/main/R/run_deconvolution.R, with a NN constraint for PREDE.

ICA was run with the *fastICA::fastICA* function with "maxit = 1000" and "tol = 1×10^{-9}", with a further STO constraint (v1.2-4).

NMF with performed with the *NMF::nmf* function on the matrix restricted to the features that have at least 1 count across all the samples, with a seed of 1 and method = "snmf/r", and a STO constraint (v0.26).

2.2 DNAm methods

CIBERSORT, NNLS and RLR were run as described for RNA methods.

epidishCP was run with the *EpiDISH::epidish* function, setting "method" to "CP".

debCAM, ICA and NMF were run as described above.

EDec was performed with the *EDec::run_edec_stage_1* function using all CpGs as informative loci, and with an additional NN constraint (v0.9).

InstaPrism was run as described above, with a pre-transformation of the data by multiplying the β-values by 1,000 and rounding the result.

RefFreeEWAS was run with the *RefFreeEWAS::RefFreeCellMix* function and additional NN and STO constraints (v2.2).

2.3 References of the reviews cited in Tables S1 and S2

**Review number**	**DOI**
1	10.1093/bib/bbac449
2	10.1093/bib/bbae234
3	10.1038/s43588-021-00038-7
4	10.2217/epi-2016-0153
5	10.1093/hmg/ddx275
6	10.1093/bib/bbac248
7	10.1016/j.csbj.2021.12.001

Reviews listed in Table S1

**Review number**	**DOI**
1	10.1016/j.coi.2013.09.015
2	10.1007/s00262-018-2150-z
3	10.1093/nar/gkae267
4	10.1093/bioadv/vbae048
5	10.1038/s41467-023-41385-5
6	10.1038/s41467-020-19015-1
7	10.1186/s13059-021-02290-6
8	10.1038/s41467-024-50618-0
9	10.1038/s41467-022-28655-4
10	10.1093/bioinformatics/btz363
11	10.1093/bib/bbu002

Reviews listed in Table S2

3 Ranking pipeline

3.1 Metrics preparation

We display in Table 1 an example of the primary metrics we can compute directly from the estimation of the proportions by a given method for each simulation. Of note, the time t is the real execution time divided by the number of samples. In case we have replicates, *i.e.* for *in silico* data, we can add secondary stability metrics, and we average across replicates for the primary metrics, as in Table 2. The time is also log-transformed since the execution times of all methods spanned several orders of magnitude:

$\mathrm{Time_{log}} = \log_{10}(1 + \mathrm{t})$

Replicate	RMSE	MAE	Pearson_Matrix	Pearson_CellType	Pearson_Sample	Time (s)
Sim1	0.21	0.33	0.87	Median = 0.70<br>Standard dev = 0.02	Median = 0.87<br>Standard dev = 0.03	12
Sim2	0.23	0.27	0.92	Median = 0.79<br>Standard dev = 0.01	Median = 0.85<br>Standard dev = 0.01	11
Sim3	0.18	0.40	0.86	Median = 0.83<br>Standard dev = 0.04	Median = 0.89<br>Standard dev = 0.02	13
Sim4	0.20	0.41	0.80	Median = 0.81<br>Standard dev = 0.03	Median = 0.90<br>Standard dev = 0.02	12

Table 1: Fake example of primary metrics.

Then we normalize the different metrics. We scale all metrics between 0 and 1, and we transform the scores such that 1 is always the best result, by inverting all metrics except the Pearson ones. For the normalization step, we first center all scores from all methods for a given metric and a given dataset by subtracting the arithmetic mean. Then we scale by dividing by the standard deviation and applying the distribution function (with the *pnorm* R function) of the normal distribution.

RMSE	MAE	Pearson_Matrix	Pearson_CellType	Pearson_Sample	Time (log)
Median = 0.21<br>Sd = 0.02	Median = 0.37<br>Sd = 0.07	Median = 0.87<br>Sd = 0.05	Median of medians = 0.80<br>Median of standard devs = 0.03	Median of medians = 0.88<br>Median of standard devs = 0.02	Median = 1.11<br>Sd = 0.03

Table 2: Fake example of averaged primary metrics along with secondary metrics.

The last step is to merge Pearson metrics into a single Pearson meta-score, separately for the metrics related to raw performance or to stability. We select first the averaged-normalized-transformed Pearson_Matrix, Pearson_CellType and Pearson_Sample metrics and calculate the arithmetic mean of those three scores for each method and dataset. We do the same for the standard deviation of the normalized-transformed Pearson_Matrix, Pearson_CellType and Pearson_Sample metrics. We end up with 8 metrics in case of replicates (4 otherwise), as shown in Table 3.

RMSE	MAE	Pearson	Time (log)
Norm median = 0.79<br>Norm Sd = 0.98	Norm median = 0.63<br>Norm sd = 0.93	Norm mean = 0.85<br>Norm sd = 0.97	Norm median = 0.82<br>Sd = 0.97

Table 3: Fake example of averaged-normalized-transformed scores after merging Pearson metrics.

3.2 Metrics renormalization to measure a dataset's easiness-of-deconvolution

Since we wanted to quantify how easy it was to deconvolve a specific dataset, we do not want to normalize scores as previously described. Indeed, this procedure is meant to avoid having a dataset with a large impact on the overall score by smoothing out differences between ease- and hard-to-deconvolve datasets. Here, we re-normalized each metric across all methods and all datasets simultaneously, instead of normalizing on a per dataset basis. Then we transformed the scores and merged all Pearson metrics. Finally, we computed the mean of the re-normalized aggregated scores for all methods for a dataset X to get a metric measuring dataset's easiness of deconvolution.

3.3 Metrics aggregation

The global aggregation procedure is broken down in three steps. The first step (i) consists in merging all metrics inside a category (raw performance, stability or scalability) and for a given dataset and method with a geometric mean as it is less influenced by outlier metrics than the arithmetic mean. We used the geometric mean whenever we wanted to mitigate the presence of possible outliers. The second step (ii) consists in merging, for a given dataset and method, the three categories with a weighted geometric mean. We used a weight of 1 for the raw performance, which we deemed to be the most important parameter to consider, and weights of 0.5 for stability and scalability. The last step (iii) is the final merging of the scores for one method for the different datasets with an arithmetic mean. The whole procedure is summarized in scheme 1.

3.4 Process S_{rank}

We have a ranking process that relies on computing ranks from the normalized-transformed scores before doing the global aggregation. For a fixed dataset and metric, we calculate the ranks of all methods with the R function *rank*, with the parameter "ties.method" set to "average" and the parameter "na.last" set to "keep". We then scale the ranks between 0 and 1 by dividing by the maximum rank, before going through the global aggregation described above.

3.5 Process S_{topsis}

For the topsis process, we use the normalized-transformed scores to compute TOPSIS scores that quantify how close a method is to the positive ideal solution (PIS) and how far it is from the negative ideal solution (NIS). In practice, a TOPSIS score for a given method and metric in a fixed dataset is calculated as follows: the PIS is defined as the maximum score observed across all methods for each metric, and the NIS as the minimum score. Then we calculate for each metric the Euclidean distance from a method to the PIS and NIS. Finally the TOPSIS score is the ratio $\frac{d_{\text{NIS}}}{d_{\text{NIS}}+d_{\text{PIS}}}$. Hence the TOPSIS score is restricted to the $[0, 1]$ interval and the higher it is, the closer the method is to the PIS. From the matrix of TOPSIS scores, we do the global aggregation to perform the process S_{topsis}.

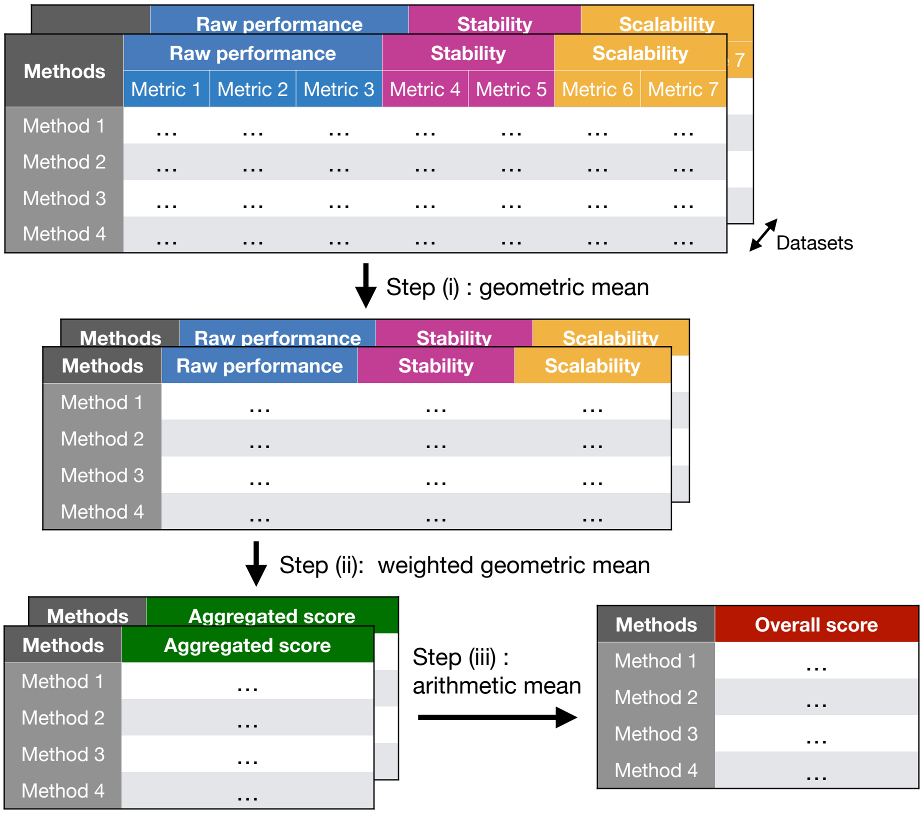

Scheme 1: Scheme of the three-steps global aggregation procedure.

3.6 Criteria for the evaluation of ranking processes

We were interested in evaluating the soundness of the ranking processes we designed. We quantified several empirical criteria as in:[14] the average rank, the condorcet rate and the generalization criterion. Let us briefly describe these, each one starting with the matrix of normalized-transformed scores $\mathbf{M}$ with the methods on the rows and the metrics on the columns. We calculated each criterion for each setting, which is a combination of the omic analysed and the class of the methods, supervised or unsupervised.

Average rank The average rank looks at the mean rank of the winner, across all metrics, elected by the ranking process S being evaluated. If x is the winner method, we first compute the matrix of ranks across all metrics $\mathbf{R}^{\mathbf{M}}$, then the mean rank of the winner $mean(\mathbf{R}^{\mathbf{M}}{}_{x,})$. Finally we normalize the result to obtain a value between 0 and 1, 1 being the best score:

$$1 - \frac{mean(\mathbf{R}^{\mathbf{M}}{}_{x,}) - 1}{J-1}$$

with J the number of metrics.

Condorcet rate Let us first define the Condorcet winner. The Condorcet winner is the method that would win across more than 50% of the metrics against all the other methods in a pairwise comparison. Hence the Condorcet rate is the rate at which the winner elected by our ranking process corresponds to the Condorcet winner in all pairwise comparisons.

Generalization criterion This criterion quantifies how generalizable the ranking process is, meaning how much it depends on a subset of metrics or not. The higher the criterion, the less it depends on specific metrics. It is intended to measure how much adding new metrics will perturb the ranking. Shortly, it is calculated for each ranking process as follows:

$$\text{Gen} = \frac{1}{|\mathcal{J}^{test}|} \sum_{j \in \mathcal{J}^{test}} \sigma(\mathbf{S}^{train}, \mathbf{S}^{test})$$

where σ is the Spearman correlation, $\mathcal{J}^{train}$ and $\mathcal{J}^{test}$ are disjoint sets of metrics, and $\mathbf{S}^{train}$ (resp. $\mathbf{S}^{test}$) are the ranks obtained from applying the ranking process S on the matrix $\mathbf{M}$ restricted to the metrics in $\mathcal{J}^{train}$ (resp. $\mathcal{J}^{test}$).

In our case, we excluded 10% of the metrics in the train set. Since we don't have many metrics, it led to having all metrics except one in the train set, and one metric in the test set, hence $|\mathcal{J}^{test}|= 1$. We excluded successively the first, second, ..., k-th, ..., J-th metric and computed the corresponding value Gen_k:

$\mathsf{Gen}_k = \sigma(\mathbf{S}^{-k}, \mathbf{S}^k)$

where $\mathbf{S}^{-k}$ (resp. $\mathbf{S}^k$) are the ranks obtained using all the metrics minus the k-th one (resp. using the k-th metric).

Finally, we get:

$\mathsf{Gen} = \frac{1}{J}\sum_{k=1}^{J} \mathsf{Gen}_k$

References

[1] Elham Azizi, Ambrose J. Carr, George Plitas, Andrew E. Cornish, Catherine Konopacki, Sandhya Prabhakaran, Juozas Nainys, Kenmin Wu, Vaidotas Kiseliovas, Manu Setty, Kristy Choi, Rachel M. Fromme, Phuong Dao, Peter T. McKenney, Ruby C. Wasti, Krishna Kadaveru, Linas Mazutis, Alexander Y. Rudensky, and Dana Pe'er. Single-Cell Map of Diverse Immune Phenotypes in the Breast Tumor Microenvironment. *Cell*, 174(5):1293–1308.e36, August 2018.

[2] Peter Bailey, David K. Chang, Katia Nones, Amber L. Johns, Ann-Marie Patch, Marie-Claude Gingras, David K. Miller, Angelika N. Christ, Tim J. C. Bruxner, Michael C. Quinn, Craig Nourse, L. Charles Murtaugh, Ivon Harliwong, Senel Idrisoglu, Suzanne Manning, Ehsan Nourbakhsh, Shivangi Wani, Lynn Fink, Oliver Holmes, Venessa Chin, Matthew J. Anderson, Stephen Kazakoff, Conrad Leonard, Felicity Newell, Nick Waddell, Scott Wood, Qinying Xu, Peter J. Wilson, Nicole Cloonan, Karin S. Kassahn, Darrin Taylor, Kelly Quek, Alan Robertson, Lorena Pantano, Laura Mincarelli, Luis N. Sanchez, Lisa Evers, Jianmin Wu, Mark Pinese, Mark J. Cowley, Marc D. Jones, Emily K. Colvin, Adnan M. Nagrial, Emily S. Humphrey, Lorraine A. Chantrill, Amanda Mawson, Jeremy Humphris, Angela Chou, Marina Pajic, Christopher J. Scarlett, Andreia V. Pinho, Marc Giry-Laterriere, Ilse Rooman, Jaswinder S. Samra, James G. Kench, Jessica A. Lovell, Neil D. Merrett, Christopher W. Toon, Krishna Epari, Nam Q. Nguyen, Andrew Barbour, Nikolajs Zeps, Kim Moran-Jones, Nigel B. Jamieson, Janet S. Graham, Fraser Duthie, Karin Oien, Jane Hair, Robert Grützmann, Anirban Maitra, Christine A. Iacobuzio-Donahue, Christopher L. Wolfgang, Richard A. Morgan, Rita T. Lawlor, Vincenzo Corbo, Claudio Bassi, Borislav Rusev, Paola Capelli, Roberto Salvia, Giampaolo Tortora, Debabrata Mukhopadhyay, Gloria M. Petersen, Australian Pancreatic Cancer Genome Initiative, Donna M. Munzy, William E. Fisher, Saadia A. Karim, James R. Eshleman, Ralph H. Hruban, Christian Pilarsky, Jennifer P. Morton, Owen J. Sansom, Aldo Scarpa, Elizabeth A. Musgrove, Ulla-Maja Hagbo Bailey, Oliver Hofmann, Robert L. Sutherland, David A. Wheeler, Anthony J. Gill, Richard A. Gibbs, John V. Pearson, Nicola Waddell, Andrew V. Biankin, and Sean M. Grimmond. Genomic analyses identify molecular subtypes of pancreatic cancer. *Nature*, 531(7592):47–52, March 2016.

[3] Maayan Baron, Adrian Veres, Samuel L. Wolock, Aubrey L. Faust, Renaud Gaujoux, Amedeo Vetere, Jennifer Hyoje Ryu, Bridget K. Wagner, Shai S. Shen-Orr, Allon M. Klein, Douglas A. Melton, and Itai Yanai. A Single-Cell Transcriptomic Map of the Human and Mouse Pancreas Reveals Inter- and Intra-cell Population Structure. *Cell Systems*, 3(4):346–360.e4, October 2016.

[4] Etienne Becht, Nicolas A. Giraldo, Laetitia Lacroix, Bénédicte Buttard, Nabila Elarouci, Florent Petitprez, Janick Selves, Pierre Laurent-Puig, Catherine Sautès-Fridman, Wolf H. Fridman, and Aurélien de Reyniès. Estimating the population abundance of tissue-infiltrating immune and stromal cell populations using gene expression. *Genome Biology*, 17(1):218, October 2016.

[5] Gabriela Bindea, Bernhard Mlecnik, Marie Tosolini, Amos Kirilovsky, Maximilian Waldner, Anna C. Obenauf, Helen Angell, Tessa Fredriksen, Lucie Lafontaine, Anne Berger, Patrick Bruneval, Wolf Herman Fridman, Christoph Becker, Franck Pagès, Michael R. Speicher, Zlatko Trajanoski, and Jérôme Galon. Spatiotemporal dynamics of intratumoral immune cells reveal the immune landscape in human cancer. *Immunity*, 39(4):782–795, October 2013.

[6] Edward Y. Chen, Christopher M. Tan, Yan Kou, Qiaonan Duan, Zichen Wang, Gabriela Vaz Meirelles, Neil R. Clark, and Avi Ma'ayan. Enrichr: interactive and collaborative HTML5 gene list enrichment analysis tool. *BMC bioinformatics*, 14:128, April 2013.

[7] Eric A. Collisson, Anguraj Sadanandam, Peter Olson, William J. Gibb, Morgan Truitt, Shenda Gu, Janine Cooc, Jennifer Weinkle, Grace E. Kim, Lakshmi Jakkula, Heidi S. Feiler, Andrew H. Ko, Adam B. Olshen, Kathleen L.

Danenberg, Margaret A. Tempero, Paul T. Spellman, Douglas Hanahan, and Joe W. Gray. Subtypes of pancreatic ductal adenocarcinoma and their differing responses to therapy. *Nature Medicine*, 17(4):500–503, April 2011.

[8] Ela Elyada, Mohan Bolisetty, Pasquale Laise, William F. Flynn, Elise T. Courtois, Richard A. Burkhart, Jonathan A. Teinor, Pascal Belleau, Giulia Biffi, Matthew S. Lucito, Santhosh Sivajothi, Todd D. Armstrong, Dannielle D. Engle, Kenneth H. Yu, Yuan Hao, Christopher L. Wolfgang, Youngkyu Park, Jonathan Preall, Elizabeth M. Jaffee, Andrea Califano, Paul Robson, and David A. Tuveson. Cross-Species Single-Cell Analysis of Pancreatic Ductal Adenocarcinoma Reveals Antigen-Presenting Cancer-Associated Fibroblasts. *Cancer Discovery*, 9(8):1102–1123, August 2019.

[9] Martin Enge, H. Efsun Arda, Marco Mignardi, John Beausang, Rita Bottino, Seung K. Kim, and Stephen R. Quake. Single-Cell Analysis of Human Pancreas Reveals Transcriptional Signatures of Aging and Somatic Mutation Patterns. *Cell*, 171(2):321–330.e14, October 2017.

[10] Haijing Jin and Zhandong Liu. A benchmark for RNA-seq deconvolution analysis under dynamic testing environments. *Genome Biology*, 22(1):102, April 2021.

[11] Carlo Maurer, Sam R. Holmstrom, Jing He, Pasquale Laise, Tao Su, Aqeel Ahmed, Hanina Hibshoosh, John A. Chabot, Paul E. Oberstein, Antonia R. Sepulveda, Jeanine M. Genkinger, Jiapeng Zhang, Alina C. Iuga, Mukesh Bansal, Andrea Califano, and Kenneth P. Olive. Experimental microdissection enables functional harmonisation of pancreatic cancer subtypes. *Gut*, 68(6):1034–1043, June 2019.

[12] Richard A. Moffitt, Raoud Marayati, Elizabeth L. Flate, Keith E. Volmar, S. Gabriela Herrera Loeza, Katherine A. Hoadley, Naim U. Rashid, Lindsay A. Williams, Samuel C. Eaton, Alexander H. Chung, Jadwiga K. Smyla, Judy M. Anderson, Hong Jin Kim, David J. Bentrem, Mark S. Talamonti, Christine A. Iacobuzio-Donahue, Michael A. Hollingsworth, and Jen Jen Yeh. Virtual microdissection identifies distinct tumor- and stroma-specific subtypes of pancreatic ductal adenocarcinoma. *Nature Genetics*, 47(10):1168–1178, October 2015.

[13] Rémy Nicolle, Yuna Blum, Laetitia Marisa, Celine Loncle, Odile Gayet, Vincent Moutardier, Olivier Turrini, Marc Giovannini, Benjamin Bian, Martin Bigonnet, Marion Rubis, Nabila Elarouci, Lucile Armenoult, Mira Ayadi, Pauline Duconseil, Mohamed Gasmi, Mehdi Ouaissi, Aurélie Maignan, Gwen Lomberk, Jean-Marie Boher, Jacques Ewald, Erwan Bories, Jonathan Garnier, Anthony Goncalves, Flora Poizat, Jean-Luc Raoul, Veronique Secq, Stephane Garcia, Philippe Grandval, Marine Barraud-Blanc, Emmanuelle Norguet, Marine Gilabert, Jean-Robert Delpero, Julie Roques, Ezequiel Calvo, Fabienne Guillaumond, Sophie Vasseur, Raul Urrutia, Aurélien de Reyniès, Nelson Dusetti, and Juan Iovanna. Pancreatic Adenocarcinoma Therapeutic Targets Revealed by Tumor-Stroma Cross-Talk Analyses in Patient-Derived Xenografts. *Cell Reports*, 21(9):2458–2470, November 2017.

[14] Adrien Pavao, Michael Vaccaro, and Isabelle Guyon. Judging competitions and benchmarks: a candidate election approach, 2021. Paper presented at ESANN 2021.

[15] Junya Peng, Bao-Fa Sun, Chuan-Yuan Chen, Jia-Yi Zhou, Yu-Sheng Chen, Hao Chen, Lulu Liu, Dan Huang, Jialin Jiang, Guan-Shen Cui, Ying Yang, Wenze Wang, Dan Guo, Menghua Dai, Junchao Guo, Taiping Zhang, Quan Liao, Yi Liu, Yong-Liang Zhao, Da-Li Han, Yupei Zhao, Yun-Gui Yang, and Wenming Wu. Single-cell RNA-seq highlights intra-tumoral heterogeneity and malignant progression in pancreatic ductal adenocarcinoma. *Cell Research*, 29(9):725–738, September 2019.

[16] Francesco Puleo, Rémy Nicolle, Yuna Blum, Jérôme Cros, Laetitia Marisa, Pieter Demetter, Eric Quertinmont, Magali Svrcek, Nabila Elarouci, Juan Iovanna, Denis Franchimont, Laurine Verset, Maria Gomez Galdon, Jacques Devière, Aurélien de Reyniès, Pierre Laurent-Puig, Jean-Luc Van Laethem, Jean-Baptiste Bachet, and Raphaël Maréchal. Stratification of Pancreatic Ductal Adenocarcinomas Based on Tumor and Microenvironment Features. *Gastroenterology*, 155(6):1999–2013.e3, December 2018.
